# Contribution of HLA and KIR Alleles to Systemic Sclerosis Susceptibility and Immunological and Clinical Disease Subtypes

**DOI:** 10.3389/fgene.2022.913196

**Published:** 2022-06-08

**Authors:** Aimee L. Hanson, Joanne Sahhar, Gene-Siew Ngian, Janet Roddy, Jennifer Walker, Wendy Stevens, Mandana Nikpour, Shervin Assassi, Susanna Proudman, Maureen D. Mayes, Tony J. Kenna, Matthew A. Brown

**Affiliations:** ^1^ Department of Medicine, University of Queensland, Brisbane, QLD, Australia; ^2^ Department of Medicine, University of Cambridge, Cambridge, United Kingdom; ^3^ Department of Medicine, Clayton and Monash Health, Monash University, Melbourne, VIC, Australia; ^4^ Department of Rheumatology, Royal Perth Hospital, Perth, WA, Australia; ^5^ Rheumatology Unit, Flinders Medical Centre, Adelaide, SA, Australia; ^6^ Department of Medicine, University of Melbourne, St Vincent’s Hospital, Melbourne, VIC, Australia; ^7^ Department of Rheumatology, St Vincent’s Hospital, Melbourne, VIC, Australia; ^8^ Division of Rheumatology, University of Texas, Houston, TX, United States; ^9^ Rheumtology Unit, Royal Adelaide Hospital, Adelaide, SA, Australia; ^10^ Discipline of Medicine, University of Adelaide, Adelaide, SA, Australia; ^11^ Institute of Health and Biomedical Innovation, Queensland University of Technology, Brisbane, QLD, Australia; ^12^ Genomics England, Charterhouse Square, London, United Kingdom; ^13^ Department of Medical and Molecular Genetics, Faculty of Life Sciences and Medicine, King’s College London, London, United Kingdom

**Keywords:** systemic sclerosis (scleroderma), HLA association and disease, human disease genetics, killer immunoglobulin like receptor (KIR), immunogenetics

## Abstract

Systemic sclerosis (SSc) is an autoinflammatory, fibrotic condition of unknown aetiology. The presence of detectable autoantibodies against diverse nuclear antigens, as well as strong HLA associations with disease, suggest autoimmune involvement, however the links between endogenous and exogenous risk factors and SSc pathology remain undetermined. We have conducted a genetic analysis of *HLA* inheritance in two independent and meta-analysed cohorts of 1,465 SSc cases and 13,273 controls, including stratified association analyses in clinical and autoantibody positive subgroups of disease. Additionally, we have used patient genotypes to impute gene dosages across the *KIR* locus, encoding paired activating and inhibitory lymphocyte receptors for Class I HLA ligands, to conduct the largest analysis of *KIR-HLA* epistatic interactions in SSc to date. We confirm previous Class II HLA associations with SSc risk and report a new Class I association with haplotype HLA-B*44:03-HLA-C*16:01 at genome-wide significance (GWS). We further report statistically significant *HLA* associations with clinical and serological subtypes of disease through direct case-case comparison, and report a new association of HLA-DRB1*15:01, previously shown to bind topoisomerase-1 derived peptides, with anti-topoisomerase (ATA) positive disease. Finally, we identify genetic epistasis between KIRs and HLA class I ligands, suggesting genetic modulation of lymphocyte activation may further contribute to an individual’s underlying disease risk. Taken together, these findings support future functional investigation into endogenous immunological and environmental stimuli for disrupted immune tolerance in SSc.

## Introduction

Systemic sclerosis (SSc; also known as scleroderma), is a connective-tissue disease of heterogeneous clinical presentation, characterised by a complex interplay between autoinflammatory and autoimmune processes, tissue fibrosis and vascular injury. Pathophysiology associated with SSc has been attributed to uncontrolled inflammation and associated activation of tissue resident fibroblasts, excessive collagen production and extracellular matrix remodelling (LeRoy, 1974). Two major subsets of disease are differentiated by the extent of skin and organ involvement. Diffuse cutaneous SSc (dcSSc) is characterised by extensive skin fibrosis and increased mortality associated with severe internal organ pathology. Alternatively, in limited cutaneous disease (lcSSc), skin involvement is typically restricted to the face and distal extremities, and vascular complications predominate. Investigated triggers of the inflammatory, fibrotic cascade in SSc suggest a role for a provocative environmental exposure, ranging from occupational chemicals (Sluis-Cremer et al., 1985; Kettaneh et al., 2007) to microbial infection (Maul et al., 1989; Lunardi et al., 2006), acting upon a background of heightened genetic predisposition. Albeit, the precise series of exogenous and endogenous circumstances that culminate in disease, and primary differentiators of clinical progression, remain unknown.

Anti-nuclear antibodies (ANA) targeting a range of cellular proteins are detected in the sera of up to 95% of SSc patients (Beck et al., 1963; Mierau et al., 2011), some of which have diagnostic utility in distinguishing SSc from other connective tissue diseases. Of the most common, anti-centromere antibodies (ACA), seen in 20–30% of all SSc cases, demonstrate reactivity against centromeric nucleoproteins such as the DNA-binding protein Centromere Protein B (CENP-B), and are predictive of limited disease with lower frequency of pulmonary fibrosis and associated mortality (Steen et al., 1988; Ho and Reveille, 2003). Anti-Scl70 antibodies directed against an epitope of the topoisomerase enzyme (abbreviated anti-topoisomerase, ATA; seen in 15–20% of SSc) are conversely predictive of diffuse disease and found in ∼45% of those who progress with lung complications (Reveille et al., 2003). Additionally, ATAs are associated with severity and development of interstitial lung disease (ILD), the leading cause of SSc mortality, in both limited and diffuse disease (Assassi et al., 2010; Jandali et al., 2022). A third and heterogeneous group of anti-nucleolar antibodies (ANoA; reported in 15–40% of patients) target, among other autoantigens, exosome, and ribonuclease components and several RNA polymerases (RNAP), with anti-RNAP antibodies demonstrating a strong association with heart failure and increased mortality in dcSSc (Jacobsen et al., 2001). Intriguingly, the co-occurrence of multiple ANAs of unique specificity in a single patient is rare. Their near mutual exclusivity suggests a unique interplay between genetic and environmental factors in the onset and progression of distinct clinical subclasses of disease, though whether ANA-associated autoimmunity is a cause or consequence of pathological processes in SSc remains undetermined.

The association of allelic variation across the class II human leukocyte antigen (HLA) locus with SSc risk is well established, supporting a functional role for CD4 T cell mediated autoantibody production in response to HLA class II restricted autoantigens in genetically susceptible individuals. Different HLA alleles demonstrate disease association when tested in lcSSc or dcSSc cohorts, or those stratified by autoantibody profile, implying that genetic heterogeneity significantly underpins the clinical heterogeneity of this disease. The strong risk association of HLA-DRB1*11:04 and HLA-DPB1*13:01 with SSc has been reported in multiple ethnic groups, enriched specifically in ATA + patients (Fanning et al., 1998; Arnett et al., 2010; Gourh et al., 2020; Acosta-Herrera et al., 2021). Conversely, HLA-DQA1*01:01/4 and HLA-DQB1*05 have been associated with risk of disease in ACA + Caucasians and Hispanics (Fanning et al., 1998; Reveille et al., 2001), and protective associations with HLA-DRB1*07:01 in this serological subgroup are described in multiple European cohorts (Zochling et al., 2014; Gourh et al., 2020; Acosta-Herrera et al., 2021).

Although less frequently reported, HLA class I associations with SSc suggest an additional role for CD8^+^ T cell mediated autoimmunity in disease. Early studies show enrichment of the HLA-A*09 subgroup allele HLA-A*24 in dcSSc, and HLA-A*30 and HLA-A*32 risk associations have also been reported in Caucasian and Brazilian populations, the former associated with risk of pulmonary hypertension and pulmonary fibrosis in SSc patients (Gladman et al., 2005; Del Rio et al., 2013). A recent intensive analysis of 9,095 SSc patients identified a significant disease risk association with HLA-B*08:01, independent of those observed with HLA Class II alleles (Acosta-Herrera et al., 2021). Increased CD8^+^ T cell clonality observed in the lungs and blood of SSc patients suggests proliferation in response to an immunogenic antigen (Yurovsky et al., 1996; Servaas et al., 2021), and activated CD8^+^ T cells have been identified in the skin, and isolated from the fibrotic lungs of SSc patients, where they express pro-fibrotic cytokines which stimulate fibroblast proliferation and collagen production (Atamas et al., 1999; Li et al., 2017). A summary of the findings from large-scale cohorts reporting classical HLA associations with SSc and disease subgroups can be found in Supplementary Table S1.

Additional to their role in antigen presentation, Class I HLA ligands are recognised by killer immunoglobulin-like receptors (KIRs) expressed on NK and CD8^+^ T cells, with roles in buffering lymphocyte activation and safeguarding against innate NK killing activity. Copy number variable haplotypes containing diverse combinations of 15 unique KIR genes encode activating (KIR2DS and KIR3DS) and inhibitory (KIR2DL and KIR3DL) receptors that engage specific subtypes of HLA Class I alleles. Canonically, KIR3DL1 is an inhibitory receptor for HLA-Bw4 ligands, a subgroup of HLA-A and B alleles carrying a recognised amino acid motif at positions 77–83 in the *a*-helix (Gumperz et al., 1996). The strength of this inhibitory interaction is governed by position 80 of the HLA Class I allele, being strongest for those alleles carrying isoleucine (Ile80), and weaker for those carrying threonine (The80), at position 80. Conversely, HLA-Bw6 alleles, lacking the Bw4 motif, are not known to serve as KIR ligands. KIR2DL2 and 2DL3 recognise the HLA-C1 family of alleles characterised by a position 80 asparagine, and KIR2DL1 engages members of the HLA-C2 allelic family, which carry a position 80 lysine (Winter et al., 1998) (Supplementary Table S2 lists major HLA-C, -Bw4 and -Bw6 family members). Ligands that engage activating KIRs are less well defined and extend beyond class I HLA to other surface markers of cell stress. However, HLA-dependent, peptide-specific activation of NK cells through KIR2DS1 recognition of HLA-C2 has been demonstrated *in vitro* (Chewning et al., 2007), and KIR3DS1 has been shown to engage HLA-B*57 (HLA-Bw4 subclass) bound HIV-derived peptides to activate NK killing responses (O'Connor et al., 2015). The exacerbated diversity attributed to copy number and allelic hyper-polymorphism across both the *KIR* genes and the combination of HLA ligands inherited imparts extensive variability in immunologic potential across the human population. Accordingly, *KIR-HLA* associations have been reported to play a role in genetic risk for many immune-mediated and infectious diseases (Khakoo et al., 2004; Pellett et al., 2007; Fusco et al., 2010; Hanson et al., 2020). Increased frequency of activating receptor *KIR2DS1* in SSc patients, particularly in those who lack a canonical HLA-C2 ligand for the paired inhibitory receptor *KIR2DL1,* has been reported (Pellett et al., 2007). Further studies report that lack of *KIR2DL2* inheritance increases SSc risk (Salim et al., 2010), and that coinheritance of canonical inhibitory receptor-ligand pair *KIR3DL1-HLA-Bw4*(The80) protects from disease (Mahmoudi et al., 2017), suggesting that activating KIR inheritance in the absence of compatible inhibitory interacting pairs may result in poorly restrained NK and T-cell activation and associated damaging inflammation.

Here we present the findings of an extensive investigation into *HLA* and *KIR* inheritance in two independent cohorts of SSc patients, including meta-analysis of 1,465 SSc cases and 13,273 controls. We present stratified *HLA*-association analyses in clinical and autoantibody positive SSc subgroups to dissect the genetic contribution of HLA alleles to unique serological and clinical manifestations of disease. We further impute *KIR* gene content information from participant genotypes to conduct the largest analysis of *KIR-HLA* coinheritance in this disease to date, and the first to address epistatic KIR interactions with both HLA subtypes and alleles in SSc. It is hoped that more thorough delineation of the genetic contributors to SSc will orient future research into the precise underlying triggers and immunological mechanisms of disease, and inform the development of targeted treatments to reduce the associated morbidity and mortality of those diagnosed.

## Materials and Methods

### Study Cohorts

Two independent cohorts of genotyped SSc patients and ethnically matched controls were used to test for genetic associations with disease and clinical disease subgroups across the *HLA* and *KIR* loci. Following quality filtering as described below, Cohort 1 comprised 503 Australian SSc patients recruited by the Australian Scleroderma Interest Group (ASIG), and Cohort 2 comprised 962 SSc patients from the United States of America, a subset of a previously published discovery cohort (Mayes et al., 2014). A total of 13,858 healthy controls, originally recruited by the International Genetics of Ankylosing Spondylitis (IGAS) Consortium as previously reported (Cortes et al., 2013), were split evenly between both SSc cohorts, with 6,632 and 6,641 controls remaining in Cohorts 1 and 2 respectively following quality filtering. Meta-analysis was performed in a combined cohort of 1,465 SSc cases and 13,273 controls. All patients met the American College of Rheumatology criteria for clinical diagnosis of SSc (Subcommittee for Scleroderma Criteria of the American Rheumatism Association Diagnostic and Therapeutic Criteria Committee, 1980), or the Medsger criteria for limited SSc (LeRoy and Medsger, 2001). Clinical metadata, including limited (lcSSc) or diffuse (dcSSc) disease classification, and ACA, ATA and ANoA autoantibody status (present/absent) was available for 490 Cohort 1 patients, and all 962 of Cohort 2 (classification frequencies shown in Supplementary Figure S1). Written informed consent was obtained from all participants, with research ethics approval granted by the relevant ethics committee at each participating centre.

### Genotyping and Sample Filtering

All SSc cases and controls were genotyped on the Illumina Immunochip array, which has high density SNP coverage of the leukocyte receptor complex at chromosome 19q13.4, where the *KIR* and *LILR* genes are encoded (Cortes and Brown, 2011). Sample genotypes were merged with genotypes from HapMap reference human populations and 17,374 common autosomal SNPs outside regions of long-range linkage disequilibrium (LD) were used to conduct principal component analysis (PCA) with shellfish (https://www.stats.ox.ac.uk/∼davison/software/shellfish/shellfish.php) for ethnicity assessment. Only patients and controls falling within plus or minus 5 standard deviations from the mean of the European sample cluster were retained. Principal components (PC) were recalculated for the filtered European participants and the first ten PCs fitted as covariates in all regression models to correct for remaining population stratification.

### HLA Imputation

Imputation of 268 classical HLA alleles to four-digit resolution was performed using HLA*IMP:03 (http://imp.science.unimelb.edu.au/hla/) (Motyer et al., 2016). The estimated imputation accuracy of Class I and II loci ranged between 95% (HLA-DRB1) and 99.84% (HLA-DPA1), with all alleles with a minor allele frequency >1% imputed with a mean estimated accuracy of 95.5% (IQR 94.7–98.8%). Consequently, no posterior probability threshold was applied to imputed HLA allele calls. The HLA subclass of each Class I allele (HLA-Bw4, Bw6, C1 or C2) was assigned based on known allele groupings (Supplementary Table S2) and the number of alleles carrying HLA-Bw4 (total), HLA-Bw4(I80), HLA-Bw4(T80), HLA-C1 or HLA-C2 motifs were summed for each individual for use in statistical analysis.

### KIR Imputation

A total of 265 and 224 SNPs spanning the *KIR* locus (Chr19: 59,793,991–60,190,556, Hg18) were available for SSc patients in Cohort 1 and Cohort 2 respectively, owing to independent genotype quality filtering prior to data acquisition. Matching variant positions were extracted from the paired controls of each cohort to minimise bias in *KIR* imputation accuracy, and SNP minor allele frequency was confirmed to closely match that of the KIR*IMP reference cohort (Vukcevic et al., 2015). Imputation of gene dosages across 14 copy-number variable *KIR* genes (*KIR2DP1, 2DS1, 2DS2, 2DS3, 2DS4, 2DS5, 2DL1, 2DL2, 2DL3, 2DL4, 2DL5, 3DP1, 3DL1, 3DS1*) was performed separately for Cohort 1 and 2 by passing phased SNP haplotypes to KIR*IMP (http://imp.science.unimelb.edu.au/kir/) (Vukcevic et al., 2015). Isoforms of *KIR3DL1*, with variable inclusion of exons 4 or 9, were distinguished by the imputation algorithm, but only total *KIR3DL1* gene dosage was used in analyses throughout. Presence or absence of a 22bp deletion in the *KIR2DS4* gene was denoted by *KIR2DS4DEL* and *KIR2DS4WT* dosages respectively, with *KIR2DS4TOTAL* capturing summed gene dosage. The posterior probability of *KIR* imputation accuracy was comparable across patients and controls from both study cohorts (Supplementary Figure S2). *KIR* genes present on rarer and more copy-number variable haplotypes (*KIR2DP1, 2DL1, 2DL5,* and *2DS3*) showed reduced imputation accuracy (Supplementary Figure S2), and consequently individuals predicted to carry rare haplotypes exhibited reduced haplotype posterior probability scores (as designated by the *KIRhaplotype* metric returned from the KIR*IMP software). Statistical analyses were conducted on both the full complement of imputed haplotypes, and those imputed above range of *KIRhaplotype* thresholds (0.5–0.9). Imputation thresholding skewed represented haplotypes toward those more common in the population (and more accurately imputed) but did not have a considerable effect on the results of *KIR-HLA* interaction analyses, which are gene not haplotype based, save for a reduction in statistical power. Thus, no posterior probability cut-off was applied to imputed haplotypes in the analyses presented here. To validate imputed haplotype calls on the population level, imputed *KIR* gene frequencies and percent prevalence across Cohorts 1 and 2 were compared to those reported for European populations in the Allele Frequency Net Database (Gonzalez-Galarza et al., 2020) (Supplementary Figure S3). *KIR* gene content haplotypes were annotated in accordance with the KIR*IMP reference cohort and imputed haplotype frequencies were compared to reference cohort frequencies derived from 793 nuclear families from the US and United Kingdom (Jiang et al., 2012) (Supplementary Figure S4).

### Statistical Analyses

Genotype associations with disease (or disease subgroup) status were assessed using logistic regression with the glm()function in R (R Core Team, 2021) for both independent and meta-analysis cohorts. The first 10 principal components capturing population genetic diversity (as detailed above) were included as covariates in each model. The association of disease status with HLA alleles at 4-digit resolution was assessed under a dominant inheritance model (allele absent = 0, heterozygote or homozygote = 1), and *KIR* gene dosage under both a dominant and recessive inheritance model (allele absent or heterozygote = 0, homozygote = 1), eg:

$$\begin{array}{l} \mathrm{glm}(\mathrm{status}∼\mathrm{allele}\mathrm{.count}+\mathrm{PC}1+\mathrm{PC}2+\mathrm{PC}3+\mathrm{PC}4+\mathrm{PC}5\\ +\mathrm{PC}6+\mathrm{PC}7+\mathrm{PC}8+\mathrm{PC}9+\mathrm{PC}10,\mathrm{family}\\ =\mathrm{binomial}\left(\mathrm{link}=\mathrm{logit}\right)) \end{array}$$

Iterative conditional analysis was used to dissect independent HLA associations across the locus exhibiting strong LD, in the combined meta-analysis cohort only. Here, the most significant disease-associated allele was added as a covariate to the logistic regression and association analyses repeated sequentially until no HLA allele showed a disease association below the GWS threshold p<5 × 10^−8^. Pairwise conditional analysis was conducted by conditioning each disease associated allele upon every other in a pairwise fashion, eg:

$$\begin{array}{l} \mathrm{glm}(\mathrm{status}∼\mathrm{allele}.\mathrm{count}.1+\mathrm{allele}.\mathrm{count}.2+\mathrm{PC}1+\mathrm{PC}2+\mathrm{PC}3\\ +\mathrm{PC}4+\mathrm{PC}5+\mathrm{PC}6+\mathrm{PC}7+\mathrm{PC}8+\mathrm{PC}9+\mathrm{PC}10,\mathrm{family}\\ =\mathrm{binomial}\left(\mathrm{link}=\mathrm{logit}\right)) \end{array}$$

HLA associations were also assessed in clinical subgroups of lcSSc and dcSSc patients, both by comparing allele frequencies in each to healthy controls, and between disease states, using logistic regression as detailed above. HLA allele frequencies in ANA positive patients (split into subsets of ACA, ATA and ANoA positive disease) were compared to the corresponding autoantibody negative patient cohort. The interaction between *KIR* gene and HLA subclass carriage in SSc was assessed by inclusion of an interaction term in the logistic regression, treating *KIR* and *HLA* inheritance as either dominant or recessive in every combination (i.e. KIR_DOM_ x HLA_DOM_, KIR_DOM_ x HLA_REC_, KIR_REC_ x HLA_DOM_, KIR_REC_ x HLA_REC_), eg:

$$\begin{array}{l} \text{glm}(\text{status∼HLA}\text{.count}+\text{KIR}\text{.count}+\text{HLA}\text{.count*KIR}\text{.count}\\ +\text{PC1}+\text{PC2}+\text{PC3}+\text{PC4}+\text{PC5}+\text{PC6}+\text{PC7}+\text{PC8}+\text{PC9}\\ +\text{PC10,family}\\ =\text{binomial(link=logit)}) \end{array}$$

Due to the large number of statistical tests conducted to assess epistatic interaction between these loci, only those KIR-HLA interactions achieving nominal significance (p_int_ <0.05) in the meta-analysis cohort, and occurring between a *KIR* and a disease associated class I HLA ligands, are reported in the main text, with all findings between biologically validated receptor-ligand pairs included as supplementary data as referenced below.

## Results

### HLA Associations With SSc

Class I and II HLA associations with SSc were assessed using logistic regression, under a dominant inheritance model. Applying a GWS threshold of p<5 × 10^−8^, significant disease-risk associations were observed with HLA-DRB1*11:04 (OR = 2.81, *p* = 2.3 × 10^−25^) and HLA-DPB1*13:01 (OR = 2.20, *p* = 1.8 × 10^−12^), and protective associations with HLA-DRB1*07:01 (OR = 0.52, *p* = 1.5 × 10^−17^), HLA-DQA1*02:01 (OR = 0.53, *p* = 3.5 × 10^−17^), HLA-DQB1*02:02 (OR = 0.49, *p* = 1.7 × 10^−15^), HLA-DRB4*01:01 (OR = 0.54, *p* = 9.3 × 10^−13^), HLA-B*44:03 (OR = 0.45, *p* = 1.8 × 10^−10^), and HLA-C*16:01 (OR = 0.44, *p* = 6.2 × 10^−9^) in the meta-analysis cohort. A suggestive association was also observed with HLA-DQA1*05:01 (OR = 1.34, *p* = 1.7 × 10^−7^). HLA disease-association signals detected in the meta-analysis cohort were supported in both independent study cohorts 1 and 2, six of which reached GWS in the latter and larger of the two (Table 1). The extended table of HLA allele frequencies and associations for all three cohorts is provided in Supplementary Table S3).

**TABLE 1:** HLA associations with SSc

	SSc vs. control																	
	Meta-Analysis cohort						Cohort 1						Cohort 2					
HLA allele	SSc prop.(Count)		CO prop.(Count)		OR	*p*	SSc prop.(Count)		CO prop.(Count)		OR	*p*	SSc prop.(Count)		CO prop.(Count)		OR	*p*
DRB1*11:04	0.119	(174/1465)	0.035	(459/13273)	2.81	2.3x10^−25^	0.099	(50/503)	0.035	(230/6632)	2.36	6.4x10^−7^	0.129	(124/962)	0.034	(229/6641)	3.08	1.3x10^−19^
DRB1*07:01	0.152	(223/1465)	0.257	(3406/13273)	0.52	1.5 x10^−17^	0.157	(79/503)	0.262	(1737/6632)	0.52	2.5x10^−7^	0.150	(144/962)	0.251	(1669/6641)	0.53	4.4x10^−11^
DQA1*02:01	0.153	(224/1465)	0.256	(3397/13273)	0.53	3.5 x10^−17^	0.159	(80/503)	0.261	(1733/6632)	0.53	4.8x10^−7^	0.150	(144/962)	0.251	(1664/6641)	0.53	5.4x10^−11^
DQB1*02:02	0.102	(149/1465)	0.186	(2468/13273)	0.49	1.7 x10^−15^	0.103	(52/503)	0.193	(1282/6632)	0.47	5.9x10^−7^	0.101	(97/962)	0.179	(1186/6641)	0.51	2.4x10^−9^
DRB4*01:01	0.111	(162/1465)	0.188	(2491/13273)	0.54	9.3 x10^−13^	0.109	(55/503)	0.194	(1289/6632)	0.50	2.6x10^−6^	0.111	(107/962)	0.181	(1202/6641)	0.57	2.9x10^−7^
DPB1*13:01	0.076	(111/1465)	0.033	(444/13273)	2.20	1.8 x10^−12^	0.068	(34/503)	0.035	(231/6632)	1.85	0.001	0.080	(77/962)	0.032	(213/6641)	2.50	7.5x10^−11^
B*44:03	0.050	(73/1465)	0.105	(1397/13273)	0.45	1.8 x10^−10^	0.066	(33/503)	0.110	(728/6632)	0.57	0.002	0.042	(40/962)	0.101	(669/6641)	0.40	4.9x10^−8^
C*16:01	0.038	(55/1465)	0.082	(1090/13273)	0.44	6.2 x10^−9^	0.050	(25/503)	0.086	(573/6632)	0.55	0.004	0.031	(30/962)	0.078	(517/6641)	0.39	9.1x10^−7^
DQA1*05:01	0.508	(744/1465)	0.421	(5583/13273)	1.34	1.7 x10^−7^	0.509	(256/503)	0.414	(2747/6632)	1.40	0.0003	0.507	(488/962)	0.427	(2836/6641)	1.29	0.0003

CO = control, OR = odds ratio, p = *p*-value.

**FIGURE 1:**
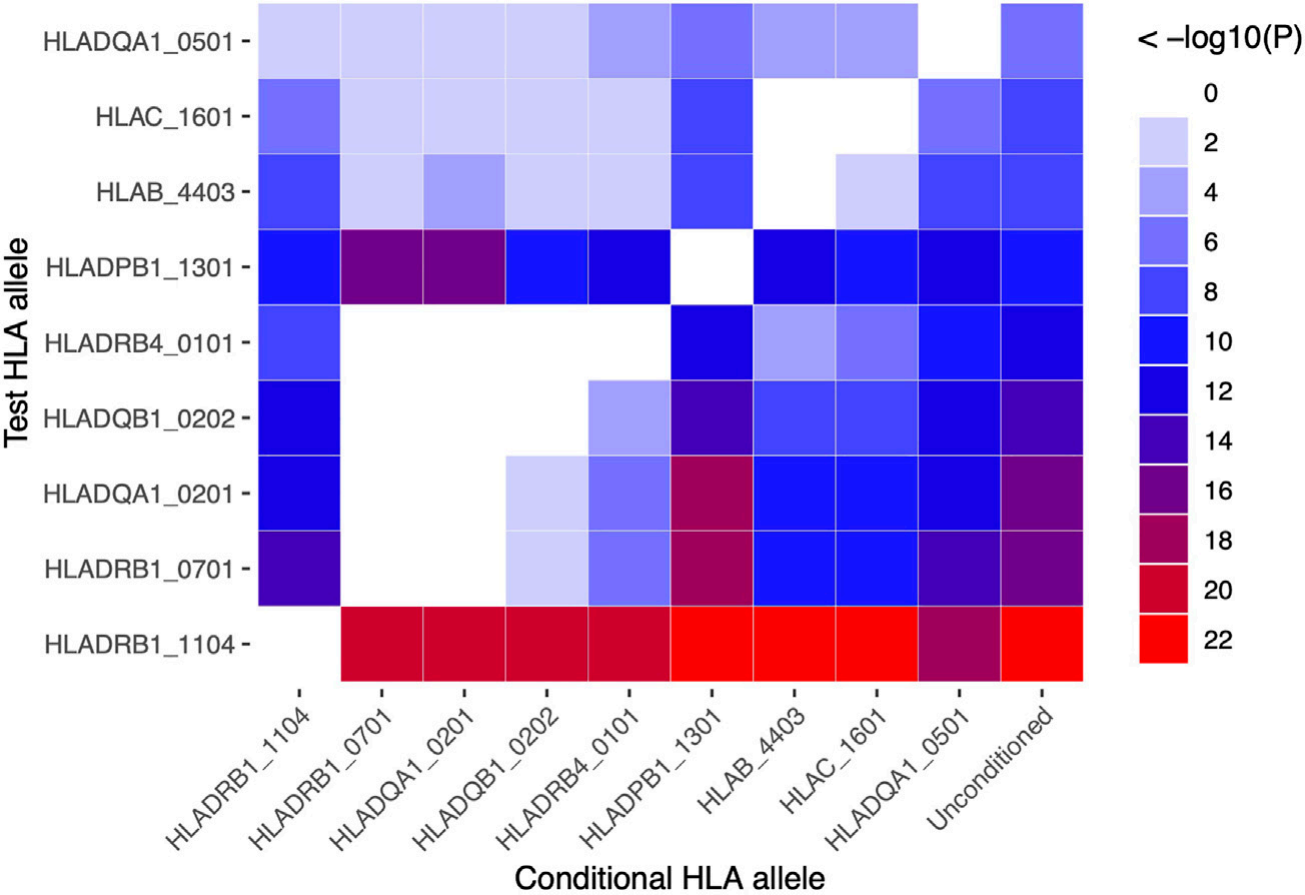
Pairwise conditional analysis of GWS and suggestive HLA class I and II associations in the SSc meta-analysis cohort. The *p*-value for the genetic association of each allele (y-axis) with disease upon correction for each allele (x-axis) in turn is denoted by the colour key on far right. Unconditioned *p*-values are shown in the right column for comparison.

HLA conditional analysis was performed on the meta-analysis cohort by sequential inclusion of the most significant SSc-associated HLA allele in the logistic regression model. Conditioning on the top disease-risk associated variant, HLA-DRB1*11:04, did not ablate significance of any of the remaining disease-associated class I or II alleles, apart from moderate reduction in significance of HLA-DQA1*05:01 (to *p* = 0.004). Additional correction for the second most strongly associated allele, HLA-DRB1*07:01, ablated the protective association with class II alleles HLA-DQA1*02:01, DQB1*02:02, and DRB4*01:01 (*p* >0.04), and, to a lesser extent, that of class I alleles HLA-B*44:03 (*p* = 2.0 × 10^−4^) and HLA-C*16:01 (*p* = 3.0 × 10^−4^), suggesting linkage of these loci within a common disease-protective haplotype. The residual disease-association signal seen for risk variant HLA-DRB1*13:01 remained significant (*p* = 1.2 × 10^−16^), and upon correction for this allele no residual HLA association was observed (Table 2). Pairwise conditional analysis further demonstrated that either HLA-DRB1*07:01 or DQA1*02:01 is the lead associated allele in the protective haplotype also containing DQB1*02:02 and DRB4*01:01, as conditioning on either entirely abolished the association signal with the remaining three alleles. Strong LD was also demonstrated between class I alleles HLA-B*44:03 and HLA-C*16:01; conditioning on the former abolished the *HLA-C* association (Figure 1).

**TABLE 2:** Conditional HLA association analysis in the meta-analysis cohort.

	DRB1*11:04		DRB1*11:04 + DRB1*07:01		DRB1*11:04 + DRB1*07:01 + DPB1*13:01	
HLA allele	OR	*p*	OR	*p*	OR	*p*
DRB1*11:04	—	—	—	—	—	—
DRB1*07:01	0.55	6.1x10^−15^	—	—	—	—
DQA1*02:01	0.56	1.4x10^−14^	2.70	0.3	2.85	0.2
DQB1*02:02	0.52	3.0x10^−13^	0.74	0.04	0.88	0.4
DRB4*01:01	0.57	1.0x10^−10^	0.80	0.05	0.84	0.1
DPB1*13:01	2.22	1.4x10^−12^	2.60	1.2x10^−16^	—	—
B*44:03	0.47	1.1x10^−9^	0.61	0.0002	0.63	0.0004
C*16:01	0.46	3.0x10^−8^	0.59	0.0003	0.61	0.0008
DQA1*05:01	1.18	3.8x10^−3^	1.09	0.1	1.09	0.1

*p*-values below the GWS threshold are underlined. Conditioning alleles are shown at top of table. CO = control, OR = odds ratio, p = *p*-value.

### HLA Associations With SSc Disease Subgroups

HLA associations were further assessed in subgroups of SSc patients differentiated by autoantibody subclass and limited or diffuse disease subtype (Table 3, Supplementary Tables S4–S9). *p*-values reported in the text below are derived from the meta-analysis cohort. The disease-risk association of HLA-DPB1*13:01 was strongest in the diffuse disease cohort (dcSSc OR = 3.2, *p* = 2.4 × 10^−13^; lcSSc OR = 1.75, *p* = 0.0001), whereas the protective associations of linked alleles HLA-DRB1*07:01 and DQA1*02:01, DQB1*02:02 and DRB4*01:01, as well as HLA-B*44:03 and HLA-C*16:01, were most pronounced in limited SSc (lcSSc OR<0.45, *p* <2.9 × 10^−8^; dcSSc OR>0.62, *p* >0.01). The disease risk association with HLA-DRB1*11:04 remained significant when assessed in both the limited (OR = 2.76, p_FDR_ = 6.5 × 10^−18^) and diffuse disease cohorts (OR = 2.93, p = 3.3 × 10^−12^). When allele frequencies were compared directly between lcSSc and dcSSc patients, the disease protective class II alleles HLA-DRB1*07:01, DQA1*02:01 and DQB1*02:02 were seen at lower frequency in lcSSc than dcSSc, albeit not at GWS with *p* <8.0 × 10^−5^ (Table 3). Extended tables of HLA allele associations calculated between disease subsets and controls, and between lcSSc and dcSSc, for all three cohorts are provided in Supplementary Tables S4–S6.

**TABLE 3:** HLA associations with SSc disease and major autoantibody subgroups.

	Metanalysis						Cohort 1						Cohort 2					
							ATA + vs −											
HLA allele	ATA− Prop.(Count)		ATA+ prop.(Count)		OR	*p*	ATA− Prop.(Count)		ATA+ prop.(Count)		OR	*p*	ATA− Prop.(Count)		ATA+ prop.(Count)		OR	*p*
DRB1*07:01	0.126	(123/974)	0.257	(3406/13273)	0.42	2.2 × 10^−18^	0.150	(53/353)	0.262	(1737/6632)	0.49	3.2 × 10^−6^	0.113	(70/621)	0.251	(1669/6641)	0.39	3.3 × 10^−13^
DQA1*02:01	0.127	(124/974)	0.256	(3397/13273)	0.43	5.3 × 10^−18^	0.153	(54/353)	0.261	(1733/6632)	0.51	6.1 × 10^−6^	0.113	(70/621)	0.251	(1664/6641)	0.39	3.9 × 10^−13^
DRB1*11:04	0.114	(111/974)	0.035	(459/13273)	2.76	6.5 × 10^−18^	0.076	(27/353)	0.035	(230/6632)	1.94	0.003	0.135	(84/621)	0.034	(229/6641)	3.26	1.6 × 10^−16^
DQB1*02:02	0.080	(78/974)	0.186	(2468/13273)	0.38	5.0 × 10^−16^	0.096	(34/353)	0.193	(1282/6632)	0.44	6.5 × 10^−6^	0.071	(44/621)	0.179	(1186/6641)	0.35	4.2 × 10^−11^
DRB4*01:01	0.094	(92/974)	0.188	(2491/13273)	0.45	1.2 × 10^−12^	0.113	(40/353)	0.194	(1289/6632)	0.52	0.0001	0.084	(52/621)	0.181	(1202/6641)	0.42	5.1 × 10^−9^
B*44:03	0.038	(37/974)	0.105	(1397/13273)	0.34	2.8 × 10^−10^	0.054	(19/353)	0.110	(728/6632)	0.46	0.001	0.029	(18/621)	0.101	(669/6641)	0.28	1.6 × 10^−7^
C*16:01	0.030	(29/974)	0.082	(1090/13273)	0.35	2.9 × 10^−8^	0.042	(15/353)	0.086	(573/6632)	0.46	0.004	0.023	(14/621)	0.078	(517/6641)	0.28	4.2 × 10^−6^
	**Diffuse SSc vs. Control**																	
	**HLA Allele**	**dcSSc Prop.(Count)**		**CO Prop.(Count)**		**OR**	**p**	**dcSSc Prop.(Count)**		**CO prop.(Count)**		**OR**	***p***	**dcSSc prop.(Count)**		**CO prop. Count)**		**OR**	***p***
DPB1*13:01	0.108	(51/474)	0.033	(444/13273)	3.20	2.4 × 10^−13^	0.109	(15/137)	0.035	(231/6632)	3.02	0.0001	0.107	(36/337)	0.032	(213/6641)	3.41	1.9 × 10^−10^
DRB1*11:04	0.131	(62/474)	0.035	(459/13273)	2.93	3.3 × 10^−12^	0.168	(23/137)	0.035	(230/6632)	3.47	1.9 × 10^−6^	0.116	(39/337)	0.034	(229/6641)	2.71	2.6 × 10^−7^
	**Diffuse SSc vs. Limited SSc**																	
	**HLA Allele**	**lcSSc Prop.(Count)**		**dcSSc prop.(Count)**		**OR**	***p***	**lcSSc prop.(Count)**		**dcSSc Prop.(Count)**		**OR**	**p**	**lcSSc prop.(Count)**		**dcSSc prop.(Count)**		**OR**	***p***
DQB1*02:02	0.08	(78/974)	0.146	(69/474)	2.04	6.2 × 10^−5^	0.096	(34/353)	0.124	(17/137)	1.39	0.31	0.071	(44/621)	0.154	(52/337)	2.54	2.4 × 10^−5^
DRB1*07:01	0.126	(123/974)	0.205	(97/474)	1.83	6.2 × 10^−5^	0.150	(53/353)	0.175	(24/137)	1.24	0.44	0.113	(70/621)	0.217	(73/337)	2.23	1.4 × 10^−5^
DQA1*02:01	0.127	(124/974)	0.205	(97/474)	1.81	8.0 × 10^−5^	0.153	(54/353)	0.175	(24/137)	1.22	0.47	0.113	(70/621)	0.217	(73/337)	2.23	1.4 × 10^−5^
	**ACA + vs. −**																	
	**Allele**	**ACA− prop.(Count)**		**ACA + prop.(Count)**		**OR**	***P***	**ACA− prop.(Count)**		**ACA + prop.(Count)**		**OR**	***P***	**ACA− prop.(Count)**		**ACA + prop.(Count)**		**OR**	***P***
DQA1*01:01	0.233	(210/903)	0.436	(221/507)	2.58	4.5 × 10^−15^	0.241	(64/266)	0.401	(87/217)	2.21	0.0001	0.229	(146/637)	0.462	(134/290)	2.99	1.2 × 10^−12^
DQB1*05:01	0.204	(184/903)	0.379	(192/507)	2.39	3.2 × 10^−12^	0.214	(57/266)	0.35	(76/217)	2.02	0.0009	0.199	(127/637)	0.400	(116/290)	2.76	1.8 × 10^−10^
DQA1*02:01	0.196	(177/903)	0.077	(39/507)	0.34	6.7 × 10^−9^	0.207	(55/266)	0.106	(23/217)	0.45	0.004	0.192	(122/637)	0.055	(16/290)	0.24	3.1 × 10^−7^
DRB1*07:01	0.195	(176/903)	0.077	(39/507)	0.34	9.3 × 10^−9^	0.203	(54/266)	0.106	(23/217)	0.47	0.006	0.192	(122/637)	0.055	(16/290)	0.24	3.1 × 10^−7^
DRB1*01:01	0.159	(144/903)	0.292	(148/507)	2.15	1.5 × 10^−8^	0.162	(43/266)	0.281	(61/217)	2.04	0.002	0.159	(101/637)	0.300	(87/290)	2.33	8.7 × 10^−7^
DQB1*02:02	0.134	(121/903)	0.043	(22/507)	0.29	2.8 × 10^−7^	0.147	(39/266)	0.055	(12/217)	0.35	0.003	0.129	(82/637)	0.034	(10/290)	0.23	2.1 × 10^−5^
	**ATA + vs. −**																	
	**Allele**	**ATA− prop.(Count)**		**ATA + prop.(Count)**		**OR**	***p***	**ATA− prop.(Count)**		**ATA + prop.(Count)**		**OR**	***p***	**ATA− prop.(Count)**		**ATA + prop.(Count)**		**OR**	***p***
DPB1*13:01	0.041	(48/1170)	0.250	(60/240)	7.85	9.3 × 10^−22^	0.035	(14/404)	0.228	(18/79)	8.54	1.2 × 10^−7^	0.044	(34/766)	0.261	(42/161)	8.39	6.8 × 10^−16^
DRB1*11:04	0.083	(97/1170)	0.300	(72/240)	4.13	9.7 × 10^−14^	0.069	(28/404)	0.266	(21/79)	4.93	9.1 × 10^−6^	0.09	(69/766)	0.317	(51/161)	3.9	3.3 × 10^−9^
DQA1*01:01	0.339	(397/1170)	0.133	(32/240)	0.30	2.4 × 10^−9^	0.347	(140/404)	0.114	(9/79)	0.23	0.0001	0.336	(257/766)	0.143	(23/161)	0.33	4.2 × 10^−6^
DPA1*02:01	0.271	(317/1170)	0.454	(109/240)	2.33	1.0 × 10^−8^	0.260	(105/404)	0.519	(41/79)	3.26	5.9 × 10^−6^	0.277	(212/766)	0.422	(68/161)	2.17	2.8 × 10^−5^
DRB1*15:01	0.203	(237/1170)	0.350	(84/240)	2.40	4.3 × 10^−8^	0.223	(90/404)	0.367	(29/79)	2.25	0.003	0.192	(147/766)	0.342	(55/161)	2.37	1.6 × 10^−5^
DRB5*01:01	0.205	(240/1170)	0.354	(85/240)	2.38	5.0 × 10^−8^	0.223	(90/404)	0.367	(29/79)	2.25	0.003	0.196	(150/766)	0.348	(56/161)	2.33	1.8 × 10^−5^
DQB1*06:02	0.197	(231/1170)	0.329	(79/240)	2.33	2.2 × 10^−7^	0.210	(85/404)	0.367	(29/79)	2.55	0.0008	0.191	(146/766)	0.311	(50/161)	2.11	0.0002
	**ANoA + vs. −**																	
	**Allele**	**ANoA− prop.(Count)**		**ANoA + prop.(Count)**		**OR**	***p***	**ANoA− prop.(Count)**		**ANoA + prop.(Count)**		**OR**	***p***	**ANoA− prop.(Count)**		**ANoA + prop.(Count)**		**OR**	***p***
DQA1*01:01	0.340	(359/1055)	0.201	(71/353)	0.48	9.2 × 10^−7^	0.335	(119/355)	0.244	(31/127)	0.62	0.04	0.343	(240/700)	0.177	(40/226)	0.41	4.6 × 10^−6^
DQA1*05:01	0.474	(500/1055)	0.626	(221/353)	1.84	1.6 × 10^−6^	0.473	(168/355)	0.622	(79/127)	1.84	0.005	0.474	(332/700)	0.628	(142/226)	1.83	0.0001
DQB1*02:01	0.227	(240/1055)	0.337	(119/353)	1.88	4.7 × 10^−6^	0.262	(93/355)	0.339	(43/127)	1.47	0.09	0.21	(147/700)	0.336	(76/226)	2.21	6.4 × 10^−6^
C*07:01	0.304	(321/1055)	0.433	(153/353)	1.78	5.8 × 10^−6^	0.332	(118/355)	0.457	(58/127)	1.69	0.01	0.29	(203/700)	0.42	(95/226)	1.86	0.0001

CO = control, OR = odds ratio, p = *p*-value, Prop. = proportion.

Considering associations with autoantibodies, HLA-DQA1*01:01, DQB1*05:01 and DRB1*01:01 were seen at significantly increased frequency in ACA + compared with ACA − SSc (HLA-DQA1*01:01 OR = 2.58, *p* = 4.5 × 10^−15^; HLA-DQB1*05:01 OR = 2.39, *p* = 3.2 × 10^−12^; HLA-DRB1*01:01 OR = 2.15, *p* = 1.5 × 10^−8^ in the meta-analysis cohort), and reduced frequency in ATA + relative to ATA − disease (HLA-DQA1*01:01 OR = 0.3, p_FDR_ = 2.4 × 10^−9^; HLA-DQB1*05:01 OR = 0.38, p_FDR_ = 1.7 × 10^−6^; HLA-DRB1*01:01 OR = 0.42, *p* = 0.0001; Table 3). The HLA-DQA1*01:01 and DQB1*05:01 alleles were seen at 43.6 and 37.9% respectively in ACA + SSc relative to only 13.3% (both) in ATA + disease and 27 and 23% respectively in controls. HLA-DQA1*01:01 was also the top HLA to show a suggestive association with ANoA + disease, where it was carried at a reduced frequency relative to ANoA − disease (OR = 0.48, *p* = 9.2 × 10^−7^). Alternatively, suggestive disease risk associations were seen with HLA-DQA1*05:01 and DQB1*02:01 in the ANoA + cohort (HLA-DQA1*05:01 OR = 1.84, *p* = 1.6 × 10^−6^; HLA-DQB1*02:01 OR = 1.88, *p* = 4.7 × 10^−6^), and the class 1 allele HLA-C*07:01 was seen at increased frequency relative to ANoA − disease, albeit not at GWS (OR = 1.78, *p* = 5.8 × 10^−6^; Table 3).

The independent SSc disease-risk association with alleles HLA-DPB1*13:01 and HLA-DRB1*11:04 appeared to be driven almost exclusively by ATA + patients, in which these alleles occurred at significantly higher frequency than in ATA − disease (HLA-DPB1*13:01 OR = 7.85, p_FDR_ = 9.3 × 10^−22^; HLA-DRB1*11:04 OR = 4.13, *p* = 9.7 × 10^−14^), increasing odds of ATA + SSc disease specifically substantially above that of SSc alone (Table 3). HLA-DPB1*13:01 was seen in 25 and 4%, and HLA-DRB1*11:04 in 30 and 8% of patients positive and negative for this autoantibody respectively. A significant increase in the frequency of alleles HLA-DPA1*02:01 (OR = 2.33, *p* = 1.0 × 10^−8^), DRB1*15:01 (OR = 2.40, *p* = 4.3 × 10^−8^) and DRB5*01:01 (OR = 2.38, *p* = 5.0 × 10^−8^) was also seen in ATA + relative to ATA − SSc, though these alleles did not show a significant association with risk of SSc, or either disease subtype, overall. The strongest HLA class I associations were seen with lcSSc rather than dcSSc (HLA-B*44:03 OR = 0.34, *p* = 2.8 × 10^−10^; HLA-C*16:01 OR = 0.35, *p* = 2.9 × 10^−8^ lcSSc versus control; Table 3). Extended tables of HLA allele frequencies in each antibody positive subgroup of disease are provided in Supplementary Tables S7–S9.

### KIR-HLA Class I Interactions in SSc

Given that certain HLA class I alleles serve as ligands for inhibitory and activating KIRs, an interaction evolved to buffer the innate killing activity of NK cells and other lymphocyte populations, HLA class I subgroup and allele associations with SSc were assessed in the context of genetic epistasis with their canonical KIRs. *KIR* imputation approaches allow profiling and statistical analysis of the highly polymorphic *KIR* locus in sizeable cohorts unamenable to lab-based copy-number typing. Imputed gene and haplotype frequencies across both study cohorts were in close agreement with data from publicly available and published European cohorts (Supplementary Figures S3,S4) and utilised for statistical comparison between SSc cases and controls. Before accounting for HLA background, none of the 14 KIR loci showed a significant association with SSc under a dominant or recessive inheritance model. SSc associated allele HLA-B*44:03, protective for lcSSc, is a member of the HLA-Bw4 subclass of KIR ligands recognised by inhibitory receptor KIR3DL1. The linked-allele HLA-C*16:01 is a HLA-C1 ligand carrying a residue 80 asparagine recognised by inhibitory receptors KIR2DL2 and KIR2DL3. An epistatic interaction was detected between KIR3DL1 and HLA-Bw4 in the SSc meta-analysis cohort, such that fewer KIR3DL1+ SSc patients carried an appropriate HLA-Bw4 ligand for this inhibitory receptor (67.7%) relative to KIR3DL1+ controls (72.2%, p_int_ = 0.02); accordingly, the protective association of HLA-Bw4 alleles with SSc was seen only in KIR3DL1+ individuals (OR = 0.77, *p* = 2.0 × 10^−5^; Table 4). No significant difference was observed in HLA-B*44 carriage in KIR3DL1+ compared with KIR3DL1-carriers, although the very small sample sizes preclude reliable interpretation. An interaction was also observed between HLA-C1 ligands and KIR2DL3/L2/S2 genes (all of which show perfect LD), such that HLA-C1 individuals carrying the activating receptor KIR2DS2 (and thus lacking paired inhibitory receptor KIR2DL3 in inverse LD) were at an increased risk of disease relative to KIR2DL3+ individuals (p_int_ = 0.03). The inhibitory receptor KIR2DL3 was seen at higher frequency in HLA-C*16 + controls (90.2%) than HLA-C*16 + SSc, cases (79.7%; OR = 0.44, *p* = 0.009), though did not differ in frequency in HLA-C*16- case-control analysis (p_int_ = 0.02; Table 4), suggesting that the protective association of this class I allele may be mediated through inhibitory KIR, interactions. Extended tables showing statistical interactions between biologically validated KIR-HLA, pairs are provided in Supplementary Tables S10–S11.

**TABLE 4:** KIR interactions with HLA class I subtypes and alleles.

	—	Metanalysis							Cohort 1							Cohort 2						
HLA	KIR	SSc prop.(Count)		CO prop.(Count)		OR	*p*	p_int_	SSc prop.(Count)		CO prop.(Count)		OR	*p*	p_int_	SSc Prop.(Count)		CO prop.(Count)		OR	*p*	p_int_
*Bw4*	*3DL1+*	0.677	(947/1399)	0.722	(9121/12639)	0.77	2x10^−5^	0.02	0.683	(332/486)	0.72	(4542/6305)	0.81	0.03	0.06	0.674	(615/913)	0.723	(4579/6334)	0.75	0.0002	0.15
	*3DL1-*	0.833	(55/66)	0.729	(462/634)	1.62	0.17		0.941	(16/17)	0.722	(236/327)	5.21	0.12		0.796	(39/49)	0.736	(226/307)	1.27	0.54	
*C1*	*2DL3+*	0.861	(1139/1323)	0.871	(10537/12097)	0.99	0.88	0.03	0.889	(399/449)	0.87	(5270/6058)	1.29	0.10	0.60	0.847	(740/874)	0.872	(5267/6039)	0.86	0.15	0.02
	*2DL3-/L2++/S2++*	0.93	(132/142)	0.867	(1020/1176)	2.14	0.03		0.926	(50/54)	0.88	(505/574)	1.96	0.22		0.932	(82/88)	0.855	(515/602)	2.63	0.03	

		**Metanalysis**							**Cohort 1**							**Cohort 2**						
**KIR**	**HLA Allele**	**SSc** **prop.(Count)**		**CO prop.(Count)**		**OR**	***p***	**p**_**pnt**_	**SSc** **prop.(Count)**		**CO prop.(Count)**		**OR**	***p***	**p**_**int**_	**SSc** **prop.(Count)**		**CO prop.(Count)**		**OR**	***p***	**p**_**int**_
*2DL3*	C*16+ (C1/C2)	0.797	(59/74)	0.902	(1044/1157)	0.44	0.009	0.02	0.828	(24/29)	0.902	(543/602)	0.49	0.18	0.43	0.778	(35/45)	0.903	(501/555)	0.38	0.02	0.01
	C*16- (C1/C2)	0.909	(1264/1391)	0.912	(11053/12116)	1.01	0.93		0.897	(425/474)	0.915	(5515/6030)	0.85	0.30		0.915	(839/917)	0.910	(5538/6086)	1.12	0.37	

Statistical interactions between biologically interacting KIR and HLA subgroups or alleles reaching statistical significance (*p* < 0.05) in the meta-analysis cohort. + = positive, − = negative, ++ = homozygous, OR = odds ratio, CO = control, *p* = p-value, pint = KIRxHLA interaction term p-value.

## Discussion

Both protective and disease-risk associations with classical HLA alleles are among the strongest seen in human immune-mediated disease, attesting to the central role of these molecules in dictating productive and pathologic immune responses. Additional to their function in guiding T cell specificity through presentation of self and foreign derived antigens, engagement of HLA ligands in a less antigen-dependent manner by inhibitory and activating lymphocyte receptors make them key moderators of lymphocyte activity. Systemic sclerosis remains an immunological perplexity; strong HLA associations, alongside emergence of serum autoantibodies that segregate with clinical phenotype, suggest an autoimmune axis. However, whether autoantibodies are drivers of cascading inflammation, fibrosis, organ destruction and resulting mortality in this disease remains unknown. Here we report on HLA associations detected in two independent and meta-analysed cohorts of SSc patients, and present findings from direct case-case comparisons of HLA frequencies in autoantibody positive and negative subgroups of disease. Furthermore, we identify statistical interactions between KIRs and HLA class I ligands in the largest analysis of genetic epistasis between these loci in SSc.

Meta-analysis of HLA frequencies in SSc patients and healthy controls revealed strong risk associations with class II alleles HLA-DRB1*11:04 and HLA-DPB1*13:01, confirmed by step-wise and pair-wise conditional analysis to represent two independent disease-associated loci. HLA-DRB1*11:04 associations were seen with both lcSSc and dcSSc, but the HLA-DPB1*13:01 association reached GWS in the dcSSc cohort alone. The frequencies of both HLA-DPB1*13:01 and HLA-DRB1*11:04 alleles were significantly increased in ATA + relative to ATA −disease (OR = 7.85 and OR = 4.13 respectively), however neither differed in frequency from controls in ATA − or ACA + SSc cohorts, indicating the strong specificity of these genetic association for patients with ATA + serology. Both HLA-DRB1*11:04 and HLA-DPB1*13:01 alleles have been reported across a diversity of ethnic groups to be associated with SSc risk, and, specifically, ATA + disease (Zochling et al., 2014; Gourh et al., 2020; Acosta-Herrera et al., 2021). Furthermore, HLA-DRB1*11 positivity, alongside ATAs and/or dcSSc, has been reported as the strongest risk factor for SSc associated pulmonary fibrosis in a United Kingdom cohort (Fanning et al., 1998). In a recent functional investigation of the link between topoisomerase-I (TOP1) derived peptides and *HLA-DR* alleles in 6 ATA + SSc patients, a restricted and shared set of TOP1 peptides were eluted off HLA-DR molecules from patient monocyte-derived dendritic cells pulsed with recombinant human TOP1. Sequence analysis of ATA + SSc associated *HLA-DR* alleles (HLA-DRB1*11:01, *11:04, *08:02, *08:04, and*15:02), which were inherited by 3 of the 6 patients, revealed two strongly conserved motifs in the peptide-contact region of the DRβ-chain. Two further individuals carried HLA-DRB1*15:01 and *16:01, alleles with no previously reported ATA + SSc association, but which contained identical peptide-contact motifs to ATA−associated alleles, and shared TOP1 epitopes isolated from all such alleles elicited CD4^+^ T cell activation in 73% of ATA + SSc patients relative to 27% of ATA−patients (Tiniakou et al., 2020). Relevant to this, this study is the first to show a GWS association of HLA-DRB1*15:01 with ATA + relative to ATA − SSc (OR = 2.4, *p* = 4.3 × 10^−8^), following the recently published association of this allele with ATA + SSc by case-control analysis (Acosta-Herrera et al., 2021), suggesting commonalities in the preferential binding of this and other ATA + SSc associated HLA-DRB1 alleles to TOP1 derived peptides. These findings support the hypothesis that exposure to an environmentally derived TOP1 peptide mimic, presented by risk class II alleles, triggers development of ATA + antibodies, and suggest that autoantibodies may play a direct role in SSc-associated lung disease.

Alleles HLA-DRB1*07:01, DQA1*02:01, DQB1*02:02, and DRB4*01:01 demonstrated a protective association with SSc. Pairwise conditioning on either allele HLA-DRB1*07:01 or DQA1*02:01 was sufficient to abolish disease association at the remaining three loci, confirming tight linkage of these two lead disease-protective alleles on a common haplotype. This is in contrast to previous conditional analyses which have reported HLA-DQB1*02:02 as carrying the strongest independent protective association with disease (Gourh et al., 2020; Acosta-Herrera et al., 2021). Here, the DRB1*07:01-DQA1*02:01 haplotype was associated with lcSSc alone, alongside HLA-DQB1*02:02, showing a reduced frequency in lcSSc when compared to dcSSc directly (*p* <8 × 10^−5^). The haplotype showed a GWS association with ACA + but not ATA + disease, as previously reported (Gourh et al., 2020; Acosta-Herrera et al., 2021).

To our knowledge, no studies have yet reported GWS HLA associations between auto-antibody positive and negative subgroups of SSc patients. In addition to associations with ATA + SSc discussed above, significantly increased frequencies of the class II alleles HLA-DQA1*01:01, DQB1*05:01, and DRB1*01:01 were seen with ACA + relative to ACA − disease, confirming published findings from smaller studies comparing patient subgroups across multiple ethnicities (Kuwana et al., 1995; Fanning et al., 1998; Simeon et al., 2009). Recent mapping of antigen targets for serum autoantibodies taken from patients with SSc, Sjögren’s syndrome and primary biliary cholangitis showed broad spectrum reactivity of ACAs against multiple centromere antigens in a subset of patients, suggesting recognition of the tertiary structure of the centromere-kinetochore macrocomplex rather than single protein derived epitopes (Kajio et al., 2021). Such complexity in the epitope mapping of ACAs is likely to also complicate derivation of linear peptides that may link ACA−associated class II alleles to autoantibody production, with binding of centromere derived peptides to genetically implicated HLAs yet to be shown. Alternate strategies such as investigating peptides binding and/or eluted from disease associated compared with protective alleles offers a different and potentially more productive approach.

We are the first to report GWS protective HLA class I association with alleles HLA-B*44:03 and HLA-C*16:01 in SSc, detected in both the total SSc cohort meta-analysis and, specifically, with lcSSc. Consistent with our findings, a suggestive HLA-B*44:03 association with the same direction of effect has been reported previously in European Americans; however this was lost upon conditioning with associated alleles HLA-DQB1*02:02, DPB1*13:01, and DRB1*11:04 (Gourh et al., 2020). Here, pairwise and iterative conditional analysis demonstrated strong LD between HLA-B*44:03 and HLA-C*16:01, and weaker but detectable LD with the protective class II haplotype containing HLA-DRB1*07:01. Although associations with both HLA class I alleles were weakened upon correction for all three independently associated class II alleles, HLA-DRB1*11:04, DRB1*07:01 and DPB1*13:01, the genetic signal remained significant (*p* <0.0008), suggesting that the observed HLA class I associations may be more than accessory to co-inherited class II alleles. Of relevance, an epistatic interaction was observed between HLA-C*16 and the KIR locus, suggesting this allele may play an independent yet complementary role in disease protection. Of HLA-C*16 + individuals, 79.7% of SSc patients co-inherited *KIR2DL3* relative to 90.2% of controls (OR = 0.44, *p* = 0.009), whereas *KIR2DL3* frequencies did not differ in HLA-C*16- individuals. *KIR2DL3* encodes an inhibitory receptor for HLA-C1 subgroup ligands*.* In a functional investigation of KIR2DL3 binding to a panel of 97 bead-bound HLA class I alleles expressing a broad repertoire of peptides, HLA-C*16:01 (a C1 ligand) ranked 8^th^ in its binding avidity for KIR2DL3 and was one of the strongest inhibitors of KIR2DL3+ NK cell degranulation (Moradi et al., 2021). In our analyses, KIR2DL3 was conversely associated with increased disease risk when inherited with HLA-C2 allele HLA-C*04, which binds the KIR2DL3 receptor with only 10% of the avidity seen for its strongest HLA-C1 binding partner (Moradi et al., 2021). These findings emphasise the importance of functional context when interpreting *KIR-HLA* epistatic interactions in disease, and suggest that co-inheritance of HLA-C*16 and *KIR2DL3* may protect from SSc through increasing the activation threshold of KIR2DL3 expressing lymphocytes.

The potential contribution of KIR-HLA ligand interactions to the risk and/or severity of immune-mediated disease has been explored across many conditions. In our analyses, co-inheritance of *HLA-Bw4* ligands in the presence of their compatible inhibitory receptor *KIR3DL1* was significantly associated with disease protection in both independent disease cohorts (meta-analysis *p* = 2 × 10^−5^), and inheritance of HLA-C1 alleles in the absence of their inhibitory receptor *KIR2DL3* was significantly associated with disease risk, albeit with more modest strength of association (*p* = 0.03). This suggests that signalling through inhibitory KIRs may reduce the risk of chronic, damaging, immune activation and protect against SSc. Among other diseases with immune involvement, co-inheritance of *KIR3DL1* and *HLA-Bw4*, or specific *HLA-Bw4* alleles, has been associated with protection from multiple sclerosis (Hollenbach et al., 2016) and ankylosing spondylitis (Hanson et al., 2020), and both *KIR3DL1-HLA-Bw4* and *KIR2DL3-HLA-C1* co-inheritance with protection from autoimmune hepatitis (Littera et al., 2016). An absence of inherited strong inhibitory KIR-HLA interacting partners may be hypothesised to predispose to a lower threshold of lymphocyte activation and increased risk of autoinflammatory disease. A study of NK cell frequency and function in SSc patients’ blood demonstrated a significant increase in NK cell numbers in dcSSc relative to controls, and increased expression of cell surface activation markers CD16 and CD69 on both lcSSc and dcSSc NK populations (Horikawa et al., 2005). However, upon *in vitro* stimulation, SSc patient NK cells also exhibited a significant reduction in interferon γ (IFNγ) production, a reduced ability to lyse target cells, and reduced granzyme B secretion. Conflicting studies surround the functional role of NK cells in SSc, emphasising that the contribution of this lymphocyte subset to disease is poorly understood. Our analyses suggest that variable inheritance of NK inhibitory and activating receptors imparts an additional layer of genetic heterogeneity to immune cell involvement in this condition.

Here we report an extensive analysis of class I and II HLA associations with SSc, and clinical and serological subtypes of disease, in two independent and meta-analysed cohorts of patients and controls. The substantial size of our study cohort has enabled us to identify HLA associations at GWS that differentiate autoantibody positive and negative SSc patients, emphasising the genetic heterogeneity underpinning this disease. Furthermore, we identify two new HLA class 1 associations, and show that co-inheritance of HLA class I ligands and KIRs, receptors that serve as key modulators of lymphocyte activation, may further contribute to an individual’s underlying risk of developing SSc. Clear elucidation of genetic associations with disease risk and autoantibody positivity in SSc may aid in functional studies addressing the inflammatory triggers for disease, in the form of both endogenous host antigens and environmental stimuli that disrupt immune tolerance.

## Data Availability

Extended summary statistics for HLA and KIR association analyses are provided in the Supplementary Material. Access requests for participant genotypes should be made through the corresponding author MB matt.brown@kcl.ac.uk.
