# Supplementary material for: Contribution of HLA and KIR Alleles to Systemic Sclerosis Susceptibility and Immunological and Clinical Disease Subtypes
Source: Front Genet. 2022 Jun 8;13:913196. doi: 10.3389/fgene.2022.913196 (PMC9214260; doi:10.3389/fgene.2022.913196)
Supplement: Supplementary file 1 [file DataSheet2.PDF]

Supplementary Table 1: Previously reported HLA associations with SSc, and clinical and serological SSc subtypes, from large-scale cohort studies.

Study	Ethnicity	Sample Size	Comparison	Allele	OR	p
Acosta-Herrera M. (2021) *Independent associations reported with conditioning on top allele*	Caucasian	9,095 SSc 17,584 CO	SSc vs CO	HLA-DRB1*11:04	2.1	$2.5x10^{-56}$
				HLA-DRB1*13:01	0.7	$3.0x10^{-14}$
				HLA-DQB1*02:02	0.6	$3.8x10^{-45}$
				HLA-DQB1*05:01	1.2	$1.3x10^{-12}$
				HLA-DPB1*13:01	2.1	$9.8x10^{-30}$
				HLA-DPB1*03:01	1.2	$4.8x10^{-8}$
				HLA-DPB1*06:01	1.5	$2.1x10^{-8}$
				HLA-DQA1*04:01	1.9	$3.0x10^{-28}$
				HLA-B*08:01	1.2	$1.8x10^{-12}$
		5,686 lcSSc 17,584 CO	lcSSc vs CO	HLA-DQA1*02:01	0.5	$5.2x10^{-51}$
				HLA-DRB1*08:01	2.2	$8.1x10^{-29}$
				HLA-DRB1*11:04	1.8	$5.0x10^{-24}$
				HLA-DRB1*13:01	0.7	$1.9x10^{-9}$
				HLA-DQB1*05:01	1.4	$7.3x10^{-21}$
				HLA-DPB1*13:01	1.7	$2.2x10^{-17}$
		2,524 dcSSc 17,584 CO	dcSSc vs CO	HLA-DRB1*11:04	3.2	$3.3x10^{-75}$
				HLA-DPB1*13:01	3.1	$3.8x10^{-41}$
				HLA-DQA1*05:01	1.5	$1.6x10^{-11}$
		2,524 dcSSc 5,686 lcSSc	dcSSc vs lcSSc	HLA-DQA1*02:01	0.7 (vs dcSSc)	$2.1x10^{-8}$
				HLA-DQA1*05:01	1.3 (vs lcSSc)	$1.8x10^{-11}$
		1,749 ATA+ SSc 17,584 CO	ATA+ vs CO	HLA-DPB1*13:01	6.8	$2.2x10^{-138}$
				HLA-DRB1*11:04	4.9	$4.6x10^{-127}$
				HLA-DRB1*15:01	1.5	$3.4x10^{-22}$
				HLA-DPA1*02:01	1.9	$2.9x10^{-19}$
				HLA-DQB1*03:01	1.8	$7.0x10^{-19}$
				HLA-DQB1*03:03	1.7	$4.9x10^{-9}$
		3,344 ACA+ SSc 17,584 CO	ACA+ vs CO	HLA-DQB1*05:01	2.0	$1.2x10^{-66}$
				HLA-DRB1*08:01	3.2	$4.0x10^{-64}$
				HLA-DRB1*07:01	0.4	$1.8x10^{-45}$
				HLA-DQA1*03:01	1.3	$2.0x10^{-20}$
		1,749 ATA+ 3,344 ACA+	ATA+ vs ACA+	HLA-DRB1*08:01	2.2 (vs ATA+)	$1.4x10^{-10}$
				HLA-DRB1*07:01	0.4 (vs ATA+)	$3.9x10^{-27}$
				HLA-DPA1*02:01	2.1 (vs ACA+)	$1.1x10^{-40}$
				HLA-DQB1*03:01	1.7 (vs ACA+)	$1.7x10^{-22}$
		558 ARA+ 17,584 CO	ARA+ vs CO	HLA-DRB1*11:04	2.6	$1.7x10^{-16}$
Gourh P. (2020) *Independent associations reported with conditioning on top allele*	African American	662 SSc 946 CO	SSc vs CO	HLA-DRB1*08:04	3.2	$3.2x10^{-16}$
				HLA-DRB1*11:02	2.3	$1.8x10^{-6}$
		129 AFA+ SSc 946 CO	AFA+ vs CO	HLA-DRB1*08:04	7.4	$2.6x10^{-19}$
				HLA-DQB1*06:09	4.1	$2.0x10^{-6}$
		182 ATA+ SSc 946 CO	ATA+ vs CO	HLA-DPB1*13:01	4.3	$2.4x10^{-12}$
				HLA-DQB1*02:01	0.3	$2.1x10^{-7}$
				HLA-DQA1*05:01	2.1	$2.2x10^{-5}$
	European American	723 SSc 5,347 CO	SSc vs CO	HLA-DQB1*02:02	0.5	$3.6x10^{-9}$
				HLA-DPB1*13:01	2.6	$1.0x10^{-8}$
				HLA-DRB1*11:04	2	$1.0x10^{-8}$
		115 ATA+ SSc 5,347 CO	ATA+ vs CO	HLA-DPB1*13:01	13.7	$1.5x10^{-24}$
				HLA-DRB1*11:04	6.5	$1.6x10^{-11}$
		238 ACA+ SSc 5,347 CO	ACA+ vs CO	HLA-DRB1*07:01	0.1	$4.8x10^{-20}$
				HLA-DQB1*05:01	2.0	$2.9x10^{-6}$
				HLA-DQA1*04:01	2.7	$6.7x10^{-6}$
Zochling J. (2014)	Caucasian	486 SSc 4,458 CO	SSc vs CO	HLA-DRB1*11:04	3.1	$2.8x10^{-7}$
				HLA-DRB1*07:01	0.6	$1.4x10^{-5}$
		44% vs 56% SSc	ACA+ vs ACA-	HLA-DRB1*07:01	Prot. for ACA+	0.002
		16% vs 84% SSc	ATA+ vs ATA-	HLA-DRB1*11:04	Risk for ATA+	0.02
				HLA-DRB1*07:01	Prot. for ATA-	0.001

Supplementary Table 2: Known subclass classifications of Class I HLA alleles

HLA subgroup	Alleles
HLA-C1	HLA-C*01, C*03, C*07, C*08, C*12, C*13, C*14, C*16 and HLA-B*46:01, B*73:01 (Asparagine at position 80 in the alpha-helix; HLA-C Asn80)
HLA-C2	HLA-C*02, C*04, C*05, C*06, C*15, C*17, C*16:02, C*18 (Lysine at position 80 in the alpha-helix; HLA-C Lys80)
HLA-Bw4I80	HLA-B*15:13, 15:16, B*15:17, B*15:23, B*15:24, B*27:02, B*38:01, B*49, B*51, B*52, B*53, B*57, B*58, B*59, HLA-A*23, A*24, A*25, A*32 (Bw4 defined by an epitope at position 77-83 in the alpha1-helix; isoleucine at position 80)
HLA-Bw4T80	HLA-B*13, B*27:01, B*27:03, B*27:04, B*27:05, B*27:07, B*37, B*38:02, B*44, B*47 (Bw4 is defined by an epitope at position 77-83 in the alpha1-helix; threonine at position 80)
HLA-Bw6	HLA-B*07, B*08, B*14, B*15:01, B*15:02, B*15:03, B*15:05, B*15:08, B*15:09, B*15:10, B*15:14, B*15:15, B*15:18, B*15:25, B*15:29, B*18, B*22, B*27:08, B*35, B*39, B*40, B*41, B*42, B*45, B*46, B*48, B*50, B*54, B*55, B*56, B*60, B*61, B*62, B*64, B*65, B*67, B*70, B*71, B*72, B*73, B*75, B*76, B*78, B*81, B*82.
	N.B. In some cases HLA alleles differ in their Bw4I80/T80/Bw6 classification at four-digit resolution.

Table adapted from (Hanson et al., 2020)

Supplementary Table 3: Classical HLA allele frequencies and disease associations with SSc in all three study cohorts.

	SSC vs CONTROL											
	Meta-analysis Cohort				Cohort 1				Cohort 2			
HLA Allele	SSc Prop.(Count)	CO Prop.(Count)	OR	p	SSc Prop.(Count)	CO Prop.(Count)	OR	p	SSc Prop.(Count)	CO Prop.(Count)	OR	p
HLA-DRB1*1104	0.119 (174/1465)	0.035 (459/13273)	2.81	2.30E-25	0.099 (50/503)	0.035 (230/6632)	2.36	6.40E-07	0.129 (124/962)	0.034 (229/6641)	3.08	1.30E-19
HLA-DRB1*0701	0.152 (223/1465)	0.257 (3406/13273)	0.52	1.50E-17	0.157 (79/503)	0.262 (1737/6632)	0.52	2.50E-07	0.15 (144/962)	0.251 (1669/6641)	0.53	4.40E-11
HLA-DQA1*0201	0.153 (224/1465)	0.256 (3397/13273)	0.53	3.50E-17	0.159 (80/503)	0.261 (1733/6632)	0.53	4.80E-07	0.15 (144/962)	0.251 (1664/6641)	0.53	5.40E-11
HLA-DQB1*0202	0.102 (149/1465)	0.186 (2468/13273)	0.49	1.70E-15	0.103 (52/503)	0.193 (1282/6632)	0.47	6.00E-07	0.101 (97/962)	0.179 (1186/6641)	0.51	2.40E-09
HLA-DRB4*0101	0.111 (162/1465)	0.188 (2491/13273)	0.54	9.30E-13	0.109 (55/503)	0.194 (1289/6632)	0.5	2.60E-06	0.111 (107/962)	0.181 (1202/6641)	0.57	2.90E-07
HLA-DPB1*1301	0.076 (111/1465)	0.033 (444/13273)	2.2	1.80E-12	0.068 (34/503)	0.035 (231/6632)	1.85	1.40E-03	0.08 (77/962)	0.032 (213/6641)	2.5	7.50E-11
HLA-B*4403	0.05 (73/1465)	0.105 (1397/13273)	0.45	1.80E-10	0.066 (33/503)	0.11 (728/6632)	0.57	2.20E-03	0.042 (40/962)	0.101 (669/6641)	0.4	4.90E-08
HLA-C*1601	0.038 (55/1465)	0.082 (1090/13273)	0.44	6.20E-09	0.05 (25/503)	0.086 (573/6632)	0.55	4.00E-03	0.031 (30/962)	0.078 (517/6641)	0.39	9.10E-07
HLA-DQA1*0501	0.508 (744/1465)	0.421 (5583/13273)	1.34	1.70E-07	0.509 (256/503)	0.414 (2747/6632)	1.4	2.80E-04	0.507 (488/962)	0.427 (2836/6641)	1.29	3.00E-04
HLA-DQA1*0401	0.073 (107/1465)	0.047 (618/13273)	1.74	4.00E-07	0.078 (39/503)	0.045 (298/6632)	1.92	2.70E-04	0.071 (68/962)	0.048 (320/6641)	1.61	7.20E-04
HLA-DRB4*9901	0.93 (1363/1465)	0.883 (11721/13273)	1.72	4.30E-07	0.924 (465/503)	0.876 (5811/6632)	1.68	2.80E-03	0.933 (898/962)	0.89 (5910/6641)	1.68	1.50E-04
HLA-A*2902	0.044 (65/1465)	0.079 (1046/13273)	0.53	1.10E-06	0.048 (24/503)	0.083 (552/6632)	0.53	2.80E-03	0.043 (41/962)	0.074 (494/6641)	0.54	2.30E-04
HLA-DQA1*0103	0.083 (122/1465)	0.12 (1588/13273)	0.62	1.30E-06	0.074 (37/503)	0.119 (787/6632)	0.56	9.90E-04	0.088 (85/962)	0.121 (801/6641)	0.64	3.00E-04
HLA-DQB1*0603	0.076 (112/1465)	0.114 (1515/13273)	0.61	2.30E-06	0.064 (32/503)	0.113 (752/6632)	0.52	4.40E-04	0.083 (80/962)	0.115 (763/6641)	0.66	9.40E-04
HLA-DQB1*0402	0.074 (109/1465)	0.049 (645/13273)	1.66	2.80E-06	0.08 (40/503)	0.048 (316/6632)	1.82	7.00E-04	0.072 (69/962)	0.05 (329/6641)	1.55	1.60E-03
HLA-B*5601	0.023 (33/1465)	0.009 (113/13273)	2.54	4.90E-06	0.024 (12/503)	0.008 (56/6632)	2.99	7.40E-04	0.022 (21/962)	0.009 (57/6641)	2.22	2.60E-03
HLA-DQB1*0502	0.045 (66/1465)	0.019 (251/13273)	1.93	5.40E-06	0.038 (19/503)	0.019 (123/6632)	1.65	5.10E-02	0.049 (47/962)	0.019 (128/6641)	2.1	3.70E-05
HLA-DRB1*1301	0.072 (105/1465)	0.11 (1455/13273)	0.62	5.50E-06	0.064 (32/503)	0.109 (721/6632)	0.56	1.70E-03	0.076 (73/962)	0.111 (734/6641)	0.65	8.10E-04
HLA-B*3501	0.138 (202/1465)	0.101 (1342/13273)	1.44	7.20E-06	0.121 (61/503)	0.1 (662/6632)	1.24	1.30E-01	0.147 (141/962)	0.102 (680/6641)	1.55	1.40E-05
HLA-DRB1*0101	0.208 (305/1465)	0.173 (2290/13273)	1.36	7.50E-06	0.211 (106/503)	0.173 (1148/6632)	1.34	1.10E-02	0.207 (199/962)	0.172 (1142/6641)	1.38	2.00E-04
HLA-DRB3*0202	0.378 (554/1465)	0.288 (3827/13273)	1.3	7.80E-06	0.342 (172/503)	0.282 (1873/6632)	1.17	1.20E-01	0.397 (382/962)	0.294 (1954/6641)	1.36	3.80E-05
HLA-DPB1*1101	0.022 (32/1465)	0.049 (653/13273)	0.44	8.00E-06	0.032 (16/503)	0.051 (335/6632)	0.61	6.20E-02	0.017 (16/962)	0.048 (318/6641)	0.36	6.60E-05
HLA-DRB1*0801	0.065 (95/1465)	0.044 (584/13273)	1.65	1.40E-05	0.07 (35/503)	0.042 (281/6632)	1.85	1.00E-03	0.062 (60/962)	0.046 (303/6641)	1.5	6.10E-03
HLA-C*0401	0.238 (348/1465)	0.175 (2326/13273)	1.33	1.60E-05	0.211 (106/503)	0.175 (1161/6632)	1.14	2.60E-01	0.252 (242/962)	0.175 (1165/6641)	1.47	3.90E-06
HLA-DRB5*0202	0.038 (55/1465)	0.016 (210/13273)	1.96	2.10E-05	0.028 (14/503)	0.016 (103/6632)	1.43	2.30E-01	0.043 (41/962)	0.016 (107/6641)	2.29	1.70E-05
HLA-DQB1*0301	0.408 (598/1465)	0.334 (4434/13273)	1.27	2.80E-05	0.406 (204/503)	0.337 (2232/6632)	1.27	1.30E-02	0.41 (394/962)	0.332 (2202/6641)	1.28	7.40E-04
HLA-DRB1*1601	0.035 (52/1465)	0.015 (201/13273)	1.93	4.80E-05	0.028 (14/503)	0.015 (99/6632)	1.51	1.60E-01	0.04 (38/962)	0.015 (102/6641)	2.18	8.70E-05
HLA-B*1801	0.119 (174/1465)	0.079 (1054/13273)	1.41	9.20E-05	0.137 (69/503)	0.081 (534/6632)	1.68	1.80E-04	0.109 (105/962)	0.078 (520/6641)	1.29	2.80E-02
HLA-C*1203	0.12 (176/1465)	0.073 (973/13273)	1.42	1.10E-04	0.113 (57/503)	0.071 (469/6632)	1.47	1.20E-02	0.124 (119/962)	0.076 (504/6641)	1.36	6.40E-03
HLA-DRB1*0804	0.01 (14/1465)	0.002 (26/13273)	3.73	1.30E-04	0.008 (4/503)	0.002 (15/6632)	2.74	8.00E-02	0.01 (10/962)	0.002 (11/6641)	4.81	5.30E-04
HLA-DRB1*0402	0.031 (46/1465)	0.009 (123/13273)	2	2.00E-04	0.024 (12/503)	0.01 (65/6632)	1.73	9.60E-02	0.035 (34/962)	0.009 (58/6641)	2.15	1.20E-03
HLA-C*0602	0.129 (189/1465)	0.167 (2219/13273)	0.74	2.40E-04	0.127 (64/503)	0.165 (1091/6632)	0.74	2.70E-02	0.13 (125/962)	0.17 (1128/6641)	0.74	3.00E-03
HLA-DQB1*0501	0.268 (393/1465)	0.228 (3020/13273)	1.26	2.70E-04	0.268 (135/503)	0.227 (1508/6632)	1.24	4.40E-02	0.268 (258/962)	0.228 (1512/6641)	1.28	2.00E-03
HLA-A*2601	0.076 (112/1465)	0.045 (601/13273)	1.42	1.60E-03	0.064 (32/503)	0.044 (294/6632)	1.24	2.70E-01	0.083 (80/962)	0.046 (307/6641)	1.48	3.80E-03
HLA-DPB1*0201	0.213 (312/1465)	0.237 (3152/13273)	0.81	1.70E-03	0.193 (97/503)	0.234 (1555/6632)	0.74	1.10E-02	0.223 (215/962)	0.24 (1597/6641)	0.84	4.30E-02
HLA-DQB1*0303	0.069 (101/1465)	0.098 (1295/13273)	0.72	2.20E-03	0.074 (37/503)	0.095 (632/6632)	0.78	1.70E-01	0.067 (64/962)	0.1 (663/6641)	0.68	4.70E-03
HLA-C*0501	0.163 (239/1465)	0.205 (2727/13273)	0.8	2.20E-03	0.171 (86/503)	0.204 (1355/6632)	0.84	1.60E-01	0.159 (153/962)	0.207 (1372/6641)	0.77	5.00E-03
HLA-DQA1*0101	0.306 (448/1465)	0.27 (3588/13273)	1.2	3.00E-03	0.304 (153/503)	0.269 (1783/6632)	1.17	1.20E-01	0.307 (295/962)	0.272 (1805/6641)	1.22	9.40E-03
HLA-C*1604	0.005 (8/1465)	0.001 (11/13273)	4.29	3.00E-03	0.004 (2/503)	0.001 (4/6632)	4.9	8.30E-02	0.006 (6/962)	0.001 (7/6641)	3.63	2.90E-02
HLA-DRB1*0401	0.158 (231/1465)	0.206 (2731/13273)	0.8	3.20E-03	0.183 (92/503)	0.209 (1383/6632)	0.93	5.20E-01	0.144 (139/962)	0.203 (1348/6641)	0.74	2.20E-03
HLA-B*5701	0.051 (75/1465)	0.072 (962/13273)	0.71	5.20E-03	0.048 (24/503)	0.071 (470/6632)	0.67	6.20E-02	0.053 (51/962)	0.074 (492/6641)	0.71	2.80E-02
HLA-B*5108	0.005 (7/1465)	0.001 (14/13273)	3.75	5.50E-03	0.004 (2/503)	0.001 (8/6632)	2.24	3.20E-01	0.005 (5/962)	0.001 (6/6641)	6.24	2.80E-03
HLA-DRB1*1101	0.134 (196/1465)	0.097 (1292/13273)	1.25	6.90E-03	0.131 (66/503)	0.094 (626/6632)	1.29	7.30E-02	0.135 (130/962)	0.1 (666/6641)	1.21	6.80E-02
HLA-DPB1*0301	0.231 (338/1465)	0.205 (2718/13273)	1.19	8.20E-03	0.223 (112/503)	0.205 (1361/6632)	1.13	2.70E-01	0.235 (226/962)	0.204 (1357/6641)	1.22	1.80E-02
HLA-B*3502	0.037 (54/1465)	0.014 (184/13273)	1.55	8.70E-03	0.024 (12/503)	0.015 (99/6632)	0.94	8.60E-01	0.044 (42/962)	0.013 (85/6641)	2.08	3.40E-04
HLA-DRB4*0103	0.371 (543/1465)	0.416 (5525/13273)	0.87	1.50E-02	0.392 (197/503)	0.416 (2761/6632)	0.95	5.70E-01	0.36 (346/962)	0.416 (2764/6641)	0.83	1.00E-02
HLA-B*1401	0.014 (21/1465)	0.026 (340/13273)	0.58	1.60E-02	0.014 (7/503)	0.028 (183/6632)	0.52	8.80E-02	0.015 (14/962)	0.024 (157/6641)	0.63	1.00E-01
HLA-B*3906	0.02 (30/1465)	0.014 (188/13273)	1.6	1.90E-02	0.026 (13/503)	0.014 (96/6632)	1.91	3.10E-02	0.018 (17/962)	0.014 (92/6641)	1.41	2.00E-01
HLA-DPB1*0402	0.233 (342/1465)	0.208 (2760/13273)	1.16	2.10E-02	0.235 (118/503)	0.211 (1399/6632)	1.16	1.80E-01	0.233 (224/962)	0.205 (1361/6641)	1.17	5.80E-02

**HLA-DRB1*1503**	0.003 (4/1465)	0.001 (7/13273)	4.28	2.50E-02	0 (0/503)	0 (2/6632)	NA	9.60E-01	0.004 (4/962)	0.001 (5/6641)	4.61	2.90E-02
**HLA-DPB1*1501**	0.009 (13/1465)	0.015 (197/13273)	0.52	2.60E-02	0.002 (1/503)	0.016 (107/6632)	0.11	2.90E-02	0.012 (12/962)	0.014 (90/6641)	0.79	4.60E-01
**HLA-B*1302**	0.027 (40/1465)	0.038 (502/13273)	0.69	2.70E-02	0.022 (11/503)	0.036 (239/6632)	0.58	7.80E-02	0.03 (29/962)	0.04 (263/6641)	0.73	1.20E-01
**HLA-B*3503**	0.034 (50/1465)	0.023 (301/13273)	1.41	3.00E-02	0.04 (20/503)	0.022 (146/6632)	1.69	3.30E-02	0.031 (30/962)	0.023 (155/6641)	1.29	2.20E-01
**HLA-DPB1*1401**	0.039 (57/1465)	0.027 (360/13273)	1.37	3.30E-02	0.028 (14/503)	0.028 (189/6632)	0.89	6.70E-01	0.045 (43/962)	0.026 (171/6641)	1.73	2.00E-03
**HLA-DRB1*1103**	0.012 (18/1465)	0.007 (87/13273)	1.76	3.40E-02	0.008 (4/503)	0.007 (45/6632)	1.13	8.10E-01	0.015 (14/962)	0.006 (42/6641)	2.05	2.40E-02
**HLA-C*0701**	0.332 (486/1465)	0.309 (4100/13273)	1.13	3.40E-02	0.36 (181/503)	0.307 (2034/6632)	1.29	8.70E-03	0.317 (305/962)	0.311 (2066/6641)	1.06	4.60E-01
**HLA-B*4006**	0.003 (4/1465)	0.001 (10/13273)	3.45	3.90E-02	0.006 (3/503)	0.001 (5/6632)	8.49	3.90E-03	0.001 (1/962)	0.001 (5/6641)	1.01	9.90E-01
**HLA-B*4405**	0.008 (12/1465)	0.003 (43/13273)	1.97	4.40E-02	0.01 (5/503)	0.003 (18/6632)	2.71	5.40E-02	0.007 (7/962)	0.004 (25/6641)	1.6	2.90E-01
**HLA-C*1502**	0.048 (70/1465)	0.035 (470/13273)	1.31	4.40E-02	0.044 (22/503)	0.037 (244/6632)	1.15	5.50E-01	0.05 (48/962)	0.034 (226/6641)	1.43	3.10E-02
**HLA-B*1518**	0.002 (3/1465)	0.006 (85/13273)	0.31	4.50E-02	0.002 (1/503)	0.005 (35/6632)	0.36	3.20E-01	0.002 (2/962)	0.008 (50/6641)	0.27	6.90E-02
**HLA-A*2402**	0.186 (273/1465)	0.158 (2092/13273)	1.15	4.60E-02	0.197 (99/503)	0.158 (1045/6632)	1.26	5.00E-02	0.181 (174/962)	0.158 (1047/6641)	1.09	3.30E-01
**HLA-A*6901**	0.001 (1/1465)	0.002 (33/13273)	0.13	4.90E-02	0 (0/503)	0.002 (11/6632)	NA	9.60E-01	0.001 (1/962)	0.003 (22/6641)	0.14	5.50E-02
**HLA-B*1501**	0.097 (142/1465)	0.123 (1639/13273)	0.83	4.90E-02	0.093 (47/503)	0.123 (816/6632)	0.79	1.40E-01	0.099 (95/962)	0.124 (823/6641)	0.85	1.50E-01
**HLA-DPB1*0202**	0.009 (13/1465)	0.014 (192/13273)	0.57	5.20E-02	0.004 (2/503)	0.012 (77/6632)	0.33	1.30E-01	0.011 (11/962)	0.017 (115/6641)	0.6	1.10E-01
**HLA-A*6601**	0.012 (18/1465)	0.007 (88/13273)	1.65	6.00E-02	0.008 (4/503)	0.007 (45/6632)	1.13	8.10E-01	0.015 (14/962)	0.006 (43/6641)	1.87	5.10E-02
**HLA-A*0302**	0.01 (15/1465)	0.004 (53/13273)	1.77	6.10E-02	0.008 (4/503)	0.003 (18/6632)	2.31	1.40E-01	0.011 (11/962)	0.005 (35/6641)	1.38	3.70E-01
**HLA-DPA1*0104**	0.007 (10/1465)	0.011 (143/13273)	0.54	6.20E-02	0.002 (1/503)	0.012 (80/6632)	0.14	5.50E-02	0.009 (9/962)	0.009 (63/6641)	0.8	5.50E-01
**HLA-A*3002**	0.016 (24/1465)	0.021 (282/13273)	0.67	6.30E-02	0.01 (5/503)	0.018 (118/6632)	0.5	1.30E-01	0.02 (19/962)	0.025 (164/6641)	0.69	1.40E-01
**HLA-C*1402**	0.027 (40/1465)	0.018 (241/13273)	1.38	6.50E-02	0.03 (15/503)	0.016 (103/6632)	1.8	3.90E-02	0.026 (25/962)	0.021 (138/6641)	1.14	5.70E-01
**HLA-DPB1*1601**	0.006 (9/1465)	0.012 (163/13273)	0.53	6.60E-02	0.006 (3/503)	0.013 (85/6632)	0.47	2.00E-01	0.006 (6/962)	0.012 (78/6641)	0.58	2.00E-01
**HLA-B*5001**	0.016 (23/1465)	0.02 (266/13273)	0.67	6.80E-02	0.022 (11/503)	0.02 (134/6632)	0.92	8.00E-01	0.012 (12/962)	0.02 (132/6641)	0.54	4.80E-02
**HLA-DRB1*1201**	0.023 (34/1465)	0.032 (421/13273)	0.72	7.20E-02	0.024 (12/503)	0.032 (210/6632)	0.75	3.40E-01	0.023 (22/962)	0.032 (211/6641)	0.7	1.20E-01
**HLA-B*4901**	0.021 (31/1465)	0.026 (349/13273)	0.71	7.20E-02	0.022 (11/503)	0.026 (174/6632)	0.73	3.30E-01	0.021 (20/962)	0.026 (175/6641)	0.7	1.40E-01
**HLA-DRB3*0101**	0.259 (380/1465)	0.296 (3924/13273)	0.89	7.30E-02	0.272 (137/503)	0.292 (1939/6632)	0.96	7.30E-01	0.253 (243/962)	0.299 (1985/6641)	0.85	4.40E-02
**HLA-DQB1*0604**	0.052 (76/1465)	0.063 (833/13273)	0.8	7.40E-02	0.042 (21/503)	0.06 (395/6632)	0.68	9.90E-02	0.057 (55/962)	0.066 (438/6641)	0.84	2.30E-01
**HLA-DRB3*0301**	0.073 (107/1465)	0.085 (1133/13273)	0.83	7.90E-02	0.062 (31/503)	0.082 (543/6632)	0.73	1.00E-01	0.079 (76/962)	0.089 (590/6641)	0.86	2.60E-01
**HLA-B*3508**	0.017 (25/1465)	0.009 (113/13273)	1.46	9.80E-02	0.016 (8/503)	0.008 (56/6632)	1.45	3.40E-01	0.018 (17/962)	0.009 (57/6641)	1.48	1.80E-01
**HLA-B*0801**	0.246 (361/1465)	0.244 (3236/13273)	1.11	1.00E-01	0.256 (129/503)	0.243 (1611/6632)	1.17	1.50E-01	0.241 (232/962)	0.245 (1625/6641)	1.09	3.10E-01
**HLA-DRB1*0407**	0.028 (41/1465)	0.023 (302/13273)	1.31	1.10E-01	0.03 (15/503)	0.023 (154/6632)	1.37	2.50E-01	0.027 (26/962)	0.022 (148/6641)	1.3	2.30E-01
**HLA-B*4402**	0.175 (256/1465)	0.205 (2721/13273)	0.89	1.10E-01	0.183 (92/503)	0.205 (1361/6632)	0.93	5.50E-01	0.17 (164/962)	0.205 (1360/6641)	0.87	1.30E-01
**HLA-DRB1*1302**	0.076 (111/1465)	0.087 (1150/13273)	0.85	1.20E-01	0.064 (32/503)	0.083 (549/6632)	0.75	1.20E-01	0.082 (79/962)	0.09 (601/6641)	0.88	3.30E-01
**HLA-DRB1*0901**	0.017 (25/1465)	0.025 (331/13273)	0.72	1.20E-01	0.02 (10/503)	0.024 (157/6632)	0.9	7.50E-01	0.016 (15/962)	0.026 (174/6641)	0.62	7.70E-02
**HLA-DPA1*0201**	0.3 (440/1465)	0.275 (3652/13273)	1.1	1.20E-01	0.3 (151/503)	0.278 (1842/6632)	1.07	5.10E-01	0.3 (289/962)	0.273 (1810/6641)	1.14	8.10E-02
**HLA-C*1602**	0.008 (12/1465)	0.004 (57/13273)	1.65	1.20E-01	0.006 (3/503)	0.004 (26/6632)	1.2	7.60E-01	0.009 (9/962)	0.005 (31/6641)	1.92	9.40E-02
**HLA-A*0203**	0.003 (4/1465)	0.001 (17/13273)	2.36	1.30E-01	0.002 (1/503)	0.002 (10/6632)	1.39	7.50E-01	0.003 (3/962)	0.001 (7/6641)	3.32	8.50E-02
**HLA-C*0303**	0.088 (129/1465)	0.106 (1404/13273)	0.86	1.30E-01	0.089 (45/503)	0.109 (722/6632)	0.85	3.10E-01	0.087 (84/962)	0.103 (682/6641)	0.89	3.20E-01
**HLA-A*3201**	0.059 (86/1465)	0.07 (933/13273)	0.84	1.40E-01	0.054 (27/503)	0.073 (486/6632)	0.72	1.10E-01	0.061 (59/962)	0.067 (447/6641)	0.93	6.20E-01
**HLA-DPA1*0301**	0.003 (4/1465)	0.001 (10/13273)	2.49	1.40E-01	0 (0/503)	0.001 (4/6632)	NA	9.70E-01	0.004 (4/962)	0.001 (6/6641)	3.45	6.70E-02
**HLA-DRB3*9901**	0.838 (1227/1465)	0.863 (11459/13273)	0.89	1.40E-01	0.853 (429/503)	0.865 (5736/6632)	0.98	8.80E-01	0.83 (798/962)	0.862 (5723/6641)	0.86	1.10E-01
**HLA-B*4501**	0.011 (16/1465)	0.015 (197/13273)	0.69	1.60E-01	0.008 (4/503)	0.015 (102/6632)	0.49	1.60E-01	0.012 (12/962)	0.014 (95/6641)	0.82	5.30E-01
**HLA-DPB1*1701**	0.023 (34/1465)	0.025 (332/13273)	0.78	1.80E-01	0.026 (13/503)	0.023 (155/6632)	0.96	8.80E-01	0.022 (21/962)	0.027 (177/6641)	0.68	9.90E-02
**HLA-B*4002**	0.026 (38/1465)	0.021 (278/13273)	1.27	1.80E-01	0.012 (6/503)	0.021 (138/6632)	0.56	1.70E-01	0.033 (32/962)	0.021 (140/6641)	1.7	8.60E-03
**HLA-C*0802**	0.074 (109/1465)	0.079 (1047/13273)	0.87	1.80E-01	0.062 (31/503)	0.084 (555/6632)	0.66	3.20E-02	0.081 (78/962)	0.074 (492/6641)	1.03	8.20E-01
**HLA-DPA1*0202**	0.051 (74/1465)	0.061 (816/13273)	0.85	1.90E-01	0.05 (25/503)	0.064 (425/6632)	0.78	2.40E-01	0.051 (49/962)	0.059 (391/6641)	0.91	5.30E-01
**HLA-DPB1*0901**	0.018 (26/1465)	0.013 (179/13273)	1.32	2.00E-01	0.026 (13/503)	0.014 (91/6632)	1.83	4.50E-02	0.014 (13/962)	0.013 (88/6641)	1.05	8.70E-01
**HLA-DPB1*1001**	0.041 (60/1465)	0.034 (453/13273)	1.18	2.40E-01	0.046 (23/503)	0.034 (223/6632)	1.32	2.20E-01	0.038 (37/962)	0.035 (230/6641)	1.12	5.30E-01
**HLA-DQA1*0301**	0.33 (484/1465)	0.357 (4735/13273)	0.93	2.50E-01	0.342 (172/503)	0.358 (2373/6632)	0.98	8.40E-01	0.324 (312/962)	0.356 (2362/6641)	0.91	2.20E-01
**HLA-DRB1*0403**	0.016 (24/1465)	0.011 (144/13273)	1.29	2.60E-01	0.016 (8/503)	0.012 (78/6632)	1.25	5.50E-01	0.017 (16/962)	0.01 (66/6641)	1.36	2.90E-01
**HLA-DQB1*0302**	0.206 (302/1465)	0.198 (2625/13273)	1.08	2.70E-01	0.195 (98/503)	0.199 (1317/6632)	1	9.90E-01	0.212 (204/962)	0.197 (1308/6641)	1.12	1.90E-01
**HLA-DRB5*9901**	0.975 (1428/1465)	0.978 (12976/13273)	0.82	2.70E-01	0.978 (492/503)	0.979 (6490/6632)	0.91	7.70E-01	0.973 (936/962)	0.977 (6486/6641)	0.78	2.60E-01
**HLA-DRB1*1102**	0.01 (15/1465)	0.006 (83/13273)	1.37	2.70E-01	0.004 (2/503)	0.006 (42/6632)	0.54	4.00E-01	0.014 (13/962)	0.006 (41/6641)	1.76	8.60E-02
**HLA-A*3402**	0.001 (1/1465)	0.002 (26/13273)	0.33	2.70E-01	0 (0/503)	0.002 (14/6632)	NA	9.60E-01	0.001 (1/962)	0.002 (12/6641)	0.61	6.40E-01
**HLA-DRB1*1001**	0.02 (29/1465)	0.014 (187/13273)	1.25	2.70E-01	0.018 (9/503)	0.012 (82/6632)	1.34	4.10E-01	0.021 (20/962)	0.016 (105/6641)	1.15	5.80E-01
**HLA-DRB1*1602**	0.004 (6/1465)	0.002 (25/13273)	1.65	2.80E-01	0.002 (1/503)	0.002 (12/6632)	0.77	8.00E-01	0.005 (5/962)	0.002 (13/6641)	2.31	1.20E-01

**HLA-A*3001**	0.02 (29/1465)	0.022 (290/13273)	0.81	2.90E-01	0.024 (12/503)	0.02 (132/6632)	1.04	8.90E-01	0.018 (17/962)	0.024 (158/6641)	0.68	1.40E-01
**HLA-DRB1*0803**	0.005 (8/1465)	0.004 (48/13273)	1.49	3.10E-01	0.006 (3/503)	0.004 (26/6632)	1.59	4.50E-01	0.005 (5/962)	0.003 (22/6641)	1.51	4.10E-01
**HLA-C*0704**	0.03 (44/1465)	0.036 (477/13273)	0.85	3.20E-01	0.022 (11/503)	0.037 (245/6632)	0.59	9.60E-02	0.034 (33/962)	0.035 (232/6641)	1.01	9.40E-01
**HLA-B*1503**	0.002 (3/1465)	0.003 (37/13273)	0.55	3.30E-01	0 (0/503)	0.003 (17/6632)	NA	9.50E-01	0.003 (3/962)	0.003 (20/6641)	0.78	6.90E-01
**HLA-C*1505**	0.01 (14/1465)	0.004 (58/13273)	1.35	3.30E-01	0.01 (5/503)	0.004 (28/6632)	1.64	3.20E-01	0.009 (9/962)	0.005 (30/6641)	1.22	6.20E-01
**HLA-B*3801**	0.041 (60/1465)	0.025 (328/13273)	1.16	3.30E-01	0.028 (14/503)	0.023 (153/6632)	0.91	7.50E-01	0.048 (46/962)	0.026 (175/6641)	1.21	2.90E-01
**HLA-DPA1*0105**	0.003 (4/1465)	0.002 (20/13273)	1.67	3.60E-01	0.002 (1/503)	0.001 (9/6632)	1.38	7.60E-01	0.003 (3/962)	0.002 (11/6641)	1.79	3.90E-01
**HLA-B*1516**	0.001 (1/1465)	0.001 (17/13273)	0.4	3.70E-01	0 (0/503)	0.001 (6/6632)	NA	9.60E-01	0.001 (1/962)	0.002 (11/6641)	0.51	5.30E-01
**HLA-B*3901**	0.027 (39/1465)	0.022 (296/13273)	1.16	3.80E-01	0.024 (12/503)	0.022 (143/6632)	1.11	7.40E-01	0.028 (27/962)	0.023 (153/6641)	1.15	5.10E-01
**HLA-DPB1*0401**	0.663 (971/1465)	0.68 (9031/13273)	0.95	3.90E-01	0.682 (343/503)	0.677 (4491/6632)	1.04	6.60E-01	0.653 (628/962)	0.684 (4540/6641)	0.89	1.30E-01
**HLA-DRB1*0102**	0.027 (40/1465)	0.023 (306/13273)	0.86	4.00E-01	0.024 (12/503)	0.024 (161/6632)	0.71	2.70E-01	0.029 (28/962)	0.022 (145/6641)	1.02	9.30E-01
**HLA-DRB1*1303**	0.018 (26/1465)	0.019 (254/13273)	0.84	4.10E-01	0.016 (8/503)	0.02 (131/6632)	0.72	3.70E-01	0.019 (18/962)	0.019 (123/6641)	0.91	7.20E-01
**HLA-A*0205**	0.02 (30/1465)	0.019 (256/13273)	0.85	4.10E-01	0.016 (8/503)	0.019 (124/6632)	0.71	3.60E-01	0.023 (22/962)	0.02 (132/6641)	0.9	6.50E-01
**HLA-DPB1*0601**	0.037 (54/1465)	0.035 (458/13273)	1.13	4.10E-01	0.036 (18/503)	0.032 (214/6632)	1.17	5.30E-01	0.037 (36/962)	0.037 (244/6641)	1.06	7.40E-01
**HLA-DRB1*1305**	0.002 (3/1465)	0.001 (19/13273)	0.6	4.20E-01	0 (0/503)	0.001 (7/6632)	NA	9.50E-01	0.003 (3/962)	0.002 (12/6641)	0.65	5.10E-01
**HLA-B*4001**	0.106 (155/1465)	0.109 (1441/13273)	1.08	4.20E-01	0.117 (59/503)	0.107 (710/6632)	1.21	2.00E-01	0.1 (96/962)	0.11 (731/6641)	0.99	9.40E-01
**HLA-C*1701**	0.024 (35/1465)	0.015 (204/13273)	1.16	4.40E-01	0.018 (9/503)	0.015 (99/6632)	0.97	9.30E-01	0.027 (26/962)	0.016 (105/6641)	1.22	3.90E-01
**HLA-DQA1*0601**	0.005 (8/1465)	0.004 (53/13273)	1.34	4.50E-01	0.006 (3/503)	0.005 (30/6632)	1.32	6.50E-01	0.005 (5/962)	0.003 (23/6641)	1.5	4.20E-01
**HLA-A*3303**	0.006 (9/1465)	0.004 (54/13273)	1.31	4.60E-01	0.006 (3/503)	0.004 (29/6632)	1.24	7.30E-01	0.006 (6/962)	0.004 (25/6641)	1.38	4.90E-01
**HLA-DRB1*0103**	0.025 (37/1465)	0.031 (407/13273)	0.88	4.70E-01	0.028 (14/503)	0.03 (197/6632)	0.97	9.10E-01	0.024 (23/962)	0.032 (210/6641)	0.82	3.80E-01
**HLA-DQB1*0504**	0.001 (2/1465)	0.003 (34/13273)	0.6	4.80E-01	0 (0/503)	0.003 (17/6632)	0	9.60E-01	0.002 (2/962)	0.003 (17/6641)	0.92	9.10E-01
**HLA-A*3101**	0.048 (70/1465)	0.052 (694/13273)	0.91	4.80E-01	0.046 (23/503)	0.051 (341/6632)	0.88	5.50E-01	0.049 (47/962)	0.053 (353/6641)	0.91	5.60E-01
**HLA-B*3701**	0.024 (35/1465)	0.028 (376/13273)	0.88	4.80E-01	0.028 (14/503)	0.027 (181/6632)	1.05	8.60E-01	0.022 (21/962)	0.029 (195/6641)	0.79	3.00E-01
**HLA-A*3004**	0.005 (7/1465)	0.003 (36/13273)	1.35	4.80E-01	0.008 (4/503)	0.004 (24/6632)	1.75	3.10E-01	0.003 (3/962)	0.002 (12/6641)	1.29	7.00E-01
**HLA-B*5801**	0.016 (23/1465)	0.011 (144/13273)	1.16	5.10E-01	0.018 (9/503)	0.011 (76/6632)	1.28	4.90E-01	0.015 (14/962)	0.01 (68/6641)	1.12	7.00E-01
**HLA-DRB1*0802**	0.002 (3/1465)	0.002 (20/13273)	1.49	5.20E-01	0.002 (1/503)	0.002 (10/6632)	1.42	7.40E-01	0.002 (2/962)	0.002 (10/6641)	1.4	6.70E-01
**HLA-C*0702**	0.257 (377/1465)	0.27 (3581/13273)	1.04	5.30E-01	0.29 (146/503)	0.272 (1803/6632)	1.2	8.50E-02	0.24 (231/962)	0.268 (1778/6641)	0.96	6.60E-01
**HLA-A*2301**	0.031 (45/1465)	0.032 (423/13273)	0.9	5.30E-01	0.038 (19/503)	0.032 (215/6632)	1.11	6.80E-01	0.027 (26/962)	0.031 (208/6641)	0.82	3.40E-01
**HLA-B*4102**	0.01 (15/1465)	0.009 (126/13273)	0.84	5.50E-01	0.002 (1/503)	0.009 (62/6632)	0.19	9.90E-02	0.015 (14/962)	0.01 (64/6641)	1.09	7.80E-01
**HLA-A*0202**	0.003 (5/1465)	0.002 (22/13273)	1.36	5.50E-01	0 (0/503)	0.002 (10/6632)	NA	9.60E-01	0.005 (5/962)	0.002 (12/6641)	1.75	3.20E-01
**HLA-C*0102**	0.072 (106/1465)	0.068 (907/13273)	1.07	5.50E-01	0.072 (36/503)	0.063 (419/6632)	1.15	4.40E-01	0.073 (70/962)	0.073 (488/6641)	0.98	8.70E-01
**HLA-DRB1*1502**	0.012 (17/1465)	0.01 (131/13273)	0.85	5.50E-01	0.012 (6/503)	0.011 (72/6632)	0.88	7.80E-01	0.011 (11/962)	0.009 (59/6641)	0.87	6.80E-01
**HLA-DPB1*1901**	0.008 (12/1465)	0.011 (140/13273)	0.84	5.50E-01	0.006 (3/503)	0.009 (61/6632)	0.66	4.90E-01	0.009 (9/962)	0.012 (79/6641)	0.85	6.60E-01
**HLA-A*2501**	0.042 (62/1465)	0.04 (527/13273)	1.08	5.60E-01	0.056 (28/503)	0.04 (268/6632)	1.41	9.50E-02	0.035 (34/962)	0.039 (259/6641)	0.93	7.10E-01
**HLA-DQB1*0602**	0.222 (325/1465)	0.246 (3271/13273)	0.96	5.60E-01	0.241 (121/503)	0.248 (1643/6632)	1.05	6.30E-01	0.212 (204/962)	0.245 (1628/6641)	0.91	2.50E-01
**HLA-DQB1*0601**	0.012 (17/1465)	0.01 (130/13273)	0.86	5.70E-01	0.014 (7/503)	0.01 (69/6632)	1.08	8.50E-01	0.01 (10/962)	0.009 (61/6641)	0.76	4.30E-01
**HLA-A*0301**	0.255 (373/1465)	0.26 (3456/13273)	1.04	5.70E-01	0.247 (124/503)	0.259 (1718/6632)	0.99	9.30E-01	0.259 (249/962)	0.262 (1738/6641)	1.07	4.30E-01
**HLA-DQB1*0201**	0.251 (368/1465)	0.257 (3409/13273)	1.04	5.80E-01	0.278 (140/503)	0.253 (1678/6632)	1.2	8.10E-02	0.237 (228/962)	0.261 (1731/6641)	0.95	5.60E-01
**HLA-DRB1*0408**	0.008 (11/1465)	0.006 (86/13273)	1.19	5.90E-01	0.01 (5/503)	0.007 (45/6632)	1.62	3.10E-01	0.006 (6/962)	0.006 (41/6641)	1	9.90E-01
**HLA-B*0702**	0.227 (332/1465)	0.251 (3334/13273)	0.97	6.10E-01	0.258 (130/503)	0.253 (1679/6632)	1.12	2.80E-01	0.21 (202/962)	0.249 (1655/6641)	0.89	1.60E-01
**HLA-B*5501**	0.033 (48/1465)	0.035 (460/13273)	0.93	6.30E-01	0.034 (17/503)	0.037 (244/6632)	0.92	7.60E-01	0.032 (31/962)	0.033 (216/6641)	0.94	7.60E-01
**HLA-DPB1*2301**	0.003 (4/1465)	0.004 (49/13273)	0.78	6.30E-01	0.004 (2/503)	0.003 (19/6632)	1.48	6.00E-01	0.002 (2/962)	0.005 (30/6641)	0.47	3.10E-01
**HLA-DPB1*0101**	0.103 (151/1465)	0.113 (1502/13273)	0.96	6.50E-01	0.099 (50/503)	0.117 (773/6632)	0.87	3.90E-01	0.105 (101/962)	0.11 (729/6641)	1.03	7.60E-01
**HLA-DRB1*0301**	0.247 (362/1465)	0.254 (3376/13273)	1.03	6.50E-01	0.27 (136/503)	0.251 (1667/6632)	1.17	1.40E-01	0.235 (226/962)	0.257 (1709/6641)	0.96	6.20E-01
**HLA-C*0304**	0.143 (210/1465)	0.154 (2039/13273)	1.04	6.60E-01	0.143 (72/503)	0.152 (1009/6632)	1.02	8.50E-01	0.143 (138/962)	0.155 (1030/6641)	1.03	8.00E-01
**HLA-A*3301**	0.016 (24/1465)	0.014 (192/13273)	0.91	6.70E-01	0.008 (4/503)	0.016 (104/6632)	0.4	7.30E-02	0.021 (20/962)	0.013 (88/6641)	1.33	2.60E-01
**HLA-B*5101**	0.091 (133/1465)	0.083 (1096/13273)	1.04	6.90E-01	0.082 (41/503)	0.081 (534/6632)	0.95	7.60E-01	0.096 (92/962)	0.085 (562/6641)	1.07	5.70E-01
**HLA-B*1402**	0.061 (90/1465)	0.057 (756/13273)	0.96	7.00E-01	0.048 (24/503)	0.06 (397/6632)	0.68	8.20E-02	0.069 (66/962)	0.054 (359/6641)	1.17	2.70E-01
**HLA-DRB1*0404**	0.081 (119/1465)	0.089 (1183/13273)	0.96	7.00E-01	0.07 (35/503)	0.088 (586/6632)	0.8	2.10E-01	0.087 (84/962)	0.09 (597/6641)	1.06	6.60E-01
**HLA-B*5301**	0.009 (13/1465)	0.006 (81/13273)	1.11	7.40E-01	0.004 (2/503)	0.007 (49/6632)	0.42	2.40E-01	0.011 (11/962)	0.005 (32/6641)	1.83	9.30E-02
**HLA-A*0201**	0.473 (693/1465)	0.485 (6438/13273)	1.02	7.40E-01	0.477 (240/503)	0.487 (3229/6632)	1.01	9.40E-01	0.471 (453/962)	0.483 (3209/6641)	1.03	6.90E-01
**HLA-A*6801**	0.057 (84/1465)	0.063 (830/13273)	0.96	7.40E-01	0.048 (24/503)	0.063 (416/6632)	0.79	2.70E-01	0.062 (60/962)	0.062 (414/6641)	1.06	7.00E-01
**HLA-B*5201**	0.016 (24/1465)	0.011 (152/13273)	1.08	7.40E-01	0.016 (8/503)	0.012 (80/6632)	1.06	8.80E-01	0.017 (16/962)	0.011 (72/6641)	1.12	7.00E-01
**HLA-DQA1*0102**	0.335 (491/1465)	0.341 (4527/13273)	1.02	7.50E-01	0.338 (170/503)	0.339 (2246/6632)	1.05	6.40E-01	0.334 (321/962)	0.343 (2281/6641)	0.99	9.20E-01
**HLA-DQB1*0503**	0.049 (72/1465)	0.05 (662/13273)	0.96	7.50E-01	0.046 (23/503)	0.048 (319/6632)	0.9	6.50E-01	0.051 (49/962)	0.052 (343/6641)	0.98	9.00E-01

**HLA-DRB1*1404**	0.002 (3/1465)	0.002 (29/13273)	0.82	7.50E-01	0.004 (2/503)	0.002 (13/6632)	1.66	5.10E-01	0.001 (1/962)	0.002 (16/6641)	0.4	3.80E-01
**HLA-DRB1*0806**	0.001 (1/1465)	0.001 (10/13273)	0.71	7.50E-01	0.002 (1/503)	0.001 (4/6632)	3.55	2.60E-01	0 (0/962)	0.001 (6/6641)	NA	9.50E-01
**HLA-B*4701**	0.005 (7/1465)	0.006 (79/13273)	0.89	7.60E-01	0.006 (3/503)	0.006 (40/6632)	1.08	9.00E-01	0.004 (4/962)	0.006 (39/6641)	0.79	6.50E-01
**HLA-DPB1*0501**	0.04 (58/1465)	0.044 (580/13273)	0.96	7.80E-01	0.046 (23/503)	0.045 (299/6632)	1.05	8.30E-01	0.036 (35/962)	0.042 (281/6641)	0.95	7.90E-01
**HLA-C*0302**	0.004 (6/1465)	0.003 (45/13273)	1.13	7.90E-01	0.002 (1/503)	0.004 (24/6632)	0.49	4.90E-01	0.005 (5/962)	0.003 (21/6641)	1.54	4.00E-01
**HLA-DRB1*0405**	0.011 (16/1465)	0.011 (140/13273)	0.93	7.90E-01	0.016 (8/503)	0.011 (74/6632)	1.25	5.50E-01	0.008 (8/962)	0.01 (66/6641)	0.78	5.20E-01
**HLA-B*0705**	0.006 (9/1465)	0.004 (56/13273)	0.92	8.30E-01	0.002 (1/503)	0.004 (28/6632)	0.34	2.90E-01	0.008 (8/962)	0.004 (28/6641)	1.21	6.50E-01
**HLA-A*0101**	0.311 (455/1465)	0.323 (4282/13273)	0.99	8.30E-01	0.334 (168/503)	0.323 (2139/6632)	1.08	4.20E-01	0.298 (287/962)	0.323 (2143/6641)	0.95	4.70E-01
**HLA-DPA1*0103**	0.964 (1412/1465)	0.964 (12798/13273)	1.03	8.30E-01	0.966 (486/503)	0.961 (6375/6632)	1.25	3.90E-01	0.963 (926/962)	0.967 (6423/6641)	0.88	4.80E-01
**HLA-DRB1*1401**	0.048 (70/1465)	0.048 (635/13273)	0.98	8.50E-01	0.042 (21/503)	0.046 (305/6632)	0.88	5.70E-01	0.051 (49/962)	0.05 (330/6641)	1.01	9.50E-01
**HLA-DPB1*2001**	0.005 (7/1465)	0.006 (76/13273)	0.93	8.50E-01	0.006 (3/503)	0.006 (41/6632)	1.06	9.30E-01	0.004 (4/962)	0.005 (35/6641)	0.87	8.00E-01
**HLA-C*1202**	0.016 (23/1465)	0.011 (150/13273)	1.04	8.60E-01	0.016 (8/503)	0.012 (78/6632)	1.09	8.20E-01	0.016 (15/962)	0.011 (72/6641)	1.04	8.90E-01
**HLA-DQB1*0609**	0.022 (32/1465)	0.023 (302/13273)	0.97	8.60E-01	0.022 (11/503)	0.022 (147/6632)	0.98	9.50E-01	0.022 (21/962)	0.023 (155/6641)	0.96	8.50E-01
**HLA-DRB1*1501**	0.229 (336/1465)	0.248 (3292/13273)	0.99	8.60E-01	0.25 (126/503)	0.25 (1657/6632)	1.1	4.00E-01	0.218 (210/962)	0.246 (1635/6641)	0.93	3.70E-01
**HLA-C*0202**	0.081 (119/1465)	0.079 (1054/13273)	1.02	8.70E-01	0.064 (32/503)	0.08 (532/6632)	0.77	1.70E-01	0.09 (87/962)	0.079 (522/6641)	1.16	2.30E-01
**HLA-B*4101**	0.011 (16/1465)	0.007 (90/13273)	1.04	8.80E-01	0.012 (6/503)	0.006 (42/6632)	1.29	5.80E-01	0.01 (10/962)	0.007 (48/6641)	0.93	8.50E-01
**HLA-B*5703**	0.001 (2/1465)	0.001 (11/13273)	0.92	9.10E-01	0 (0/503)	0.001 (5/6632)	NA	9.60E-01	0.002 (2/962)	0.001 (6/6641)	1.29	7.60E-01
**HLA-B*1524**	0.001 (1/1465)	0.001 (12/13273)	0.9	9.20E-01	0 (0/503)	0.001 (5/6632)	NA	9.60E-01	0.001 (1/962)	0.001 (7/6641)	1.17	8.80E-01
**HLA-B*2702**	0.003 (5/1465)	0.003 (36/13273)	0.96	9.30E-01	0 (0/503)	0.003 (19/6632)	NA	9.50E-01	0.005 (5/962)	0.003 (17/6641)	1.48	4.60E-01
**HLA-A*0102**	0 (0/1465)	0.001 (10/13273)	NA	9.40E-01	0 (0/503)	0.001 (5/6632)	NA	9.60E-01	0 (0/962)	0.001 (5/6641)	NA	9.60E-01
**HLA-DRB1*0406**	0 (0/1465)	0.001 (10/13273)	NA	9.50E-01	0 (0/503)	0.001 (5/6632)	NA	9.60E-01	0 (0/962)	0.001 (5/6641)	NA	9.60E-01
**HLA-B*4801**	0.001 (2/1465)	0.001 (19/13273)	1.05	9.50E-01	0 (0/503)	0.002 (11/6632)	NA	9.60E-01	0.002 (2/962)	0.001 (8/6641)	1.63	5.40E-01
**HLA-B*4404**	0 (0/1465)	0.002 (22/13273)	NA	9.50E-01	0 (0/503)	0.002 (10/6632)	NA	9.70E-01	0 (0/962)	0.002 (12/6641)	NA	9.40E-01
**HLA-A*6802**	0.015 (22/1465)	0.013 (172/13273)	0.99	9.50E-01	0.01 (5/503)	0.013 (85/6632)	0.69	4.30E-01	0.018 (17/962)	0.013 (87/6641)	1.12	6.80E-01
**HLA-C*0801**	0.001 (2/1465)	0.002 (20/13273)	1.04	9.60E-01	0.002 (1/503)	0.002 (12/6632)	1.36	7.70E-01	0.001 (1/962)	0.001 (8/6641)	0.89	9.10E-01
**HLA-A*1101**	0.117 (172/1465)	0.118 (1570/13273)	1	9.60E-01	0.111 (56/503)	0.116 (772/6632)	0.95	7.30E-01	0.121 (116/962)	0.12 (798/6641)	1	9.80E-01
**HLA-A*2901**	0.008 (11/1465)	0.004 (55/13273)	1.02	9.60E-01	0.006 (3/503)	0.004 (26/6632)	1.02	9.80E-01	0.008 (8/962)	0.004 (29/6641)	1	9.90E-01
**HLA-DRB5*0101**	0.232 (340/1465)	0.248 (3292/13273)	1	9.70E-01	0.25 (126/503)	0.25 (1655/6632)	1.1	3.90E-01	0.222 (214/962)	0.246 (1637/6641)	0.95	5.10E-01
**HLA-B*1517**	0.007 (10/1465)	0.005 (62/13273)	0.99	9.70E-01	0.008 (4/503)	0.004 (25/6632)	1.54	4.30E-01	0.006 (6/962)	0.006 (37/6641)	0.73	4.80E-01
**HLA-DQB1*0304**	0.003 (5/1465)	0.003 (43/13273)	0.99	9.80E-01	0.006 (3/503)	0.003 (20/6632)	2.19	2.10E-01	0.002 (2/962)	0.003 (23/6641)	0.52	3.80E-01
**HLA-B*2705**	0.079 (116/1465)	0.085 (1124/13273)	1	9.80E-01	0.07 (35/503)	0.085 (564/6632)	0.85	3.50E-01	0.084 (81/962)	0.084 (560/6641)	1.09	4.80E-01
**HLA-A*0206**	0.003 (4/1465)	0.003 (40/13273)	1.01	9.90E-01	0 (0/503)	0.003 (20/6632)	NA	9.50E-01	0.004 (4/962)	0.003 (20/6641)	1.65	3.60E-01

Only alleles seen in >=10 controls in the meta-analysis cohort are shown. P-values below the GWS threshold are coloured orange, those with suggestive significance ($P<5x10^{-6}$) are coloured yellow. Rare alleles for which odds ratios could not be accurately computed are assigned OR = NA. CO = control, OR = odds ratio, NA = not applicable.

<u>Supplementary Table 4:</u> Classical HLA allele frequencies and disease associations with lSSc in all three study cohorts.

	ISSC vs CONTROL											
	Meta-analysis Cohort				Cohort 1				Cohort 2			
HLA Allele	ISSc Prop.(Count)	CO Prop.(Count)	OR	p	ISSc Prop.(Count)	CO Prop.(Count)	OR	p	ISSc Prop.(Count)	CO Prop.(Count)	OR	p
**HLA-DRB1*0701**	0.126 (123/974)	0.257 (3406/13273)	0.42	**2.20E-18**	0.15 (53/353)	0.262 (1737/6632)	0.49	**3.20E-06**	0.113 (70/621)	0.251 (1669/6641)	0.39	**3.30E-13**
**HLA-DQA1*0201**	0.127 (124/974)	0.256 (3397/13273)	0.43	**5.30E-18**	0.153 (54/353)	0.261 (1733/6632)	0.51	6.10E-06	0.113 (70/621)	0.251 (1664/6641)	0.39	**3.90E-13**
**HLA-DRB1*1104**	0.114 (111/974)	0.035 (459/13273)	2.76	**6.50E-18**	0.076 (27/353)	0.035 (230/6632)	1.94	0.0026	0.135 (84/621)	0.034 (229/6641)	3.26	**1.60E-16**
**HLA-DQB1*0202**	0.08 (78/974)	0.186 (2468/13273)	0.38	**5.00E-16**	0.096 (34/353)	0.193 (1282/6632)	0.44	6.50E-06	0.071 (44/621)	0.179 (1186/6641)	0.35	**4.20E-11**
**HLA-DRB4*0101**	0.094 (92/974)	0.188 (2491/13273)	0.45	**1.20E-12**	0.113 (40/353)	0.194 (1289/6632)	0.52	0.00014	0.084 (52/621)	0.181 (1202/6641)	0.42	**5.10E-09**
**HLA-B*4403**	0.038 (37/974)	0.105 (1397/13273)	0.34	**2.80E-10**	0.054 (19/353)	0.11 (728/6632)	0.46	0.001	0.029 (18/621)	0.101 (669/6641)	0.28	**1.60E-07**
**HLA-C*1601**	0.03 (29/974)	0.082 (1090/13273)	0.35	**2.90E-08**	0.042 (15/353)	0.086 (573/6632)	0.46	0.0037	0.023 (14/621)	0.078 (517/6641)	0.28	**4.20E-06**
**HLA-DRB4*9901**	0.937 (913/974)	0.883 (11721/13273)	1.93	**1.10E-06**	0.915 (323/353)	0.876 (5811/6632)	1.49	0.04	0.95 (590/621)	0.89 (5910/6641)	2.29	1.30E-05
**HLA-DQA1*0401**	0.078 (76/974)	0.047 (618/13273)	1.86	**1.10E-06**	0.085 (30/353)	0.045 (298/6632)	2.05	0.00035	0.074 (46/621)	0.048 (320/6641)	1.68	0.0018
**HLA-A*2902**	0.038 (37/974)	0.079 (1046/13273)	0.45	**3.50E-06**	0.037 (13/353)	0.083 (552/6632)	0.4	0.0016	0.039 (24/621)	0.074 (494/6641)	0.5	0.0011
**HLA-DRB1*0101**	0.221 (215/974)	0.173 (2290/13273)	1.46	**3.60E-06**	0.235 (83/353)	0.173 (1148/6632)	1.51	0.0015	0.213 (132/621)	0.172 (1142/6641)	1.44	0.00052
**HLA-DQB1*0402**	0.08 (78/974)	0.049 (645/13273)	1.79	**3.80E-06**	0.088 (31/353)	0.048 (316/6632)	1.97	6.00E-04	0.076 (47/621)	0.05 (329/6641)	1.62	0.0032
**HLA-DPB1*1101**	0.015 (15/974)	0.049 (653/13273)	0.31	7.60E-06	0.025 (9/353)	0.051 (335/6632)	0.49	0.035	0.01 (6/621)	0.048 (318/6641)	0.21	0.00014
**HLA-DRB1*0801**	0.071 (69/974)	0.044 (584/13273)	1.8	1.00E-05	0.079 (28/353)	0.042 (281/6632)	2.05	0.00054	0.066 (41/621)	0.046 (303/6641)	1.58	0.009
**HLA-DQB1*0501**	0.283 (276/974)	0.228 (3020/13273)	1.36	4.50E-05	0.292 (103/353)	0.227 (1508/6632)	1.39	0.0066	0.279 (173/621)	0.228 (1512/6641)	1.35	0.0017
**HLA-B*5601**	0.023 (22/974)	0.009 (113/13273)	2.55	9.30E-05	0.028 (10/353)	0.008 (56/6632)	3.5	0.00034	0.019 (12/621)	0.009 (57/6641)	1.92	0.048
**HLA-DQA1*0101**	0.328 (319/974)	0.27 (3588/13273)	1.32	9.60E-05	0.334 (118/353)	0.269 (1783/6632)	1.35	0.01	0.324 (201/621)	0.272 (1805/6641)	1.32	0.0024
**HLA-DPB1*1301**	0.061 (59/974)	0.033 (444/13273)	1.75	0.00011	0.051 (18/353)	0.035 (231/6632)	1.41	0.18	0.066 (41/621)	0.032 (213/6641)	2.03	8.30E-05
**HLA-DQB1*0502**	0.044 (43/974)	0.019 (251/13273)	1.94	0.00011	0.034 (12/353)	0.019 (123/6632)	1.61	0.13	0.05 (31/621)	0.019 (128/6641)	2.16	0.00027
**HLA-B*3501**	0.139 (135/974)	0.101 (1342/13273)	1.45	0.00013	0.11 (39/353)	0.1 (662/6632)	1.13	0.5	0.155 (96/621)	0.102 (680/6641)	1.65	2.90E-05
**HLA-DRB1*0402**	0.033 (32/974)	0.009 (123/13273)	2.2	2.00E-04	0.02 (7/353)	0.01 (65/6632)	1.61	0.24	0.04 (25/621)	0.009 (58/6641)	2.5	0.00049
**HLA-DQA1*0501**	0.495 (482/974)	0.421 (5583/13273)	1.28	0.00023	0.482 (170/353)	0.414 (2747/6632)	1.28	0.025	0.502 (312/621)	0.427 (2836/6641)	1.27	0.0058
**HLA-C*0401**	0.237 (231/974)	0.175 (2326/13273)	1.34	0.00023	0.193 (68/353)	0.175 (1161/6632)	1.06	0.7	0.262 (163/621)	0.175 (1165/6641)	1.54	1.10E-05
**HLA-DQB1*0603**	0.079 (77/974)	0.114 (1515/13273)	0.64	0.00028	0.068 (24/353)	0.113 (752/6632)	0.56	0.0073	0.085 (53/621)	0.115 (763/6641)	0.68	0.011
**HLA-DRB5*0202**	0.037 (36/974)	0.016 (210/13273)	1.97	3.00E-04	0.025 (9/353)	0.016 (103/6632)	1.42	0.33	0.043 (27/621)	0.016 (107/6641)	2.36	0.00014
**HLA-C*0602**	0.123 (120/974)	0.167 (2219/13273)	0.7	0.00034	0.122 (43/353)	0.165 (1091/6632)	0.7	0.03	0.124 (77/621)	0.17 (1128/6641)	0.7	0.0051
**HLA-DRB3*0202**	0.37 (360/974)	0.288 (3827/13273)	1.28	0.00058	0.314 (111/353)	0.282 (1873/6632)	1.08	0.53	0.401 (249/621)	0.294 (1954/6641)	1.38	3.00E-04
**HLA-DQA1*0103**	0.089 (87/974)	0.12 (1588/13273)	0.67	0.00062	0.085 (30/353)	0.119 (787/6632)	0.67	0.039	0.092 (57/621)	0.121 (801/6641)	0.67	0.006
**HLA-DRB1*1301**	0.076 (74/974)	0.11 (1455/13273)	0.66	0.00085	0.071 (25/353)	0.109 (721/6632)	0.62	0.026	0.079 (49/621)	0.111 (734/6641)	0.68	0.013
**HLA-DRB1*1601**	0.034 (33/974)	0.015 (201/13273)	1.88	0.0011	0.023 (8/353)	0.015 (99/6632)	1.31	0.47	0.04 (25/621)	0.015 (102/6641)	2.24	0.00055
**HLA-C*1604**	0.006 (6/974)	0.001 (11/13273)	5.34	0.0016	0.003 (1/353)	0.001 (4/6632)	4.37	0.2	0.008 (5/621)	0.001 (7/6641)	5.01	0.0092
**HLA-B*4901**	0.011 (11/974)	0.026 (349/13273)	0.38	0.0018	0.008 (3/353)	0.026 (174/6632)	0.29	0.035	0.013 (8/621)	0.026 (175/6641)	0.42	0.02
**HLA-DQB1*0303**	0.064 (62/974)	0.098 (1295/13273)	0.66	0.002	0.074 (26/353)	0.095 (632/6632)	0.77	0.21	0.058 (36/621)	0.1 (663/6641)	0.59	0.0033
**HLA-B*1401**	0.009 (9/974)	0.026 (340/13273)	0.37	0.0032	0.008 (3/353)	0.028 (183/6632)	0.31	0.043	0.01 (6/621)	0.024 (157/6641)	0.41	0.035
**HLA-DRB1*0804**	0.008 (8/974)	0.002 (26/13273)	3.34	0.0037	0.006 (2/353)	0.002 (15/6632)	2.19	0.3	0.01 (6/621)	0.002 (11/6641)	4.48	0.0045
**HLA-A*2601**	0.077 (75/974)	0.045 (601/13273)	1.46	0.0038	0.062 (22/353)	0.044 (294/6632)	1.29	0.28	0.085 (53/621)	0.046 (307/6641)	1.53	0.0088
**HLA-DPB1*0201**	0.208 (203/974)	0.237 (3152/13273)	0.79	0.0049	0.193 (68/353)	0.234 (1555/6632)	0.76	0.05	0.217 (135/621)	0.24 (1597/6641)	0.81	0.041
**HLA-DQB1*0302**	0.231 (225/974)	0.198 (2625/13273)	1.24	0.0064	0.207 (73/353)	0.199 (1317/6632)	1.06	0.66	0.245 (152/621)	0.197 (1308/6641)	1.34	0.003
**HLA-B*1801**	0.112 (109/974)	0.079 (1054/13273)	1.33	0.0075	0.125 (44/353)	0.081 (534/6632)	1.57	0.0075	0.105 (65/621)	0.078 (520/6641)	1.22	0.16
**HLA-DPB1*1401**	0.043 (42/974)	0.027 (360/13273)	1.54	0.01	0.028 (10/353)	0.028 (189/6632)	0.93	0.83	0.052 (32/621)	0.026 (171/6641)	2.03	0.00043
**HLA-DPB1*1501**	0.005 (5/974)	0.015 (197/13273)	0.31	0.01	0.003 (1/353)	0.016 (107/6632)	0.16	0.07	0.006 (4/621)	0.014 (90/6641)	0.43	0.097
**HLA-DPB1*0301**	0.236 (230/974)	0.205 (2718/13273)	1.22	0.011	0.21 (74/353)	0.205 (1361/6632)	1.03	0.82	0.251 (156/621)	0.204 (1357/6641)	1.34	0.0033
**HLA-DQB1*0604**	0.043 (42/974)	0.063 (833/13273)	0.66	0.011	0.034 (12/353)	0.06 (395/6632)	0.56	0.052	0.048 (30/621)	0.066 (438/6641)	0.7	0.067
**HLA-DRB1*1201**	0.017 (17/974)	0.032 (421/13273)	0.54	0.013	0.023 (8/353)	0.032 (210/6632)	0.71	0.35	0.014 (9/621)	0.032 (211/6641)	0.44	0.017
**HLA-C*1203**	0.11 (107/974)	0.073 (973/13273)	1.31	0.016	0.096 (34/353)	0.071 (469/6632)	1.29	0.18	0.118 (73/621)	0.076 (504/6641)	1.28	0.076
**HLA-B*1302**	0.024 (23/974)	0.038 (502/13273)	0.59	0.016	0.02 (7/353)	0.036 (239/6632)	0.53	0.1	0.026 (16/621)	0.04 (263/6641)	0.63	0.075
**HLA-DRB3*0301**	0.065 (63/974)	0.085 (1133/13273)	0.73	0.019	0.057 (20/353)	0.082 (543/6632)	0.68	0.096	0.069 (43/621)	0.089 (590/6641)	0.74	0.075
**HLA-C*1402**	0.031 (30/974)	0.018 (241/13273)	1.58	0.021	0.023 (8/353)	0.016 (103/6632)	1.39	0.37	0.035 (22/621)	0.021 (138/6641)	1.56	0.062
**HLA-DRB1*1101**	0.132 (129/974)	0.097 (1292/13273)	1.26	0.023	0.122 (43/353)	0.094 (626/6632)	1.23	0.22	0.138 (86/621)	0.1 (666/6641)	1.25	0.082

**HLA-DQB1*0301**	0.386 (376/974)	0.334 (4434/13273)	1.17	0.023	0.371 (131/353)	0.337 (2232/6632)	1.13	0.3	0.395 (245/621)	0.332 (2202/6641)	1.2	0.04
**HLA-DRB1*1302**	0.067 (65/974)	0.087 (1150/13273)	0.74	0.025	0.057 (20/353)	0.083 (549/6632)	0.67	0.087	0.072 (45/621)	0.09 (601/6641)	0.77	0.1
**HLA-DRB3*9901**	0.828 (806/974)	0.863 (11459/13273)	0.82	0.029	0.841 (297/353)	0.865 (5736/6632)	0.87	0.36	0.82 (509/621)	0.862 (5723/6641)	0.8	0.051
**HLA-A*2402**	0.192 (187/974)	0.158 (2092/13273)	1.21	0.029	0.19 (67/353)	0.158 (1045/6632)	1.22	0.16	0.193 (120/621)	0.158 (1047/6641)	1.18	0.13
**HLA-DRB1*0401**	0.163 (159/974)	0.206 (2731/13273)	0.82	0.031	0.184 (65/353)	0.209 (1383/6632)	0.9	0.47	0.151 (94/621)	0.203 (1348/6641)	0.78	0.034
**HLA-DPB1*0402**	0.235 (229/974)	0.208 (2760/13273)	1.18	0.037	0.227 (80/353)	0.211 (1399/6632)	1.11	0.44	0.24 (149/621)	0.205 (1361/6641)	1.22	0.048
**HLA-B*3701**	0.016 (16/974)	0.028 (376/13273)	0.6	0.044	0.02 (7/353)	0.027 (181/6632)	0.73	0.41	0.014 (9/621)	0.029 (195/6641)	0.52	0.055
**HLA-DPA1*0104**	0.005 (5/974)	0.011 (143/13273)	0.42	0.06	0.003 (1/353)	0.012 (80/6632)	0.21	0.13	0.006 (4/621)	0.009 (63/6641)	0.59	0.31
**HLA-C*0802**	0.067 (65/974)	0.079 (1047/13273)	0.78	0.062	0.054 (19/353)	0.084 (555/6632)	0.59	0.029	0.074 (46/621)	0.074 (492/6641)	0.94	0.68
**HLA-B*5701**	0.055 (54/974)	0.072 (962/13273)	0.76	0.063	0.048 (17/353)	0.071 (470/6632)	0.67	0.11	0.06 (37/621)	0.074 (492/6641)	0.81	0.23
**HLA-DRB1*1103**	0.012 (12/974)	0.007 (87/13273)	1.78	0.066	0.011 (4/353)	0.007 (45/6632)	1.61	0.36	0.013 (8/621)	0.006 (42/6641)	1.8	0.14
**HLA-B*3503**	0.034 (33/974)	0.023 (301/13273)	1.41	0.066	0.04 (14/353)	0.022 (146/6632)	1.73	0.056	0.031 (19/621)	0.023 (155/6641)	1.27	0.34
**HLA-C*1505**	0.012 (12/974)	0.004 (58/13273)	1.82	0.068	0.014 (5/353)	0.004 (28/6632)	2.65	0.05	0.011 (7/621)	0.005 (30/6641)	1.45	0.4
**HLA-C*0501**	0.174 (169/974)	0.205 (2727/13273)	0.85	0.068	0.207 (73/353)	0.204 (1355/6632)	1.05	0.73	0.155 (96/621)	0.207 (1372/6641)	0.74	0.0094
**HLA-B*3502**	0.034 (33/974)	0.014 (184/13273)	1.44	0.071	0.023 (8/353)	0.015 (99/6632)	1.09	0.83	0.04 (25/621)	0.013 (85/6641)	1.78	0.02
**HLA-C*0304**	0.16 (156/974)	0.154 (2039/13273)	1.17	0.088	0.161 (57/353)	0.152 (1009/6632)	1.14	0.39	0.159 (99/621)	0.155 (1030/6641)	1.17	0.19
**HLA-B*4001**	0.117 (114/974)	0.109 (1441/13273)	1.19	0.088	0.133 (47/353)	0.107 (710/6632)	1.35	0.068	0.108 (67/621)	0.11 (731/6641)	1.08	0.56
**HLA-B*0801**	0.253 (246/974)	0.244 (3236/13273)	1.14	0.092	0.261 (92/353)	0.243 (1611/6632)	1.16	0.24	0.248 (154/621)	0.245 (1625/6641)	1.14	0.2
**HLA-DPB1*1001**	0.045 (44/974)	0.034 (453/13273)	1.31	0.099	0.059 (21/353)	0.034 (223/6632)	1.77	0.016	0.037 (23/621)	0.035 (230/6641)	1.08	0.74
**HLA-DRB4*0103**	0.379 (369/974)	0.416 (5525/13273)	0.89	0.1	0.388 (137/353)	0.416 (2761/6632)	0.92	0.44	0.374 (232/621)	0.416 (2764/6641)	0.88	0.14
**HLA-C*0102**	0.082 (80/974)	0.068 (907/13273)	1.22	0.1	0.085 (30/353)	0.063 (419/6632)	1.38	0.1	0.081 (50/621)	0.073 (488/6641)	1.09	0.59
**HLA-B*3508**	0.017 (17/974)	0.009 (113/13273)	1.53	0.11	0.011 (4/353)	0.008 (56/6632)	1.13	0.81	0.021 (13/621)	0.009 (57/6641)	1.71	0.1
**HLA-B*5001**	0.015 (15/974)	0.02 (266/13273)	0.66	0.12	0.023 (8/353)	0.02 (134/6632)	0.99	0.98	0.011 (7/621)	0.02 (132/6641)	0.49	0.07
**HLA-A*6901**	0.001 (1/974)	0.002 (33/13273)	0.22	0.13	0 (0/353)	0.002 (11/6632)	NA	0.96	0.002 (1/621)	0.003 (22/6641)	0.21	0.14
**HLA-B*4405**	0.007 (7/974)	0.003 (43/13273)	1.81	0.15	0.006 (2/353)	0.003 (18/6632)	1.69	0.49	0.008 (5/621)	0.004 (25/6641)	1.74	0.28
**HLA-DRB1*1303**	0.014 (14/974)	0.019 (254/13273)	0.68	0.17	0.014 (5/353)	0.02 (131/6632)	0.65	0.35	0.014 (9/621)	0.019 (123/6641)	0.68	0.28
**HLA-B*5801**	0.018 (18/974)	0.011 (144/13273)	1.4	0.19	0.02 (7/353)	0.011 (76/6632)	1.52	0.3	0.018 (11/621)	0.01 (68/6641)	1.38	0.34
**HLA-B*1518**	0.003 (3/974)	0.006 (85/13273)	0.46	0.19	0.003 (1/353)	0.005 (35/6632)	0.53	0.53	0.003 (2/621)	0.008 (50/6641)	0.41	0.22
**HLA-A*0302**	0.009 (9/974)	0.004 (53/13273)	1.62	0.19	0.006 (2/353)	0.003 (18/6632)	1.65	0.51	0.011 (7/621)	0.005 (35/6641)	1.34	0.49
**HLA-DRB1*0803**	0.006 (6/974)	0.004 (48/13273)	1.76	0.2	0.006 (2/353)	0.004 (26/6632)	1.56	0.55	0.006 (4/621)	0.003 (22/6641)	2	0.21
**HLA-A*3001**	0.017 (17/974)	0.022 (290/13273)	0.72	0.2	0.02 (7/353)	0.02 (132/6632)	0.9	0.79	0.016 (10/621)	0.024 (158/6641)	0.63	0.17
**HLA-DPB1*0202**	0.01 (10/974)	0.014 (192/13273)	0.66	0.21	0.006 (2/353)	0.012 (77/6632)	0.48	0.31	0.013 (8/621)	0.017 (115/6641)	0.67	0.28
**HLA-DPB1*1601**	0.007 (7/974)	0.012 (163/13273)	0.62	0.21	0.008 (3/353)	0.013 (85/6632)	0.67	0.5	0.006 (4/621)	0.012 (78/6641)	0.6	0.33
**HLA-DPB1*0901**	0.018 (18/974)	0.013 (179/13273)	1.36	0.22	0.025 (9/353)	0.014 (91/6632)	1.82	0.092	0.014 (9/621)	0.013 (88/6641)	1.13	0.72
**HLA-DRB5*9901**	0.973 (948/974)	0.978 (12976/13273)	0.77	0.22	0.98 (346/353)	0.979 (6490/6632)	1.01	0.98	0.969 (602/621)	0.977 (6486/6641)	0.67	0.11
**HLA-B*5108**	0.003 (3/974)	0.001 (14/13273)	2.2	0.23	0.003 (1/353)	0.001 (8/6632)	1.82	0.57	0.003 (2/621)	0.001 (6/6641)	3.87	0.1
**HLA-B*4101**	0.006 (6/974)	0.007 (90/13273)	0.6	0.23	0.008 (3/353)	0.006 (42/6632)	1.02	0.98	0.005 (3/621)	0.007 (48/6641)	0.43	0.16
**HLA-DRB1*0103**	0.023 (22/974)	0.031 (407/13273)	0.78	0.26	0.031 (11/353)	0.03 (197/6632)	1.07	0.84	0.018 (11/621)	0.032 (210/6641)	0.6	0.11
**HLA-DPA1*0202**	0.05 (49/974)	0.061 (816/13273)	0.84	0.26	0.045 (16/353)	0.064 (425/6632)	0.7	0.17	0.053 (33/621)	0.059 (391/6641)	0.95	0.8
**HLA-A*3002**	0.018 (18/974)	0.021 (282/13273)	0.76	0.27	0.011 (4/353)	0.018 (118/6632)	0.59	0.31	0.023 (14/621)	0.025 (164/6641)	0.79	0.4
**HLA-B*2705**	0.089 (87/974)	0.085 (1124/13273)	1.13	0.28	0.076 (27/353)	0.085 (564/6632)	0.92	0.67	0.097 (60/621)	0.084 (560/6641)	1.28	0.092
**HLA-B*4501**	0.011 (11/974)	0.015 (197/13273)	0.71	0.28	0.011 (4/353)	0.015 (102/6632)	0.7	0.49	0.011 (7/621)	0.014 (95/6641)	0.73	0.43
**HLA-A*3303**	0.007 (7/974)	0.004 (54/13273)	1.54	0.29	0.006 (2/353)	0.004 (29/6632)	1.23	0.78	0.008 (5/621)	0.004 (25/6641)	1.77	0.26
**HLA-A*3201**	0.061 (59/974)	0.07 (933/13273)	0.86	0.29	0.059 (21/353)	0.073 (486/6632)	0.8	0.34	0.061 (38/621)	0.067 (447/6641)	0.93	0.66
**HLA-B*5501**	0.029 (28/974)	0.035 (460/13273)	0.81	0.29	0.031 (11/353)	0.037 (244/6632)	0.85	0.6	0.027 (17/621)	0.033 (216/6641)	0.78	0.34
**HLA-DRB1*1401**	0.056 (55/974)	0.048 (635/13273)	1.17	0.29	0.048 (17/353)	0.046 (305/6632)	1.02	0.92	0.061 (38/621)	0.05 (330/6641)	1.22	0.26
**HLA-DQA1*0601**	0.006 (6/974)	0.004 (53/13273)	1.58	0.29	0.006 (2/353)	0.005 (30/6632)	1.31	0.71	0.006 (4/621)	0.003 (23/6641)	1.98	0.22
**HLA-A*2501**	0.046 (45/974)	0.04 (527/13273)	1.18	0.3	0.054 (19/353)	0.04 (268/6632)	1.36	0.21	0.042 (26/621)	0.039 (259/6641)	1.11	0.62
**HLA-DRB1*0301**	0.259 (252/974)	0.254 (3376/13273)	1.08	0.3	0.278 (98/353)	0.251 (1667/6632)	1.19	0.16	0.248 (154/621)	0.257 (1709/6641)	1.03	0.77
**HLA-C*0701**	0.32 (312/974)	0.309 (4100/13273)	1.08	0.31	0.348 (123/353)	0.307 (2034/6632)	1.22	0.08	0.304 (189/621)	0.311 (2066/6641)	1	0.99
**HLA-DRB1*0901**	0.018 (18/974)	0.025 (331/13273)	0.78	0.32	0.023 (8/353)	0.024 (157/6632)	1	1	0.016 (10/621)	0.026 (174/6641)	0.65	0.19
**HLA-DRB1*0407**	0.027 (26/974)	0.023 (302/13273)	1.23	0.32	0.034 (12/353)	0.023 (154/6632)	1.55	0.15	0.023 (14/621)	0.022 (148/6641)	1.08	0.78
**HLA-DRB1*1602**	0.004 (4/974)	0.002 (25/13273)	1.72	0.32	0.003 (1/353)	0.002 (12/6632)	1.28	0.81	0.005 (3/621)	0.002 (13/6641)	2.34	0.19
**HLA-DQB1*0201**	0.261 (254/974)	0.257 (3409/13273)	1.08	0.33	0.286 (101/353)	0.253 (1678/6632)	1.22	0.1	0.246 (153/621)	0.261 (1731/6641)	1	0.99
**HLA-DPB1*0601**	0.039 (38/974)	0.035 (458/13273)	1.18	0.33	0.037 (13/353)	0.032 (214/6632)	1.18	0.57	0.04 (25/621)	0.037 (244/6641)	1.13	0.57

**HLA-DRB1*0403**	0.016 (16/974)	0.011 (144/13273)	1.29	0.35	0.011 (4/353)	0.012 (78/6632)	0.92	0.87	0.019 (12/621)	0.01 (66/6641)	1.53	0.2
**HLA-DQB1*0503**	0.057 (56/974)	0.05 (662/13273)	1.14	0.38	0.051 (18/353)	0.048 (319/6632)	1.03	0.91	0.061 (38/621)	0.052 (343/6641)	1.19	0.33
**HLA-B*3906**	0.016 (16/974)	0.014 (188/13273)	1.26	0.38	0.014 (5/353)	0.014 (96/6632)	0.99	0.98	0.018 (11/621)	0.014 (92/6641)	1.42	0.28
**HLA-B*4002**	0.025 (24/974)	0.021 (278/13273)	1.21	0.39	0.017 (6/353)	0.021 (138/6632)	0.83	0.65	0.029 (18/621)	0.021 (140/6641)	1.5	0.12
**HLA-DRB3*0101**	0.271 (264/974)	0.296 (3924/13273)	0.94	0.39	0.275 (97/353)	0.292 (1939/6632)	0.95	0.69	0.269 (167/621)	0.299 (1985/6641)	0.92	0.4
**HLA-DQB1*0602**	0.219 (213/974)	0.246 (3271/13273)	0.93	0.39	0.229 (81/353)	0.248 (1643/6632)	0.96	0.75	0.213 (132/621)	0.245 (1628/6641)	0.91	0.36
**HLA-C*1701**	0.016 (16/974)	0.015 (204/13273)	0.8	0.4	0.011 (4/353)	0.015 (99/6632)	0.65	0.41	0.019 (12/621)	0.016 (105/6641)	0.83	0.55
**HLA-C*0303**	0.092 (90/974)	0.106 (1404/13273)	0.91	0.4	0.099 (35/353)	0.109 (722/6632)	0.94	0.72	0.089 (55/621)	0.103 (682/6641)	0.9	0.48
**HLA-DPB1*1701**	0.025 (24/974)	0.025 (332/13273)	0.83	0.4	0.028 (10/353)	0.023 (155/6632)	1.1	0.78	0.023 (14/621)	0.027 (177/6641)	0.69	0.19
**HLA-B*3901**	0.027 (26/974)	0.022 (296/13273)	1.18	0.42	0.02 (7/353)	0.022 (143/6632)	0.92	0.83	0.031 (19/621)	0.023 (153/6641)	1.26	0.36
**HLA-DQB1*0504**	0.001 (1/974)	0.003 (34/13273)	0.44	0.42	0 (0/353)	0.003 (17/6632)	NA	0.96	0.002 (1/621)	0.003 (17/6641)	0.72	0.75
**HLA-DRB1*0404**	0.092 (90/974)	0.089 (1183/13273)	1.1	0.43	0.082 (29/353)	0.088 (586/6632)	0.94	0.75	0.098 (61/621)	0.09 (597/6641)	1.2	0.21
**HLA-DQA1*0102**	0.319 (311/974)	0.341 (4527/13273)	0.94	0.43	0.317 (112/353)	0.339 (2246/6632)	0.94	0.59	0.32 (199/621)	0.343 (2281/6641)	0.94	0.47
**HLA-B*4102**	0.009 (9/974)	0.009 (126/13273)	0.76	0.44	0.003 (1/353)	0.009 (62/6632)	0.28	0.21	0.013 (8/621)	0.01 (64/6641)	0.9	0.79
**HLA-C*0702**	0.263 (256/974)	0.27 (3581/13273)	1.06	0.46	0.297 (105/353)	0.272 (1803/6632)	1.19	0.14	0.243 (151/621)	0.268 (1778/6641)	0.98	0.86
**HLA-C*0302**	0.005 (5/974)	0.003 (45/13273)	1.42	0.46	0.003 (1/353)	0.004 (24/6632)	0.71	0.73	0.006 (4/621)	0.003 (21/6641)	1.87	0.26
**HLA-DPB1*0101**	0.101 (98/974)	0.113 (1502/13273)	0.92	0.47	0.096 (34/353)	0.117 (773/6632)	0.83	0.31	0.103 (64/621)	0.11 (729/6641)	1.01	0.93
**HLA-A*0203**	0.002 (2/974)	0.001 (17/13273)	1.71	0.47	0.003 (1/353)	0.002 (10/6632)	1.96	0.52	0.002 (1/621)	0.001 (7/6641)	1.6	0.66
**HLA-DPA1*0301**	0.002 (2/974)	0.001 (10/13273)	1.76	0.47	0 (0/353)	0.001 (4/6632)	NA	0.97	0.003 (2/621)	0.001 (6/6641)	2.36	0.32
**HLA-DPA1*0201**	0.292 (284/974)	0.275 (3652/13273)	1.05	0.48	0.295 (104/353)	0.278 (1842/6632)	1.05	0.66	0.29 (180/621)	0.273 (1810/6641)	1.09	0.37
**HLA-A*3402**	0.001 (1/974)	0.002 (26/13273)	0.49	0.49	0 (0/353)	0.002 (14/6632)	NA	0.96	0.002 (1/621)	0.002 (12/6641)	0.94	0.95
**HLA-A*2301**	0.03 (29/974)	0.032 (423/13273)	0.87	0.49	0.042 (15/353)	0.032 (215/6632)	1.27	0.39	0.023 (14/621)	0.031 (208/6641)	0.67	0.15
**HLA-DPB1*0401**	0.664 (647/974)	0.68 (9031/13273)	0.95	0.49	0.691 (244/353)	0.677 (4491/6632)	1.08	0.49	0.649 (403/621)	0.684 (4540/6641)	0.88	0.14
**HLA-A*6802**	0.012 (12/974)	0.013 (172/13273)	0.81	0.49	0.011 (4/353)	0.013 (85/6632)	0.82	0.69	0.013 (8/621)	0.013 (87/6641)	0.8	0.55
**HLA-B*0705**	0.008 (8/974)	0.004 (56/13273)	1.29	0.51	0.003 (1/353)	0.004 (28/6632)	0.54	0.54	0.011 (7/621)	0.004 (28/6641)	1.64	0.26
**HLA-DPA1*0103**	0.959 (934/974)	0.964 (12798/13273)	0.9	0.53	0.958 (338/353)	0.961 (6375/6632)	0.97	0.92	0.96 (596/621)	0.967 (6423/6641)	0.81	0.33
**HLA-DRB1*1001**	0.018 (18/974)	0.014 (187/13273)	1.17	0.54	0.014 (5/353)	0.012 (82/6632)	1.08	0.87	0.021 (13/621)	0.016 (105/6641)	1.14	0.67
**HLA-A*6601**	0.009 (9/974)	0.007 (88/13273)	1.24	0.55	0.008 (3/353)	0.007 (45/6632)	1.24	0.72	0.01 (6/621)	0.006 (43/6641)	1.15	0.76
**HLA-B*1517**	0.005 (5/974)	0.005 (62/13273)	0.76	0.56	0.008 (3/353)	0.004 (25/6632)	1.81	0.34	0.003 (2/621)	0.006 (37/6641)	0.38	0.19
**HLA-DRB1*1501**	0.225 (219/974)	0.248 (3292/13273)	0.96	0.57	0.238 (84/353)	0.25 (1657/6632)	0.99	0.95	0.217 (135/621)	0.246 (1635/6641)	0.93	0.45
**HLA-DRB1*1502**	0.011 (11/974)	0.01 (131/13273)	0.84	0.58	0.017 (6/353)	0.011 (72/6632)	1.37	0.46	0.008 (5/621)	0.009 (59/6641)	0.57	0.24
**HLA-DQB1*0601**	0.011 (11/974)	0.01 (130/13273)	0.84	0.6	0.017 (6/353)	0.01 (69/6632)	1.43	0.41	0.008 (5/621)	0.009 (61/6641)	0.56	0.22
**HLA-A*3101**	0.048 (47/974)	0.052 (694/13273)	0.92	0.6	0.042 (15/353)	0.051 (341/6632)	0.81	0.43	0.052 (32/621)	0.053 (353/6641)	0.96	0.84
**HLA-A*0301**	0.257 (250/974)	0.26 (3456/13273)	1.04	0.6	0.258 (91/353)	0.259 (1718/6632)	1.04	0.76	0.256 (159/621)	0.262 (1738/6641)	1.05	0.63
**HLA-B*5703**	0.002 (2/974)	0.001 (11/13273)	1.48	0.62	0 (0/353)	0.001 (5/6632)	NA	0.96	0.003 (2/621)	0.001 (6/6641)	2.02	0.4
**HLA-DRB1*0102**	0.028 (27/974)	0.023 (306/13273)	0.9	0.63	0.02 (7/353)	0.024 (161/6632)	0.66	0.29	0.032 (20/621)	0.022 (145/6641)	1.14	0.61
**HLA-B*4402**	0.187 (182/974)	0.205 (2721/13273)	0.96	0.64	0.218 (77/353)	0.205 (1361/6632)	1.13	0.36	0.169 (105/621)	0.205 (1360/6641)	0.86	0.19
**HLA-B*1516**	0.001 (1/974)	0.001 (17/13273)	0.62	0.65	0 (0/353)	0.001 (6/6632)	NA	0.96	0.002 (1/621)	0.002 (11/6641)	0.8	0.83
**HLA-B*5301**	0.009 (9/974)	0.006 (81/13273)	1.17	0.66	0.006 (2/353)	0.007 (49/6632)	0.66	0.57	0.011 (7/621)	0.005 (32/6641)	1.78	0.18
**HLA-B*1402**	0.06 (58/974)	0.057 (756/13273)	0.94	0.66	0.045 (16/353)	0.06 (397/6632)	0.68	0.15	0.068 (42/621)	0.054 (359/6641)	1.16	0.4
**HLA-DQB1*0609**	0.021 (20/974)	0.023 (302/13273)	0.9	0.67	0.023 (8/353)	0.022 (147/6632)	1.02	0.96	0.019 (12/621)	0.023 (155/6641)	0.84	0.56
**HLA-DPB1*1901**	0.008 (8/974)	0.011 (140/13273)	0.85	0.67	0.006 (2/353)	0.009 (61/6632)	0.62	0.51	0.01 (6/621)	0.012 (79/6641)	0.92	0.84
**HLA-A*3004**	0.004 (4/974)	0.003 (36/13273)	1.24	0.69	0.006 (2/353)	0.004 (24/6632)	1.38	0.66	0.003 (2/621)	0.002 (12/6641)	1.37	0.68
**HLA-C*1602**	0.004 (4/974)	0.004 (57/13273)	0.81	0.69	0.006 (2/353)	0.004 (26/6632)	1.24	0.77	0.003 (2/621)	0.005 (31/6641)	0.66	0.57
**HLA-DPB1*2001**	0.006 (6/974)	0.006 (76/13273)	1.18	0.69	0.008 (3/353)	0.006 (41/6632)	1.44	0.55	0.005 (3/621)	0.005 (35/6641)	1.02	0.98
**HLA-DPA1*0105**	0.001 (1/974)	0.002 (20/13273)	0.68	0.71	0 (0/353)	0.001 (9/6632)	NA	0.97	0.002 (1/621)	0.002 (11/6641)	1.03	0.98
**HLA-DRB5*0101**	0.228 (222/974)	0.248 (3292/13273)	0.97	0.72	0.238 (84/353)	0.25 (1655/6632)	0.99	0.96	0.222 (138/621)	0.246 (1637/6641)	0.95	0.6
**HLA-B*2702**	0.004 (4/974)	0.003 (36/13273)	1.21	0.72	0 (0/353)	0.003 (19/6632)	NA	0.97	0.006 (4/621)	0.003 (17/6641)	1.9	0.28
**HLA-A*6801**	0.063 (61/974)	0.063 (830/13273)	1.05	0.72	0.054 (19/353)	0.063 (416/6632)	0.87	0.58	0.068 (42/621)	0.062 (414/6641)	1.16	0.39
**HLA-C*1202**	0.016 (16/974)	0.011 (150/13273)	1.1	0.73	0.02 (7/353)	0.012 (78/6632)	1.48	0.33	0.014 (9/621)	0.011 (72/6641)	0.93	0.84
**HLA-A*0205**	0.022 (21/974)	0.019 (256/13273)	0.92	0.73	0.02 (7/353)	0.019 (124/6632)	0.95	0.89	0.023 (14/621)	0.02 (132/6641)	0.89	0.7
**HLA-C*1502**	0.039 (38/974)	0.035 (470/13273)	1.06	0.74	0.037 (13/353)	0.037 (244/6632)	0.99	0.96	0.04 (25/621)	0.034 (226/6641)	1.13	0.56
**HLA-A*0201**	0.464 (452/974)	0.485 (6438/13273)	0.98	0.75	0.47 (166/353)	0.487 (3229/6632)	0.97	0.76	0.461 (286/621)	0.483 (3209/6641)	0.99	0.91
**HLA-A*2901**	0.008 (8/974)	0.004 (55/13273)	1.13	0.75	0.003 (1/353)	0.004 (26/6632)	0.57	0.58	0.011 (7/621)	0.004 (29/6641)	1.34	0.51
**HLA-B*5101**	0.09 (88/974)	0.083 (1096/13273)	1.04	0.75	0.074 (26/353)	0.081 (534/6632)	0.87	0.52	0.1 (62/621)	0.085 (562/6641)	1.12	0.43

**HLA-B*5201**	0.016 (16/974)	0.011 (152/13273)	1.08	0.77	0.02 (7/353)	0.012 (80/6632)	1.43	0.37	0.014 (9/621)	0.011 (72/6641)	0.93	0.83
**HLA-DRB1*0802**	0.001 (1/974)	0.002 (20/13273)	0.74	0.77	0 (0/353)	0.002 (10/6632)	NA	0.97	0.002 (1/621)	0.002 (10/6641)	1.08	0.94
**HLA-DRB1*1102**	0.008 (8/974)	0.006 (83/13273)	1.11	0.78	0.003 (1/353)	0.006 (42/6632)	0.41	0.38	0.011 (7/621)	0.006 (41/6641)	1.45	0.38
**HLA-B*1524**	0.001 (1/974)	0.001 (12/13273)	1.33	0.79	0 (0/353)	0.001 (5/6632)	NA	0.96	0.002 (1/621)	0.001 (7/6641)	1.75	0.6
**HLA-B*4006**	0.001 (1/974)	0.001 (10/13273)	1.32	0.79	0.003 (1/353)	0.001 (5/6632)	4.05	0.2	0 (0/621)	0.001 (5/6641)	NA	0.96
**HLA-DPB1*0501**	0.043 (42/974)	0.044 (580/13273)	1.04	0.79	0.054 (19/353)	0.045 (299/6632)	1.23	0.39	0.037 (23/621)	0.042 (281/6641)	0.97	0.91
**HLA-DRB1*1404**	0.002 (2/974)	0.002 (29/13273)	0.83	0.81	0.003 (1/353)	0.002 (13/6632)	1.23	0.84	0.002 (1/621)	0.002 (16/6641)	0.61	0.64
**HLA-DPB1*2301**	0.003 (3/974)	0.004 (49/13273)	0.86	0.81	0.003 (1/353)	0.003 (19/6632)	1.03	0.97	0.003 (2/621)	0.005 (30/6641)	0.73	0.67
**HLA-C*0801**	0.001 (1/974)	0.002 (20/13273)	0.79	0.82	0 (0/353)	0.002 (12/6632)	NA	0.96	0.002 (1/621)	0.001 (8/6641)	1.37	0.77
**HLA-A*0101**	0.311 (303/974)	0.323 (4282/13273)	0.98	0.82	0.351 (124/353)	0.323 (2139/6632)	1.16	0.21	0.288 (179/621)	0.323 (2143/6641)	0.9	0.28
**HLA-DQB1*0304**	0.003 (3/974)	0.003 (43/13273)	0.87	0.82	0.003 (1/353)	0.003 (20/6632)	1.05	0.96	0.003 (2/621)	0.003 (23/6641)	0.81	0.78
**HLA-A*0206**	0.003 (3/974)	0.003 (40/13273)	1.14	0.83	0 (0/353)	0.003 (20/6632)	NA	0.97	0.005 (3/621)	0.003 (20/6641)	2.01	0.26
**HLA-B*4801**	0.001 (1/974)	0.001 (19/13273)	0.81	0.84	0 (0/353)	0.002 (11/6632)	NA	0.97	0.002 (1/621)	0.001 (8/6641)	1.29	0.81
**HLA-A*1101**	0.116 (113/974)	0.118 (1570/13273)	0.98	0.85	0.096 (34/353)	0.116 (772/6632)	0.8	0.24	0.127 (79/621)	0.12 (798/6641)	1.06	0.66
**HLA-B*1501**	0.113 (110/974)	0.123 (1639/13273)	0.98	0.86	0.11 (39/353)	0.123 (816/6632)	0.93	0.67	0.114 (71/621)	0.124 (823/6641)	1	0.98
**HLA-DRB1*0405**	0.011 (11/974)	0.011 (140/13273)	0.95	0.88	0.017 (6/353)	0.011 (74/6632)	1.36	0.48	0.008 (5/621)	0.01 (66/6641)	0.74	0.52
**HLA-B*4701**	0.005 (5/974)	0.006 (79/13273)	0.93	0.88	0.003 (1/353)	0.006 (40/6632)	0.49	0.48	0.006 (4/621)	0.006 (39/6641)	1.23	0.69
**HLA-C*0202**	0.081 (79/974)	0.079 (1054/13273)	1.02	0.88	0.059 (21/353)	0.08 (532/6632)	0.72	0.16	0.093 (58/621)	0.079 (522/6641)	1.2	0.21
**HLA-A*0202**	0.002 (2/974)	0.002 (22/13273)	0.9	0.89	0 (0/353)	0.002 (10/6632)	NA	0.97	0.003 (2/621)	0.002 (12/6641)	1.17	0.85
**HLA-B*1503**	0 (0/974)	0.003 (37/13273)	NA	0.93	0 (0/353)	0.003 (17/6632)	NA	0.97	0 (0/621)	0.003 (20/6641)	NA	0.95
**HLA-DRB1*0408**	0.006 (6/974)	0.006 (86/13273)	0.97	0.94	0.006 (2/353)	0.007 (45/6632)	0.93	0.92	0.006 (4/621)	0.006 (41/6641)	1.03	0.96
**HLA-B*3801**	0.035 (34/974)	0.025 (328/13273)	0.99	0.94	0.025 (9/353)	0.023 (153/6632)	0.91	0.78	0.04 (25/621)	0.026 (175/6641)	0.96	0.84
**HLA-DRB1*1305**	0.003 (3/974)	0.001 (19/13273)	0.96	0.95	0 (0/353)	0.001 (7/6632)	NA	0.97	0.005 (3/621)	0.002 (12/6641)	0.98	0.97
**HLA-A*0102**	0 (0/974)	0.001 (10/13273)	NA	0.95	0 (0/353)	0.001 (5/6632)	NA	0.96	0 (0/621)	0.001 (5/6641)	NA	0.96
**HLA-DRB1*0806**	0 (0/974)	0.001 (10/13273)	NA	0.95	0 (0/353)	0.001 (4/6632)	NA	0.97	0 (0/621)	0.001 (6/6641)	NA	0.96
**HLA-DRB1*0406**	0 (0/974)	0.001 (10/13273)	NA	0.95	0 (0/353)	0.001 (5/6632)	NA	0.96	0 (0/621)	0.001 (5/6641)	NA	0.96
**HLA-B*4404**	0 (0/974)	0.002 (22/13273)	NA	0.95	0 (0/353)	0.002 (10/6632)	NA	0.97	0 (0/621)	0.002 (12/6641)	NA	0.96
**HLA-C*0704**	0.035 (34/974)	0.036 (477/13273)	0.99	0.95	0.028 (10/353)	0.037 (245/6632)	0.77	0.43	0.039 (24/621)	0.035 (232/6641)	1.14	0.55
**HLA-B*0702**	0.234 (228/974)	0.251 (3334/13273)	1	0.96	0.269 (95/353)	0.253 (1679/6632)	1.15	0.27	0.214 (133/621)	0.249 (1655/6641)	0.91	0.37
**HLA-A*3301**	0.017 (17/974)	0.014 (192/13273)	0.99	0.98	0.008 (3/353)	0.016 (104/6632)	0.46	0.18	0.023 (14/621)	0.013 (88/6641)	1.48	0.19
**HLA-DQA1*0301**	0.347 (338/974)	0.357 (4735/13273)	1	0.98	0.346 (122/353)	0.358 (2373/6632)	0.98	0.84	0.348 (216/621)	0.356 (2362/6641)	1.01	0.91

Only alleles seen in >=10 controls in the meta-analysis cohort are shown. P-values below the GWS threshold are coloured orange, those with suggestive significance ($P<5x10^{-6}$) are coloured yellow. Rare alleles for which odds ratios could not be accurately computed are assigned OR = NA. CO = control, OR = odds ratio, NA = not applicable.

Supplementary Table 5: Classical HLA allele frequencies and disease associations with dSSc in all three study cohorts.

	dSSC vs CONTROL											
	Meta-analysis Cohort				Cohort 1				Cohort 2			
HLA Allele	dSSc Prop.(Count)	CO Prop.(Count)	OR	p	dSSc Prop.(Count)	CO Prop.(Count)	OR	p	dSSc Prop.(Count)	CO Prop.(Count)	OR	p
HLA-DPB1*1301	0.108 (51/474)	0.033 (444/13273)	3.2	2.40E-13	0.109 (15/137)	0.035 (231/6632)	3.02	0.00013	0.107 (36/337)	0.032 (213/6641)	3.41	1.90E-10
HLA-DRB1*1104	0.131 (62/474)	0.035 (459/13273)	2.93	3.30E-12	0.168 (23/137)	0.035 (230/6632)	3.47	1.90E-06	0.116 (39/337)	0.034 (229/6641)	2.71	2.60E-07
HLA-DQA1*0501	0.536 (254/474)	0.421 (5583/13273)	1.48	3.60E-05	0.584 (80/137)	0.414 (2747/6632)	1.82	0.00072	0.516 (174/337)	0.427 (2836/6641)	1.34	0.0096
HLA-DQB1*0301	0.445 (211/474)	0.334 (4434/13273)	1.46	7.90E-05	0.467 (64/137)	0.337 (2232/6632)	1.53	0.015	0.436 (147/337)	0.332 (2202/6641)	1.44	0.0015
HLA-DQA1*0103	0.07 (33/474)	0.12 (1588/13273)	0.49	0.00011	0.044 (6/137)	0.119 (787/6632)	0.29	0.0036	0.08 (27/337)	0.121 (801/6641)	0.57	0.007
HLA-DRB3*0202	0.403 (191/474)	0.288 (3827/13273)	1.4	0.00061	0.438 (60/137)	0.282 (1873/6632)	1.57	0.013	0.389 (131/337)	0.294 (1954/6641)	1.31	0.021
HLA-DRB1*1301	0.061 (29/474)	0.11 (1455/13273)	0.52	0.00064	0.044 (6/137)	0.109 (721/6632)	0.37	0.018	0.068 (23/337)	0.111 (734/6641)	0.57	0.011
HLA-C*1203	0.139 (66/474)	0.073 (973/13273)	1.62	0.00066	0.161 (22/137)	0.071 (469/6632)	1.88	0.011	0.131 (44/337)	0.076 (504/6641)	1.48	0.024
HLA-DQB1*0603	0.07 (33/474)	0.114 (1515/13273)	0.54	0.00093	0.051 (7/137)	0.113 (752/6632)	0.39	0.016	0.077 (26/337)	0.115 (763/6641)	0.61	0.017
HLA-C*1502	0.068 (32/474)	0.035 (470/13273)	1.88	0.00094	0.066 (9/137)	0.037 (244/6632)	1.68	0.14	0.068 (23/337)	0.034 (226/6641)	1.97	0.003
HLA-DRB1*0804	0.013 (6/474)	0.002 (26/13273)	4.47	0.0014	0.015 (2/137)	0.002 (15/6632)	3.7	0.1	0.012 (4/337)	0.002 (11/6641)	5.64	0.0041
HLA-C*1602	0.017 (8/474)	0.004 (57/13273)	3.39	0.0017	0.007 (1/137)	0.004 (26/6632)	1.21	0.85	0.021 (7/337)	0.005 (31/6641)	4.19	9.00E-04
HLA-B*5108	0.008 (4/474)	0.001 (14/13273)	6.34	0.0017	0.007 (1/137)	0.001 (8/6632)	2.63	0.38	0.009 (3/337)	0.001 (6/6641)	10.64	0.001
HLA-B*1501	0.065 (31/474)	0.123 (1639/13273)	0.55	0.0018	0.051 (7/137)	0.123 (816/6632)	0.45	0.04	0.071 (24/337)	0.124 (823/6641)	0.59	0.016
HLA-B*1801	0.131 (62/474)	0.079 (1054/13273)	1.55	0.0021	0.168 (23/137)	0.081 (534/6632)	1.96	0.0047	0.116 (39/337)	0.078 (520/6641)	1.38	0.071
HLA-B*4006	0.006 (3/474)	0.001 (10/13273)	7.83	0.0022	0.015 (2/137)	0.001 (5/6632)	23.18	0.00032	0.003 (1/337)	0.001 (5/6641)	3.11	0.31
HLA-DQB1*0502	0.049 (23/474)	0.019 (251/13273)	1.96	0.0033	0.051 (7/137)	0.019 (123/6632)	1.92	0.11	0.047 (16/337)	0.019 (128/6641)	1.96	0.015
HLA-DRB1*1601	0.04 (19/474)	0.015 (201/13273)	2.06	0.0038	0.044 (6/137)	0.015 (99/6632)	2.06	0.1	0.039 (13/337)	0.015 (102/6641)	2.07	0.017
HLA-C*0501	0.141 (67/474)	0.205 (2727/13273)	0.68	0.0042	0.08 (11/137)	0.204 (1355/6632)	0.37	0.002	0.166 (56/337)	0.207 (1372/6641)	0.81	0.15
HLA-B*5601	0.023 (11/474)	0.009 (113/13273)	2.51	0.0046	0.015 (2/137)	0.008 (56/6632)	1.78	0.43	0.027 (9/337)	0.009 (57/6641)	2.67	0.0081
HLA-DRB5*0202	0.04 (19/474)	0.016 (210/13273)	1.97	0.0065	0.036 (5/137)	0.016 (103/6632)	1.6	0.33	0.042 (14/337)	0.016 (107/6641)	2.15	0.0098
HLA-B*3501	0.137 (65/474)	0.101 (1342/13273)	1.44	0.0078	0.146 (20/137)	0.1 (662/6632)	1.51	0.098	0.134 (45/337)	0.102 (680/6641)	1.42	0.036
HLA-A*6601	0.019 (9/474)	0.007 (88/13273)	2.5	0.011	0.007 (1/137)	0.007 (45/6632)	0.96	0.97	0.024 (8/337)	0.006 (43/6641)	3.24	0.0034
HLA-C*0401	0.241 (114/474)	0.175 (2326/13273)	1.32	0.012	0.255 (35/137)	0.175 (1161/6632)	1.31	0.18	0.234 (79/337)	0.175 (1165/6641)	1.36	0.023
HLA-DRB1*0401	0.139 (66/474)	0.206 (2731/13273)	0.71	0.012	0.161 (22/137)	0.209 (1383/6632)	0.87	0.56	0.131 (44/337)	0.203 (1348/6641)	0.66	0.013
HLA-DRB1*0701	0.205 (97/474)	0.257 (3406/13273)	0.75	0.014	0.175 (24/137)	0.262 (1737/6632)	0.6	0.026	0.217 (73/337)	0.251 (1669/6641)	0.83	0.18
HLA-DRB4*0101	0.143 (68/474)	0.188 (2491/13273)	0.72	0.015	0.102 (14/137)	0.194 (1289/6632)	0.47	0.0078	0.16 (54/337)	0.181 (1202/6641)	0.87	0.35
HLA-DQA1*0201	0.205 (97/474)	0.256 (3397/13273)	0.75	0.015	0.175 (24/137)	0.261 (1733/6632)	0.6	0.026	0.217 (73/337)	0.251 (1664/6641)	0.84	0.19
HLA-C*0701	0.357 (169/474)	0.309 (4100/13273)	1.27	0.016	0.401 (55/137)	0.307 (2034/6632)	1.58	0.01	0.338 (114/337)	0.311 (2066/6641)	1.15	0.23
HLA-C*1701	0.04 (19/474)	0.015 (204/13273)	1.83	0.018	0.036 (5/137)	0.015 (99/6632)	1.61	0.33	0.042 (14/337)	0.016 (105/6641)	1.95	0.027
HLA-B*5701	0.042 (20/474)	0.072 (962/13273)	0.58	0.019	0.044 (6/137)	0.071 (470/6632)	0.64	0.29	0.042 (14/337)	0.074 (492/6641)	0.55	0.032
HLA-DQB1*0202	0.146 (69/474)	0.186 (2468/13273)	0.73	0.019	0.124 (17/137)	0.193 (1282/6632)	0.58	0.036	0.154 (52/337)	0.179 (1186/6641)	0.83	0.22
HLA-DQA1*0301	0.289 (137/474)	0.357 (4735/13273)	0.79	0.021	0.314 (43/137)	0.358 (2373/6632)	0.91	0.61	0.279 (94/337)	0.356 (2362/6641)	0.75	0.021
HLA-C*1601	0.053 (25/474)	0.082 (1090/13273)	0.62	0.024	0.073 (10/137)	0.086 (573/6632)	0.84	0.6	0.045 (15/337)	0.078 (517/6641)	0.55	0.028
HLA-DRB3*0101	0.232 (110/474)	0.296 (3924/13273)	0.78	0.024	0.255 (35/137)	0.292 (1939/6632)	0.95	0.79	0.223 (75/337)	0.299 (1985/6641)	0.71	0.012
HLA-DRB4*0103	0.346 (164/474)	0.416 (5525/13273)	0.8	0.026	0.38 (52/137)	0.416 (2761/6632)	0.95	0.76	0.332 (112/337)	0.416 (2764/6641)	0.75	0.017
HLA-DRB4*9901	0.92 (436/474)	0.883 (11721/13273)	1.45	0.032	0.956 (131/137)	0.876 (5811/6632)	2.92	0.011	0.905 (305/337)	0.89 (5910/6641)	1.11	0.57
HLA-B*4402	0.15 (71/474)	0.205 (2721/13273)	0.76	0.033	0.095 (13/137)	0.205 (1361/6632)	0.47	0.01	0.172 (58/337)	0.205 (1360/6641)	0.87	0.36
HLA-A*2902	0.055 (26/474)	0.079 (1046/13273)	0.65	0.033	0.073 (10/137)	0.083 (552/6632)	0.81	0.53	0.047 (16/337)	0.074 (494/6641)	0.59	0.045
HLA-B*3502	0.044 (21/474)	0.014 (184/13273)	1.69	0.037	0.029 (4/137)	0.015 (99/6632)	0.71	0.53	0.05 (17/337)	0.013 (85/6641)	2.57	0.0011
HLA-DQA1*0401	0.063 (30/474)	0.047 (618/13273)	1.5	0.038	0.058 (8/137)	0.045 (298/6632)	1.49	0.29	0.065 (22/337)	0.048 (320/6641)	1.47	0.093
HLA-DPA1*0105	0.006 (3/474)	0.002 (20/13273)	3.64	0.041	0.007 (1/137)	0.001 (9/6632)	4.94	0.13	0.006 (2/337)	0.002 (11/6641)	3.18	0.14
HLA-C*0304	0.103 (49/474)	0.154 (2039/13273)	0.74	0.047	0.073 (10/137)	0.152 (1009/6632)	0.54	0.064	0.116 (39/337)	0.155 (1030/6641)	0.8	0.21
HLA-B*3906	0.023 (11/474)	0.014 (188/13273)	1.86	0.05	0.036 (5/137)	0.014 (96/6632)	3.04	0.019	0.018 (6/337)	0.014 (92/6641)	1.42	0.41
HLA-B*4403	0.076 (36/474)	0.105 (1397/13273)	0.71	0.05	0.102 (14/137)	0.11 (728/6632)	0.93	0.81	0.065 (22/337)	0.101 (669/6641)	0.63	0.042
HLA-DRB1*0404	0.057 (27/474)	0.089 (1183/13273)	0.68	0.051	0.029 (4/137)	0.088 (586/6632)	0.33	0.032	0.068 (23/337)	0.09 (597/6641)	0.82	0.38
HLA-DQB1*0302	0.156 (74/474)	0.198 (2625/13273)	0.79	0.062	0.168 (23/137)	0.199 (1317/6632)	0.86	0.53	0.151 (51/337)	0.197 (1308/6641)	0.76	0.078
HLA-A*0203	0.004 (2/474)	0.001 (17/13273)	4.04	0.063	0 (0/137)	0.002 (10/6632)	NA	0.98	0.006 (2/337)	0.001 (7/6641)	7.29	0.014
HLA-DRB1*1101	0.141 (67/474)	0.097 (1292/13273)	1.29	0.067	0.168 (23/137)	0.094 (626/6632)	1.54	0.071	0.131 (44/337)	0.1 (666/6641)	1.16	0.38

**HLA-DRB1*0402**	0.03 (14/474)	0.009 (123/13273)	1.73	0.067	0.036 (5/137)	0.01 (65/6632)	1.96	0.17	0.027 (9/337)	0.009 (58/6641)	1.71	0.16
**HLA-B*2705**	0.055 (26/474)	0.085 (1124/13273)	0.69	0.07	0.044 (6/137)	0.085 (564/6632)	0.55	0.16	0.059 (20/337)	0.084 (560/6641)	0.75	0.22
**HLA-DQA1*0102**	0.367 (174/474)	0.341 (4527/13273)	1.19	0.082	0.38 (52/137)	0.339 (2246/6632)	1.34	0.11	0.362 (122/337)	0.343 (2281/6641)	1.11	0.36
**HLA-B*3701**	0.04 (19/474)	0.028 (376/13273)	1.52	0.082	0.051 (7/137)	0.027 (181/6632)	2.02	0.079	0.036 (12/337)	0.029 (195/6641)	1.31	0.38
**HLA-B*4101**	0.021 (10/474)	0.007 (90/13273)	1.84	0.083	0.022 (3/137)	0.006 (42/6632)	1.57	0.48	0.021 (7/337)	0.007 (48/6641)	1.95	0.11
**HLA-DQB1*0402**	0.063 (30/474)	0.049 (645/13273)	1.39	0.088	0.058 (8/137)	0.048 (316/6632)	1.35	0.42	0.065 (22/337)	0.05 (329/6641)	1.4	0.14
**HLA-DRB1*1401**	0.032 (15/474)	0.048 (635/13273)	0.64	0.09	0.029 (4/137)	0.046 (305/6632)	0.59	0.31	0.033 (11/337)	0.05 (330/6641)	0.64	0.15
**HLA-DQB1*0503**	0.034 (16/474)	0.05 (662/13273)	0.65	0.093	0.036 (5/137)	0.048 (319/6632)	0.7	0.44	0.033 (11/337)	0.052 (343/6641)	0.62	0.12
**HLA-DPB1*1601**	0.002 (1/474)	0.012 (163/13273)	0.19	0.095	0 (0/137)	0.013 (85/6632)	NA	0.97	0.003 (1/337)	0.012 (78/6641)	0.28	0.21
**HLA-A*3002**	0.013 (6/474)	0.021 (282/13273)	0.5	0.096	0.007 (1/137)	0.018 (118/6632)	0.32	0.26	0.015 (5/337)	0.025 (164/6641)	0.52	0.15
**HLA-B*4405**	0.011 (5/474)	0.003 (43/13273)	2.24	0.098	0.022 (3/137)	0.003 (18/6632)	4.84	0.016	0.006 (2/337)	0.004 (25/6641)	1.26	0.76
**HLA-DPA1*0301**	0.004 (2/474)	0.001 (10/13273)	3.75	0.099	0 (0/137)	0.001 (4/6632)	NA	0.98	0.006 (2/337)	0.001 (6/6641)	5.19	0.053
**HLA-A*0302**	0.013 (6/474)	0.004 (53/13273)	2.07	0.1	0.015 (2/137)	0.003 (18/6632)	3.82	0.085	0.012 (4/337)	0.005 (35/6641)	1.53	0.43
**HLA-C*0704**	0.021 (10/474)	0.036 (477/13273)	0.6	0.11	0.007 (1/137)	0.037 (245/6632)	0.2	0.11	0.027 (9/337)	0.035 (232/6641)	0.76	0.44
**HLA-B*3801**	0.053 (25/474)	0.025 (328/13273)	1.43	0.11	0.036 (5/137)	0.023 (153/6632)	0.94	0.89	0.059 (20/337)	0.026 (175/6641)	1.62	0.059
**HLA-DPB1*0202**	0.006 (3/474)	0.014 (192/13273)	0.4	0.11	0 (0/137)	0.012 (77/6632)	NA	0.98	0.009 (3/337)	0.017 (115/6641)	0.46	0.18
**HLA-DPA1*0201**	0.316 (150/474)	0.275 (3652/13273)	1.17	0.12	0.307 (42/137)	0.278 (1842/6632)	1.06	0.77	0.32 (108/337)	0.273 (1810/6641)	1.25	0.066
**HLA-A*2601**	0.076 (36/474)	0.045 (601/13273)	1.33	0.12	0.066 (9/137)	0.044 (294/6632)	1.1	0.8	0.08 (27/337)	0.046 (307/6641)	1.43	0.1
**HLA-DRB1*1102**	0.015 (7/474)	0.006 (83/13273)	1.86	0.12	0.007 (1/137)	0.006 (42/6632)	0.92	0.94	0.018 (6/337)	0.006 (41/6641)	2.19	0.08
**HLA-DRB1*0407**	0.032 (15/474)	0.023 (302/13273)	1.51	0.13	0.022 (3/137)	0.023 (154/6632)	1.02	0.97	0.036 (12/337)	0.022 (148/6641)	1.71	0.079
**HLA-DRB1*0802**	0.004 (2/474)	0.002 (20/13273)	3.11	0.13	0.007 (1/137)	0.002 (10/6632)	5.75	0.1	0.003 (1/337)	0.002 (10/6641)	1.98	0.52
**HLA-DPB1*0402**	0.234 (111/474)	0.208 (2760/13273)	1.18	0.14	0.263 (36/137)	0.211 (1399/6632)	1.38	0.1	0.223 (75/337)	0.205 (1361/6641)	1.11	0.43
**HLA-C*0102**	0.051 (24/474)	0.068 (907/13273)	0.73	0.14	0.036 (5/137)	0.063 (419/6632)	0.58	0.24	0.056 (19/337)	0.073 (488/6641)	0.74	0.22
**HLA-DPB1*1701**	0.019 (9/474)	0.025 (332/13273)	0.6	0.14	0.015 (2/137)	0.023 (155/6632)	0.45	0.27	0.021 (7/337)	0.027 (177/6641)	0.65	0.27
**HLA-B*4901**	0.042 (20/474)	0.026 (349/13273)	1.41	0.15	0.058 (8/137)	0.026 (174/6632)	1.86	0.1	0.036 (12/337)	0.026 (175/6641)	1.23	0.49
**HLA-DRB1*0101**	0.184 (87/474)	0.173 (2290/13273)	1.19	0.16	0.153 (21/137)	0.173 (1148/6632)	0.98	0.93	0.196 (66/337)	0.172 (1142/6641)	1.28	0.082
**HLA-C*0602**	0.141 (67/474)	0.167 (2219/13273)	0.83	0.16	0.139 (19/137)	0.165 (1091/6632)	0.82	0.43	0.142 (48/337)	0.17 (1128/6641)	0.82	0.21
**HLA-DRB1*0801**	0.053 (25/474)	0.044 (584/13273)	1.34	0.17	0.044 (6/137)	0.042 (281/6632)	1.23	0.63	0.056 (19/337)	0.046 (303/6641)	1.34	0.23
**HLA-B*4002**	0.03 (14/474)	0.021 (278/13273)	1.46	0.18	0 (0/137)	0.021 (138/6632)	NA	0.97	0.042 (14/337)	0.021 (140/6641)	2.12	0.0092
**HLA-C*0303**	0.08 (38/474)	0.106 (1404/13273)	0.79	0.18	0.066 (9/137)	0.109 (722/6632)	0.64	0.21	0.086 (29/337)	0.103 (682/6641)	0.87	0.49
**HLA-B*4001**	0.078 (37/474)	0.109 (1441/13273)	0.79	0.18	0.058 (8/137)	0.107 (710/6632)	0.63	0.2	0.086 (29/337)	0.11 (731/6641)	0.85	0.4
**HLA-DPB1*0201**	0.228 (108/474)	0.237 (3152/13273)	0.86	0.19	0.204 (28/137)	0.234 (1555/6632)	0.74	0.16	0.237 (80/337)	0.24 (1597/6641)	0.91	0.48
**HLA-A*0202**	0.006 (3/474)	0.002 (22/13273)	2.29	0.19	0 (0/137)	0.002 (10/6632)	NA	0.98	0.009 (3/337)	0.002 (12/6641)	2.91	0.11
**HLA-C*1604**	0.004 (2/474)	0.001 (11/13273)	2.86	0.19	0.007 (1/137)	0.001 (4/6632)	4.47	0.23	0.003 (1/337)	0.001 (7/6641)	1.74	0.62
**HLA-B*0705**	0.002 (1/474)	0.004 (56/13273)	0.28	0.21	0 (0/137)	0.004 (28/6632)	NA	0.98	0.003 (1/337)	0.004 (28/6641)	0.39	0.36
**HLA-DPB1*1101**	0.036 (17/474)	0.049 (653/13273)	0.73	0.21	0.051 (7/137)	0.051 (335/6632)	1.02	0.96	0.03 (10/337)	0.048 (318/6641)	0.63	0.15
**HLA-DRB1*1103**	0.013 (6/474)	0.007 (87/13273)	1.71	0.21	0 (0/137)	0.007 (45/6632)	NA	0.97	0.018 (6/337)	0.006 (42/6641)	2.49	0.042
**HLA-B*3503**	0.034 (16/474)	0.023 (301/13273)	1.38	0.22	0.036 (5/137)	0.022 (146/6632)	1.52	0.37	0.033 (11/337)	0.023 (155/6641)	1.35	0.35
**HLA-DRB1*0901**	0.015 (7/474)	0.025 (331/13273)	0.63	0.23	0.015 (2/137)	0.024 (157/6632)	0.72	0.65	0.015 (5/337)	0.026 (174/6641)	0.59	0.25
**HLA-DPA1*0103**	0.973 (461/474)	0.964 (12798/13273)	1.39	0.25	0.985 (135/137)	0.961 (6375/6632)	2.98	0.13	0.967 (326/337)	0.967 (6423/6641)	1.02	0.96
**HLA-DRB1*1001**	0.023 (11/474)	0.014 (187/13273)	1.43	0.26	0.029 (4/137)	0.012 (82/6632)	2.05	0.17	0.021 (7/337)	0.016 (105/6641)	1.15	0.73
**HLA-A*3201**	0.055 (26/474)	0.07 (933/13273)	0.8	0.28	0.036 (5/137)	0.073 (486/6632)	0.52	0.15	0.062 (21/337)	0.067 (447/6641)	0.95	0.84
**HLA-A*6801**	0.046 (22/474)	0.063 (830/13273)	0.79	0.29	0.029 (4/137)	0.063 (416/6632)	0.51	0.18	0.053 (18/337)	0.062 (414/6641)	0.9	0.67
**HLA-B*5001**	0.017 (8/474)	0.02 (266/13273)	0.69	0.32	0.022 (3/137)	0.02 (134/6632)	0.82	0.74	0.015 (5/337)	0.02 (132/6641)	0.65	0.36
**HLA-A*0201**	0.487 (231/474)	0.485 (6438/13273)	1.1	0.32	0.482 (66/137)	0.487 (3229/6632)	1.08	0.66	0.49 (165/337)	0.483 (3209/6641)	1.11	0.37
**HLA-A*6802**	0.021 (10/474)	0.013 (172/13273)	1.38	0.33	0.007 (1/137)	0.013 (85/6632)	0.47	0.46	0.027 (9/337)	0.013 (87/6641)	1.77	0.11
**HLA-B*4701**	0.002 (1/474)	0.006 (79/13273)	0.41	0.37	0.007 (1/137)	0.006 (40/6632)	1.52	0.68	0 (0/337)	0.006 (39/6641)	NA	0.96
**HLA-B*4501**	0.011 (5/474)	0.015 (197/13273)	0.67	0.38	0 (0/137)	0.015 (102/6632)	NA	0.97	0.015 (5/337)	0.014 (95/6641)	1	0.99
**HLA-C*1505**	0.004 (2/474)	0.004 (58/13273)	0.53	0.38	0 (0/137)	0.004 (28/6632)	NA	0.98	0.006 (2/337)	0.005 (30/6641)	0.71	0.65
**HLA-DQB1*0303**	0.08 (38/474)	0.098 (1295/13273)	0.86	0.38	0.073 (10/137)	0.095 (632/6632)	0.82	0.55	0.083 (28/337)	0.1 (663/6641)	0.86	0.47
**HLA-DPB1*2001**	0.002 (1/474)	0.006 (76/13273)	0.42	0.39	0 (0/137)	0.006 (41/6632)	NA	0.97	0.003 (1/337)	0.005 (35/6641)	0.63	0.65
**HLA-A*0205**	0.019 (9/474)	0.019 (256/13273)	0.75	0.4	0.007 (1/137)	0.019 (124/6632)	0.26	0.19	0.024 (8/337)	0.02 (132/6641)	0.94	0.88
**HLA-DRB1*0102**	0.027 (13/474)	0.023 (306/13273)	0.78	0.4	0.036 (5/137)	0.024 (161/6632)	0.79	0.63	0.024 (8/337)	0.022 (145/6641)	0.79	0.53
**HLA-A*3004**	0.006 (3/474)	0.003 (36/13273)	1.65	0.42	0.015 (2/137)	0.004 (24/6632)	2.8	0.18	0.003 (1/337)	0.002 (12/6641)	1.15	0.89
**HLA-C*1801**	0.002 (1/474)	0.001 (7/13273)	2.38	0.43	0 (0/137)	0.001 (5/6632)	NA	0.98	0.003 (1/337)	0 (2/6641)	4.19	0.25

**HLA-B*3508**	0.017 (8/474)	0.009 (113/13273)	1.35	0.43	0.029 (4/137)	0.008 (56/6632)	2.1	0.17	0.012 (4/337)	0.009 (57/6641)	1.02	0.97
**HLA-A*3301**	0.015 (7/474)	0.014 (192/13273)	0.74	0.44	0.007 (1/137)	0.016 (104/6632)	0.29	0.23	0.018 (6/337)	0.013 (88/6641)	1.05	0.91
**HLA-B*1517**	0.011 (5/474)	0.005 (62/13273)	1.44	0.44	0.007 (1/137)	0.004 (25/6632)	1.3	0.8	0.012 (4/337)	0.006 (37/6641)	1.35	0.58
**HLA-DRB1*0301**	0.224 (106/474)	0.254 (3376/13273)	0.92	0.45	0.255 (35/137)	0.251 (1667/6632)	1.15	0.48	0.211 (71/337)	0.257 (1709/6641)	0.83	0.17
**HLA-DPB1*0501**	0.034 (16/474)	0.044 (580/13273)	0.83	0.46	0.029 (4/137)	0.045 (299/6632)	0.69	0.47	0.036 (12/337)	0.042 (281/6641)	0.92	0.79
**HLA-DPB1*0301**	0.213 (101/474)	0.205 (2718/13273)	1.08	0.48	0.241 (33/137)	0.205 (1361/6632)	1.29	0.21	0.202 (68/337)	0.204 (1357/6641)	1	0.98
**HLA-DQB1*0501**	0.241 (114/474)	0.228 (3020/13273)	1.08	0.48	0.219 (30/137)	0.227 (1508/6632)	0.94	0.75	0.249 (84/337)	0.228 (1512/6641)	1.14	0.31
**HLA-DPB1*0401**	0.658 (312/474)	0.68 (9031/13273)	0.94	0.51	0.657 (90/137)	0.677 (4491/6632)	0.94	0.73	0.659 (222/337)	0.684 (4540/6641)	0.92	0.49
**HLA-DRB1*0806**	0.002 (1/474)	0.001 (10/13273)	2.01	0.52	0.007 (1/137)	0.001 (4/6632)	14.09	0.02	0 (0/337)	0.001 (6/6641)	NA	0.97
**HLA-B*5801**	0.011 (5/474)	0.011 (144/13273)	0.74	0.52	0.015 (2/137)	0.011 (76/6632)	0.84	0.82	0.009 (3/337)	0.01 (68/6641)	0.69	0.54
**HLA-B*0702**	0.215 (102/474)	0.251 (3334/13273)	0.93	0.52	0.241 (33/137)	0.253 (1679/6632)	1.14	0.53	0.205 (69/337)	0.249 (1655/6641)	0.85	0.26
**HLA-DRB1*0408**	0.008 (4/474)	0.006 (86/13273)	1.35	0.56	0.015 (2/137)	0.007 (45/6632)	2.41	0.23	0.006 (2/337)	0.006 (41/6641)	0.94	0.93
**HLA-B*2702**	0.002 (1/474)	0.003 (36/13273)	0.55	0.56	0 (0/137)	0.003 (19/6632)	NA	0.97	0.003 (1/337)	0.003 (17/6641)	0.84	0.87
**HLA-B*5501**	0.04 (19/474)	0.035 (460/13273)	1.15	0.57	0.036 (5/137)	0.037 (244/6632)	1.06	0.9	0.042 (14/337)	0.033 (216/6641)	1.23	0.47
**HLA-B*3901**	0.027 (13/474)	0.022 (296/13273)	1.18	0.57	0.036 (5/137)	0.022 (143/6632)	1.72	0.25	0.024 (8/337)	0.023 (153/6641)	0.97	0.94
**HLA-C*0302**	0.002 (1/474)	0.003 (45/13273)	0.56	0.57	0 (0/137)	0.004 (24/6632)	NA	0.98	0.003 (1/337)	0.003 (21/6641)	0.9	0.92
**HLA-DRB1*1602**	0.004 (2/474)	0.002 (25/13273)	1.52	0.58	0 (0/137)	0.002 (12/6632)	NA	0.98	0.006 (2/337)	0.002 (13/6641)	2.34	0.27
**HLA-DRB5*0101**	0.238 (113/474)	0.248 (3292/13273)	1.06	0.58	0.27 (37/137)	0.25 (1655/6632)	1.35	0.13	0.226 (76/337)	0.246 (1637/6641)	0.96	0.73
**HLA-B*5101**	0.095 (45/474)	0.083 (1096/13273)	1.09	0.61	0.109 (15/137)	0.081 (534/6632)	1.29	0.36	0.089 (30/337)	0.085 (562/6641)	0.98	0.9
**HLA-DQB1*0201**	0.232 (110/474)	0.257 (3409/13273)	0.95	0.62	0.263 (36/137)	0.253 (1678/6632)	1.18	0.41	0.22 (74/337)	0.261 (1731/6641)	0.86	0.25
**HLA-C*0801**	0.002 (1/474)	0.002 (20/13273)	1.66	0.62	0.007 (1/137)	0.002 (12/6632)	5.58	0.1	0 (0/337)	0.001 (8/6641)	NA	0.97
**HLA-DPB1*2301**	0.002 (1/474)	0.004 (49/13273)	0.61	0.63	0.007 (1/137)	0.003 (19/6632)	3.04	0.28	0 (0/337)	0.005 (30/6641)	NA	0.96
**HLA-B*4801**	0.002 (1/474)	0.001 (19/13273)	1.62	0.64	0 (0/137)	0.002 (11/6632)	NA	0.98	0.003 (1/337)	0.001 (8/6641)	2.25	0.45
**HLA-DPA1*0104**	0.011 (5/474)	0.011 (143/13273)	0.81	0.64	0 (0/137)	0.012 (80/6632)	NA	0.97	0.015 (5/337)	0.009 (63/6641)	1.32	0.56
**HLA-DPA1*0202**	0.053 (25/474)	0.061 (816/13273)	0.91	0.64	0.066 (9/137)	0.064 (425/6632)	1.12	0.75	0.047 (16/337)	0.059 (391/6641)	0.85	0.52
**HLA-DRB1*1302**	0.095 (45/474)	0.087 (1150/13273)	1.08	0.65	0.08 (11/137)	0.083 (549/6632)	0.95	0.88	0.101 (34/337)	0.09 (601/6641)	1.09	0.64
**HLA-DRB1*1501**	0.236 (112/474)	0.248 (3292/13273)	1.05	0.65	0.27 (37/137)	0.25 (1657/6632)	1.35	0.13	0.223 (75/337)	0.246 (1635/6641)	0.94	0.66
**HLA-DQB1*0609**	0.025 (12/474)	0.023 (302/13273)	1.14	0.66	0.022 (3/137)	0.022 (147/6632)	1	1	0.027 (9/337)	0.023 (155/6641)	1.2	0.6
**HLA-A*2901**	0.006 (3/474)	0.004 (55/13273)	0.77	0.66	0.015 (2/137)	0.004 (26/6632)	1.72	0.49	0.003 (1/337)	0.004 (29/6641)	0.35	0.31
**HLA-A*0301**	0.253 (120/474)	0.26 (3456/13273)	1.05	0.67	0.219 (30/137)	0.259 (1718/6632)	0.89	0.56	0.267 (90/337)	0.262 (1738/6641)	1.11	0.4
**HLA-DRB1*1502**	0.013 (6/474)	0.01 (131/13273)	0.84	0.68	0 (0/137)	0.011 (72/6632)	NA	0.97	0.018 (6/337)	0.009 (59/6641)	1.38	0.47
**HLA-B*0801**	0.232 (110/474)	0.244 (3236/13273)	1.05	0.68	0.248 (34/137)	0.243 (1611/6632)	1.22	0.33	0.226 (76/337)	0.245 (1625/6641)	0.98	0.91
**HLA-DRB5*9901**	0.977 (463/474)	0.978 (12976/13273)	0.88	0.68	0.971 (133/137)	0.979 (6490/6632)	0.64	0.4	0.979 (330/337)	0.977 (6486/6641)	1.04	0.91
**HLA-DQB1*0601**	0.013 (6/474)	0.01 (130/13273)	0.84	0.69	0.007 (1/137)	0.01 (69/6632)	0.41	0.38	0.015 (5/337)	0.009 (61/6641)	1.09	0.86
**HLA-DQB1*0604**	0.07 (33/474)	0.063 (833/13273)	1.08	0.69	0.058 (8/137)	0.06 (395/6632)	0.96	0.9	0.074 (25/337)	0.066 (438/6641)	1.08	0.73
**HLA-DPB1*1001**	0.032 (15/474)	0.034 (453/13273)	0.91	0.72	0.007 (1/137)	0.034 (223/6632)	0.2	0.11	0.042 (14/337)	0.035 (230/6641)	1.23	0.47
**HLA-A*3101**	0.049 (23/474)	0.052 (694/13273)	0.93	0.72	0.058 (8/137)	0.051 (341/6632)	1.16	0.69	0.045 (15/337)	0.053 (353/6641)	0.82	0.47
**HLA-DPB1*0101**	0.11 (52/474)	0.113 (1502/13273)	1.05	0.73	0.117 (16/137)	0.117 (773/6632)	1.12	0.67	0.107 (36/337)	0.11 (729/6641)	1.06	0.76
**HLA-C*0802**	0.091 (43/474)	0.079 (1047/13273)	1.05	0.75	0.08 (11/137)	0.084 (555/6632)	0.79	0.47	0.095 (32/337)	0.074 (492/6641)	1.23	0.29
**HLA-C*1202**	0.015 (7/474)	0.011 (150/13273)	0.88	0.75	0.007 (1/137)	0.012 (78/6632)	0.34	0.29	0.018 (6/337)	0.011 (72/6641)	1.2	0.68
**HLA-DQB1*0304**	0.004 (2/474)	0.003 (43/13273)	1.26	0.75	0.015 (2/137)	0.003 (20/6632)	5.28	0.031	0 (0/337)	0.003 (23/6641)	NA	0.97
**HLA-A*2501**	0.036 (17/474)	0.04 (527/13273)	0.92	0.76	0.066 (9/137)	0.04 (268/6632)	1.65	0.16	0.024 (8/337)	0.039 (259/6641)	0.62	0.2
**HLA-DPB1*0601**	0.03 (14/474)	0.035 (458/13273)	0.92	0.76	0.022 (3/137)	0.032 (214/6632)	0.76	0.65	0.033 (11/337)	0.037 (244/6641)	0.94	0.85
**HLA-DPB1*1901**	0.008 (4/474)	0.011 (140/13273)	0.86	0.77	0.007 (1/137)	0.009 (61/6632)	0.84	0.86	0.009 (3/337)	0.012 (79/6641)	0.8	0.7
**HLA-B*1302**	0.036 (17/474)	0.038 (502/13273)	0.93	0.77	0.029 (4/137)	0.036 (239/6632)	0.75	0.58	0.039 (13/337)	0.04 (263/6641)	0.95	0.85
**HLA-DPB1*0901**	0.015 (7/474)	0.013 (179/13273)	1.12	0.78	0.022 (3/137)	0.014 (91/6632)	1.56	0.46	0.012 (4/337)	0.013 (88/6641)	0.93	0.88
**HLA-DRB1*0405**	0.011 (5/474)	0.011 (140/13273)	0.88	0.78	0.015 (2/137)	0.011 (74/6632)	1.15	0.85	0.009 (3/337)	0.01 (66/6641)	0.81	0.73
**HLA-DRB1*1201**	0.034 (16/474)	0.032 (421/13273)	1.08	0.78	0.022 (3/137)	0.032 (210/6632)	0.7	0.55	0.039 (13/337)	0.032 (211/6641)	1.22	0.5
**HLA-DRB3*0301**	0.091 (43/474)	0.085 (1133/13273)	1.04	0.81	0.073 (10/137)	0.082 (543/6632)	0.87	0.67	0.098 (33/337)	0.089 (590/6641)	1.08	0.69
**HLA-DRB3*9901**	0.852 (404/474)	0.863 (11459/13273)	1.03	0.81	0.869 (119/137)	0.865 (5736/6632)	1.21	0.47	0.846 (285/337)	0.862 (5723/6641)	0.98	0.89
**HLA-DRB1*1303**	0.023 (11/474)	0.019 (254/13273)	1.08	0.82	0.015 (2/137)	0.02 (131/6632)	0.63	0.52	0.027 (9/337)	0.019 (123/6641)	1.33	0.42
**HLA-A*3303**	0.004 (2/474)	0.004 (54/13273)	0.85	0.82	0.007 (1/137)	0.004 (29/6632)	1.32	0.79	0.003 (1/337)	0.004 (25/6641)	0.63	0.65
**HLA-A*0206**	0.002 (1/474)	0.003 (40/13273)	0.8	0.83	0 (0/137)	0.003 (20/6632)	NA	0.97	0.003 (1/337)	0.003 (20/6641)	1.17	0.88
**HLA-DRB1*0803**	0.004 (2/474)	0.004 (48/13273)	1.17	0.83	0.007 (1/137)	0.004 (26/6632)	2.07	0.49	0.003 (1/337)	0.003 (22/6641)	0.83	0.86
**HLA-DQA1*0101**	0.266 (126/474)	0.27 (3588/13273)	0.98	0.84	0.241 (33/137)	0.269 (1783/6632)	0.84	0.38	0.276 (93/337)	0.272 (1805/6641)	1.04	0.76

**HLA-DRB1*0403**	0.015 (7/474)	0.011 (144/13273)	1.08	0.85	0.029 (4/137)	0.012 (78/6632)	2.01	0.19	0.009 (3/337)	0.01 (66/6641)	0.7	0.55
**HLA-DRB1*0103**	0.03 (14/474)	0.031 (407/13273)	1.05	0.86	0.015 (2/137)	0.03 (197/6632)	0.52	0.37	0.036 (12/337)	0.032 (210/6641)	1.24	0.49
**HLA-C*0202**	0.082 (39/474)	0.079 (1054/13273)	1.03	0.87	0.073 (10/137)	0.08 (532/6632)	0.88	0.7	0.086 (29/337)	0.079 (522/6641)	1.1	0.62
**HLA-B*1401**	0.025 (12/474)	0.026 (340/13273)	1.04	0.89	0.029 (4/137)	0.028 (183/6632)	1.18	0.75	0.024 (8/337)	0.024 (157/6641)	1.03	0.93
**HLA-A*2301**	0.034 (16/474)	0.032 (423/13273)	0.97	0.9	0.029 (4/137)	0.032 (215/6632)	0.78	0.64	0.036 (12/337)	0.031 (208/6641)	1.07	0.82
**HLA-B*1402**	0.065 (31/474)	0.057 (756/13273)	0.98	0.92	0.051 (7/137)	0.06 (397/6632)	0.61	0.22	0.071 (24/337)	0.054 (359/6641)	1.21	0.39
**HLA-A*3001**	0.025 (12/474)	0.022 (290/13273)	1.03	0.93	0.036 (5/137)	0.02 (132/6632)	1.49	0.4	0.021 (7/337)	0.024 (158/6641)	0.78	0.54
**HLA-DRB1*1404**	0.002 (1/474)	0.002 (29/13273)	0.92	0.93	0.007 (1/137)	0.002 (13/6632)	3.1	0.29	0 (0/337)	0.002 (16/6641)	NA	0.96
**HLA-DQB1*0602**	0.226 (107/474)	0.246 (3271/13273)	1.01	0.94	0.255 (35/137)	0.248 (1643/6632)	1.28	0.23	0.214 (72/337)	0.245 (1628/6641)	0.91	0.49
**HLA-A*2402**	0.169 (80/474)	0.158 (2092/13273)	1.01	0.94	0.204 (28/137)	0.158 (1045/6632)	1.27	0.27	0.154 (52/337)	0.158 (1047/6641)	0.91	0.55
**HLA-DRB1*1305**	0 (0/474)	0.001 (19/13273)	NA	0.95	0 (0/137)	0.001 (7/6632)	NA	0.98	0 (0/337)	0.002 (12/6641)	NA	0.96
**HLA-DQA1*0601**	0.004 (2/474)	0.004 (53/13273)	1.05	0.95	0.007 (1/137)	0.005 (30/6632)	1.61	0.65	0.003 (1/337)	0.003 (23/6641)	0.83	0.85
**HLA-B*4404**	0 (0/474)	0.002 (22/13273)	NA	0.95	0 (0/137)	0.002 (10/6632)	NA	0.98	0 (0/337)	0.002 (12/6641)	NA	0.96
**HLA-B*4102**	0.013 (6/474)	0.009 (126/13273)	0.97	0.95	0 (0/137)	0.009 (62/6632)	NA	0.98	0.018 (6/337)	0.01 (64/6641)	1.37	0.48
**HLA-B*1516**	0 (0/474)	0.001 (17/13273)	NA	0.96	0 (0/137)	0.001 (6/6632)	NA	0.97	0 (0/337)	0.002 (11/6641)	NA	0.96
**HLA-A*6901**	0 (0/474)	0.002 (33/13273)	NA	0.96	0 (0/137)	0.002 (11/6632)	NA	0.98	0 (0/337)	0.003 (22/6641)	NA	0.96
**HLA-B*1503**	0.004 (2/474)	0.003 (37/13273)	1.04	0.96	0 (0/137)	0.003 (17/6632)	NA	0.97	0.006 (2/337)	0.003 (20/6641)	1.42	0.64
**HLA-B*1518**	0 (0/474)	0.006 (85/13273)	NA	0.96	0 (0/137)	0.005 (35/6632)	NA	0.97	0 (0/337)	0.008 (50/6641)	NA	0.97
**HLA-DQB1*0504**	0.002 (1/474)	0.003 (34/13273)	0.95	0.96	0 (0/137)	0.003 (17/6632)	NA	0.97	0.003 (1/337)	0.003 (17/6641)	1.31	0.8
**HLA-B*5703**	0 (0/474)	0.001 (11/13273)	NA	0.96	0 (0/137)	0.001 (5/6632)	NA	0.98	0 (0/337)	0.001 (6/6641)	NA	0.97
**HLA-A*3402**	0 (0/474)	0.002 (26/13273)	NA	0.97	0 (0/137)	0.002 (14/6632)	NA	0.97	0 (0/337)	0.002 (12/6641)	NA	0.96
**HLA-B*1524**	0 (0/474)	0.001 (12/13273)	NA	0.97	0 (0/137)	0.001 (5/6632)	NA	0.98	0 (0/337)	0.001 (7/6641)	NA	0.97
**HLA-DPB1*1501**	0.017 (8/474)	0.015 (197/13273)	0.98	0.97	0 (0/137)	0.016 (107/6632)	NA	0.97	0.024 (8/337)	0.014 (90/6641)	1.57	0.23
**HLA-A*0102**	0 (0/474)	0.001 (10/13273)	NA	0.97	0 (0/137)	0.001 (5/6632)	NA	0.98	0 (0/337)	0.001 (5/6641)	NA	0.96
**HLA-DRB1*0406**	0 (0/474)	0.001 (10/13273)	NA	0.97	0 (0/137)	0.001 (5/6632)	NA	0.98	0 (0/337)	0.001 (5/6641)	NA	0.96
**HLA-A*1101**	0.118 (56/474)	0.118 (1570/13273)	1.01	0.97	0.146 (20/137)	0.116 (772/6632)	1.31	0.27	0.107 (36/337)	0.12 (798/6641)	0.88	0.49
**HLA-C*1402**	0.021 (10/474)	0.018 (241/13273)	1.01	0.98	0.051 (7/137)	0.016 (103/6632)	3.04	0.0063	0.009 (3/337)	0.021 (138/6641)	0.37	0.089
**HLA-B*5301**	0.008 (4/474)	0.006 (81/13273)	0.99	0.98	0 (0/137)	0.007 (49/6632)	NA	0.97	0.012 (4/337)	0.005 (32/6641)	1.78	0.29
**HLA-DPB1*1401**	0.03 (14/474)	0.027 (360/13273)	1	0.99	0.022 (3/137)	0.028 (189/6632)	0.63	0.44	0.033 (11/337)	0.026 (171/6641)	1.25	0.49
**HLA-C*0702**	0.245 (116/474)	0.27 (3581/13273)	1	1	0.263 (36/137)	0.272 (1803/6632)	1.16	0.47	0.237 (80/337)	0.268 (1778/6641)	0.95	0.68
**HLA-A*0101**	0.312 (148/474)	0.323 (4282/13273)	1	1	0.299 (41/137)	0.323 (2139/6632)	0.94	0.76	0.318 (107/337)	0.323 (2143/6641)	1.03	0.82
**HLA-B*5201**	0.017 (8/474)	0.011 (152/13273)	1	1	0.007 (1/137)	0.012 (80/6632)	0.33	0.28	0.021 (7/337)	0.011 (72/6641)	1.43	0.39

Only alleles seen in >=10 controls in the meta-analysis cohort are shown. P-values below the GWS threshold are coloured orange, those with suggestive significance ($P<5x10^{-6}$) are coloured yellow. Rare alleles for which odds ratios could not be accurately computed are assigned OR = NA. CO = control, OR = odds ratio, NA = not applicable.

Supplementary Table 6: Classical HLA allele frequency differences between dSSc and lSSc in all three study cohorts.

	dSSC vs lSSC											
	Meta-analysis Cohort				Cohort 1				Cohort 2			
HLA Allele	lSSc Prop.(Count)	dSSc Prop.(Count)	OR	p	lSSc Prop.(Count)	dSSc Prop.(Count)	OR	p	lSSc Prop.(Count)	dSSc Prop.(Count)	OR	
HLA-DQB1*0202	0.08 (78/974)	0.146 (69/474)	2.04	6.20E-05	0.096 (34/353)	0.124 (17/137)	1.39	0.31	0.071 (44/621)	0.154 (52/337)	2.54	2.40E-05
HLA-DRB1*0701	0.126 (123/974)	0.205 (97/474)	1.83	6.20E-05	0.15 (53/353)	0.175 (24/137)	1.24	0.44	0.113 (70/621)	0.217 (73/337)	2.23	1.40E-05
HLA-DQA1*0201	0.127 (124/974)	0.205 (97/474)	1.81	8.00E-05	0.153 (54/353)	0.175 (24/137)	1.22	0.47	0.113 (70/621)	0.217 (73/337)	2.23	1.40E-05
HLA-B*4901	0.011 (11/974)	0.042 (20/474)	3.74	0.00057	0.008 (3/353)	0.058 (8/137)	8.38	0.0033	0.013 (8/621)	0.036 (12/337)	2.69	0.034
HLA-B*4403	0.038 (37/974)	0.076 (36/474)	2.27	0.00082	0.054 (19/353)	0.102 (14/137)	2.58	0.013	0.029 (18/621)	0.065 (22/337)	2.5	0.0055
HLA-DQB1*0302	0.231 (225/974)	0.156 (74/474)	0.61	0.00084	0.207 (73/353)	0.168 (23/137)	0.84	0.51	0.245 (152/621)	0.151 (51/337)	0.54	0.00072
HLA-DRB4*0101	0.094 (92/974)	0.143 (68/474)	1.68	0.0025	0.113 (40/353)	0.102 (14/137)	0.97	0.94	0.084 (52/621)	0.16 (54/337)	2.17	0.00022
HLA-DPB1*1301	0.061 (59/974)	0.108 (51/474)	1.83	0.0026	0.051 (18/353)	0.109 (15/137)	1.82	0.12	0.066 (41/621)	0.107 (36/337)	1.74	0.023
HLA-C*0304	0.16 (156/974)	0.103 (49/474)	0.61	0.0047	0.161 (57/353)	0.073 (10/137)	0.44	0.023	0.159 (99/621)	0.116 (39/337)	0.67	0.054
HLA-B*3701	0.016 (16/974)	0.04 (19/474)	2.61	0.0056	0.02 (7/353)	0.051 (7/137)	3.31	0.033	0.014 (9/621)	0.036 (12/337)	2.61	0.034
HLA-B*1501	0.113 (110/974)	0.065 (31/474)	0.57	0.0086	0.11 (39/353)	0.051 (7/137)	0.45	0.062	0.114 (71/621)	0.071 (24/337)	0.6	0.041
HLA-DPB1*1101	0.015 (15/974)	0.036 (17/474)	2.54	0.01	0.025 (9/353)	0.051 (7/137)	2.71	0.059	0.01 (6/621)	0.03 (10/337)	3.03	0.035
HLA-C*1701	0.016 (16/974)	0.04 (19/474)	2.37	0.014	0.011 (4/353)	0.036 (5/137)	4.17	0.052	0.019 (12/621)	0.042 (14/337)	2.17	0.057
HLA-DQA1*0101	0.328 (319/974)	0.266 (126/474)	0.74	0.017	0.334 (118/353)	0.241 (33/137)	0.61	0.035	0.324 (201/621)	0.276 (93/337)	0.79	0.11
HLA-C*1601	0.03 (29/974)	0.053 (25/474)	1.91	0.021	0.042 (15/353)	0.073 (10/137)	2.27	0.061	0.023 (14/621)	0.045 (15/337)	2.06	0.058
HLA-C*1502	0.039 (38/974)	0.068 (32/474)	1.76	0.023	0.037 (13/353)	0.066 (9/137)	1.73	0.23	0.04 (25/621)	0.068 (23/337)	1.67	0.088
HLA-B*2705	0.089 (87/974)	0.055 (26/474)	0.59	0.025	0.076 (27/353)	0.044 (6/137)	0.66	0.37	0.097 (60/621)	0.059 (20/337)	0.56	0.03
HLA-DRB1*0404	0.092 (90/974)	0.057 (27/474)	0.6	0.025	0.082 (29/353)	0.029 (4/137)	0.37	0.074	0.098 (61/621)	0.068 (23/337)	0.66	0.1
HLA-B*4001	0.117 (114/974)	0.078 (37/474)	0.64	0.027	0.133 (47/353)	0.058 (8/137)	0.43	0.039	0.108 (67/621)	0.086 (29/337)	0.77	0.26
HLA-C*0102	0.082 (80/974)	0.051 (24/474)	0.59	0.03	0.085 (30/353)	0.036 (5/137)	0.4	0.068	0.081 (50/621)	0.056 (19/337)	0.67	0.16
HLA-DQA1*0301	0.347 (338/974)	0.289 (137/474)	0.77	0.031	0.346 (122/353)	0.314 (43/137)	1	0.99	0.348 (216/621)	0.279 (94/337)	0.71	0.023
HLA-DQB1*0604	0.043 (42/974)	0.07 (33/474)	1.66	0.035	0.034 (12/353)	0.058 (8/137)	1.54	0.38	0.048 (30/621)	0.074 (25/337)	1.58	0.1
HLA-DRB1*1401	0.056 (55/974)	0.032 (15/474)	0.54	0.038	0.048 (17/353)	0.029 (4/137)	0.51	0.25	0.061 (38/621)	0.033 (11/337)	0.54	0.078
HLA-DQA1*0102	0.319 (311/974)	0.367 (174/474)	1.27	0.047	0.317 (112/353)	0.38 (52/137)	1.3	0.22	0.32 (199/621)	0.362 (122/337)	1.22	0.18
HLA-DQB1*0503	0.057 (56/974)	0.034 (16/474)	0.57	0.052	0.051 (18/353)	0.036 (5/137)	0.56	0.28	0.061 (38/621)	0.033 (11/337)	0.54	0.083
HLA-DRB1*1302	0.067 (65/974)	0.095 (45/474)	1.48	0.054	0.057 (20/353)	0.08 (11/137)	1.25	0.57	0.072 (45/621)	0.101 (34/337)	1.48	0.11
HLA-DRB1*1201	0.017 (17/974)	0.034 (16/474)	1.95	0.06	0.023 (8/353)	0.022 (3/137)	0.97	0.97	0.014 (9/621)	0.039 (13/337)	2.9	0.017
HLA-DQB1*0301	0.386 (376/974)	0.445 (211/474)	1.24	0.063	0.371 (131/353)	0.467 (64/137)	1.33	0.18	0.395 (245/621)	0.436 (147/337)	1.19	0.21
HLA-DRB3*0301	0.065 (63/974)	0.091 (43/474)	1.46	0.068	0.057 (20/353)	0.073 (10/137)	1.14	0.76	0.069 (43/621)	0.098 (33/337)	1.5	0.099
HLA-DQB1*0501	0.283 (276/974)	0.241 (114/474)	0.8	0.084	0.292 (103/353)	0.219 (30/137)	0.67	0.11	0.279 (173/621)	0.249 (84/337)	0.84	0.27
HLA-C*1505	0.012 (12/974)	0.004 (2/474)	0.27	0.096	0.014 (5/353)	0 (0/137)	NA	0.98	0.011 (7/621)	0.006 (2/337)	0.46	0.35
HLA-B*4402	0.187 (182/974)	0.15 (71/474)	0.79	0.12	0.218 (77/353)	0.095 (13/137)	0.41	0.0063	0.169 (105/621)	0.172 (58/337)	1.01	0.97
HLA-DRB1*0101	0.221 (215/974)	0.184 (87/474)	0.8	0.13	0.235 (83/353)	0.153 (21/137)	0.61	0.071	0.213 (132/621)	0.196 (66/337)	0.89	0.49
HLA-C*0802	0.067 (65/974)	0.091 (43/474)	1.37	0.13	0.054 (19/353)	0.08 (11/137)	1.35	0.47	0.074 (46/621)	0.095 (32/337)	1.35	0.22
HLA-C*0701	0.32 (312/974)	0.357 (169/474)	1.19	0.14	0.348 (123/353)	0.401 (55/137)	1.28	0.25	0.304 (189/621)	0.338 (114/337)	1.17	0.29
HLA-A*2902	0.038 (37/974)	0.055 (26/474)	1.45	0.16	0.037 (13/353)	0.073 (10/137)	2.38	0.055	0.039 (24/621)	0.047 (16/337)	1.24	0.52
HLA-C*0501	0.174 (169/974)	0.141 (67/474)	0.8	0.17	0.207 (73/353)	0.08 (11/137)	0.34	0.0024	0.155 (96/621)	0.166 (56/337)	1.09	0.63
HLA-DRB3*9901	0.828 (806/974)	0.852 (404/474)	1.24	0.17	0.841 (297/353)	0.869 (119/137)	1.57	0.14	0.82 (509/621)	0.846 (285/337)	1.18	0.38
HLA-DQA1*0103	0.089 (87/974)	0.07 (33/474)	0.75	0.18	0.085 (30/353)	0.044 (6/137)	0.48	0.11	0.092 (57/621)	0.08 (27/337)	0.86	0.55
HLA-DPA1*0103	0.959 (934/974)	0.973 (461/474)	1.55	0.18	0.958 (338/353)	0.985 (135/137)	3.73	0.089	0.96 (596/621)	0.967 (326/337)	1.23	0.57
HLA-DPB1*1401	0.043 (42/974)	0.03 (14/474)	0.66	0.18	0.028 (10/353)	0.022 (3/137)	0.63	0.5	0.052 (32/621)	0.033 (11/337)	0.61	0.17
HLA-C*1203	0.11 (107/974)	0.139 (66/474)	1.26	0.19	0.096 (34/353)	0.161 (22/137)	1.61	0.12	0.118 (73/621)	0.131 (44/337)	1.24	0.32
HLA-DQA1*0501	0.495 (482/974)	0.536 (254/474)	1.16	0.19	0.482 (170/353)	0.584 (80/137)	1.44	0.081	0.502 (312/621)	0.516 (174/337)	1.07	0.64
HLA-B*1302	0.024 (23/974)	0.036 (17/474)	1.54	0.19	0.02 (7/353)	0.029 (4/137)	1.04	0.95	0.026 (16/621)	0.039 (13/337)	1.58	0.24
HLA-B*5801	0.018 (18/974)	0.011 (5/474)	0.51	0.19	0.02 (7/353)	0.015 (2/137)	0.57	0.49	0.018 (11/621)	0.009 (3/337)	0.49	0.28
HLA-DRB3*0101	0.271 (264/974)	0.232 (110/474)	0.84	0.19	0.275 (97/353)	0.255 (35/137)	1.02	0.94	0.269 (167/621)	0.223 (75/337)	0.77	0.12
HLA-DRB4*9901	0.937 (913/974)	0.92 (436/474)	0.76	0.2	0.915 (323/353)	0.956 (131/137)	1.88	0.18	0.95 (590/621)	0.905 (305/337)	0.5	0.0082
HLA-B*3801	0.035 (34/974)	0.053 (25/474)	1.43	0.2	0.025 (9/353)	0.036 (5/137)	1.28	0.68	0.04 (25/621)	0.059 (20/337)	1.67	0.11
HLA-C*0704	0.035 (34/974)	0.021 (10/474)	0.63	0.2	0.028 (10/353)	0.007 (1/137)	0.24	0.18	0.039 (24/621)	0.027 (9/337)	0.68	0.34

**HLA-DPB1*1001**	0.045 (44/974)	0.032 (15/474)	0.68	0.2	0.059 (21/353)	0.007 (1/137)	0.11	0.037	0.037 (23/621)	0.042 (14/337)	1.09	0.81
**HLA-A*2402**	0.192 (187/974)	0.169 (80/474)	0.83	0.21	0.19 (67/353)	0.204 (28/137)	1.04	0.88	0.193 (120/621)	0.154 (52/337)	0.75	0.12
**HLA-A*6801**	0.063 (61/974)	0.046 (22/474)	0.73	0.22	0.054 (19/353)	0.029 (4/137)	0.57	0.32	0.068 (42/621)	0.053 (18/337)	0.74	0.3
**HLA-DRB1*0801**	0.071 (69/974)	0.053 (25/474)	0.75	0.23	0.079 (28/353)	0.044 (6/137)	0.66	0.37	0.066 (41/621)	0.056 (19/337)	0.83	0.53
**HLA-DRB4*0103**	0.379 (369/974)	0.346 (164/474)	0.87	0.23	0.388 (137/353)	0.38 (52/137)	1.06	0.8	0.374 (232/621)	0.332 (112/337)	0.82	0.18
**HLA-DQB1*0303**	0.064 (62/974)	0.08 (38/474)	1.29	0.24	0.074 (26/353)	0.073 (10/137)	1.01	0.97	0.058 (36/621)	0.083 (28/337)	1.45	0.16
**HLA-A*6802**	0.012 (12/974)	0.021 (10/474)	1.65	0.25	0.011 (4/353)	0.007 (1/137)	0.62	0.68	0.013 (8/621)	0.027 (9/337)	2.17	0.12
**HLA-DRB1*0301**	0.259 (252/974)	0.224 (106/474)	0.86	0.26	0.278 (98/353)	0.255 (35/137)	1	1	0.248 (154/621)	0.211 (71/337)	0.81	0.21
**HLA-DQB1*0402**	0.08 (78/974)	0.063 (30/474)	0.78	0.27	0.088 (31/353)	0.058 (8/137)	0.75	0.49	0.076 (47/621)	0.065 (22/337)	0.84	0.51
**HLA-C*1402**	0.031 (30/974)	0.021 (10/474)	0.67	0.28	0.023 (8/353)	0.051 (7/137)	2.23	0.14	0.035 (22/621)	0.009 (3/337)	0.23	0.018
**HLA-B*5501**	0.029 (28/974)	0.04 (19/474)	1.39	0.28	0.031 (11/353)	0.036 (5/137)	0.95	0.93	0.027 (17/621)	0.042 (14/337)	1.57	0.22
**HLA-DRB1*0401**	0.163 (159/974)	0.139 (66/474)	0.84	0.28	0.184 (65/353)	0.161 (22/137)	0.97	0.9	0.151 (94/621)	0.131 (44/337)	0.83	0.35
**HLA-DRB1*1301**	0.076 (74/974)	0.061 (29/474)	0.79	0.29	0.071 (25/353)	0.044 (6/137)	0.57	0.24	0.079 (49/621)	0.068 (23/337)	0.85	0.54
**HLA-DRB1*1303**	0.014 (14/974)	0.023 (11/474)	1.54	0.29	0.014 (5/353)	0.015 (2/137)	0.94	0.95	0.014 (9/621)	0.027 (9/337)	1.75	0.24
**HLA-A*0201**	0.464 (452/974)	0.487 (231/474)	1.12	0.3	0.47 (166/353)	0.482 (66/137)	1.08	0.71	0.461 (286/621)	0.49 (165/337)	1.13	0.37
**HLA-B*5701**	0.055 (54/974)	0.042 (20/474)	0.76	0.31	0.048 (17/353)	0.044 (6/137)	0.93	0.89	0.06 (37/621)	0.042 (14/337)	0.67	0.21
**HLA-C*0602**	0.123 (120/974)	0.141 (67/474)	1.18	0.31	0.122 (43/353)	0.139 (19/137)	1.19	0.57	0.124 (77/621)	0.142 (48/337)	1.18	0.41
**HLA-DPA1*0201**	0.292 (284/974)	0.316 (150/474)	1.13	0.32	0.295 (104/353)	0.307 (42/137)	1.08	0.73	0.29 (180/621)	0.32 (108/337)	1.17	0.3
**HLA-B*1801**	0.112 (109/974)	0.131 (62/474)	1.18	0.35	0.125 (44/353)	0.168 (23/137)	1.18	0.57	0.105 (65/621)	0.116 (39/337)	1.17	0.48
**HLA-DQA1*0401**	0.078 (76/974)	0.063 (30/474)	0.81	0.35	0.085 (30/353)	0.058 (8/137)	0.81	0.62	0.074 (46/621)	0.065 (22/337)	0.85	0.56
**HLA-DRB1*0103**	0.023 (22/974)	0.03 (14/474)	1.38	0.36	0.031 (11/353)	0.015 (2/137)	0.62	0.54	0.018 (11/621)	0.036 (12/337)	2.04	0.097
**HLA-B*3906**	0.016 (16/974)	0.023 (11/474)	1.43	0.37	0.014 (5/353)	0.036 (5/137)	2.96	0.099	0.018 (11/621)	0.018 (6/337)	0.97	0.95
**HLA-DPB1*0301**	0.236 (230/974)	0.213 (101/474)	0.89	0.38	0.21 (74/353)	0.241 (33/137)	1.33	0.24	0.251 (156/621)	0.202 (68/337)	0.77	0.12
**HLA-A*3002**	0.018 (18/974)	0.013 (6/474)	0.66	0.39	0.011 (4/353)	0.007 (1/137)	0.61	0.67	0.023 (14/621)	0.015 (5/337)	0.66	0.43
**HLA-DRB1*0402**	0.033 (32/974)	0.03 (14/474)	0.75	0.39	0.02 (7/353)	0.036 (5/137)	1.31	0.67	0.04 (25/621)	0.027 (9/337)	0.69	0.37
**HLA-DQB1*0201**	0.261 (254/974)	0.232 (110/474)	0.89	0.4	0.286 (101/353)	0.263 (36/137)	1.01	0.96	0.246 (153/621)	0.22 (74/337)	0.86	0.37
**HLA-DPB1*0501**	0.043 (42/974)	0.034 (16/474)	0.78	0.41	0.054 (19/353)	0.029 (4/137)	0.5	0.23	0.037 (23/621)	0.036 (12/337)	0.95	0.89
**HLA-DRB3*0202**	0.37 (360/974)	0.403 (191/474)	1.1	0.41	0.314 (111/353)	0.438 (60/137)	1.44	0.097	0.401 (249/621)	0.389 (131/337)	0.96	0.76
**HLA-A*2501**	0.046 (45/974)	0.036 (17/474)	0.8	0.43	0.054 (19/353)	0.066 (9/137)	1.31	0.53	0.042 (26/621)	0.024 (8/337)	0.59	0.2
**HLA-C*1604**	0.006 (6/974)	0.004 (2/474)	0.52	0.43	0.003 (1/353)	0.007 (1/137)	2.15	0.62	0.008 (5/621)	0.003 (1/337)	0.32	0.3
**HLA-DRB1*1501**	0.225 (219/974)	0.236 (112/474)	1.11	0.44	0.238 (84/353)	0.27 (37/137)	1.3	0.27	0.217 (135/621)	0.223 (75/337)	1.02	0.89
**HLA-DPB1*0101**	0.101 (98/974)	0.11 (52/474)	1.15	0.44	0.096 (34/353)	0.117 (16/137)	1.45	0.27	0.103 (64/621)	0.107 (36/337)	1.09	0.71
**HLA-DPB1*0601**	0.039 (38/974)	0.03 (14/474)	0.78	0.44	0.037 (13/353)	0.022 (3/137)	0.68	0.56	0.04 (25/621)	0.033 (11/337)	0.78	0.51
**HLA-DQB1*0603**	0.079 (77/974)	0.07 (33/474)	0.85	0.45	0.068 (24/353)	0.051 (7/137)	0.71	0.45	0.085 (53/621)	0.077 (26/337)	0.88	0.61
**HLA-DRB5*0101**	0.228 (222/974)	0.238 (113/474)	1.1	0.47	0.238 (84/353)	0.27 (37/137)	1.3	0.27	0.222 (138/621)	0.226 (76/337)	1.01	0.93
**HLA-DPB1*1701**	0.025 (24/974)	0.019 (9/474)	0.75	0.47	0.028 (10/353)	0.015 (2/137)	0.53	0.42	0.023 (14/621)	0.021 (7/337)	0.92	0.87
**HLA-DRB1*0803**	0.006 (6/974)	0.004 (2/474)	0.58	0.51	0.006 (2/353)	0.007 (1/137)	0.64	0.73	0.006 (4/621)	0.003 (1/337)	0.42	0.44
**HLA-DRB1*0407**	0.027 (26/974)	0.032 (15/474)	1.24	0.52	0.034 (12/353)	0.022 (3/137)	0.73	0.63	0.023 (14/621)	0.036 (12/337)	1.62	0.23
**HLA-DQB1*0602**	0.219 (213/974)	0.226 (107/474)	1.09	0.52	0.229 (81/353)	0.255 (35/137)	1.29	0.3	0.213 (132/621)	0.214 (72/337)	1	0.98
**HLA-A*3201**	0.061 (59/974)	0.055 (26/474)	0.86	0.53	0.059 (21/353)	0.036 (5/137)	0.46	0.15	0.061 (38/621)	0.062 (21/337)	0.98	0.93
**HLA-DPB1*0201**	0.208 (203/974)	0.228 (108/474)	1.09	0.53	0.193 (68/353)	0.204 (28/137)	1.02	0.95	0.217 (135/621)	0.237 (80/337)	1.1	0.55
**HLA-DQB1*0609**	0.021 (20/974)	0.025 (12/474)	1.26	0.54	0.023 (8/353)	0.022 (3/137)	0.84	0.8	0.019 (12/621)	0.027 (9/337)	1.49	0.38
**HLA-DPB1*0202**	0.01 (10/974)	0.006 (3/474)	0.66	0.54	0.006 (2/353)	0 (0/137)	NA	0.98	0.013 (8/621)	0.009 (3/337)	0.7	0.6
**HLA-A*0205**	0.022 (21/974)	0.019 (9/474)	0.78	0.55	0.02 (7/353)	0.007 (1/137)	0.29	0.26	0.023 (14/621)	0.024 (8/337)	1.12	0.81
**HLA-DPB1*0901**	0.018 (18/974)	0.015 (7/474)	0.77	0.56	0.025 (9/353)	0.022 (3/137)	0.66	0.55	0.014 (9/621)	0.012 (4/337)	0.86	0.81
**HLA-B*0801**	0.253 (246/974)	0.232 (110/474)	0.93	0.59	0.261 (92/353)	0.248 (34/137)	1.09	0.71	0.248 (154/621)	0.226 (76/337)	0.89	0.46
**HLA-C*0303**	0.092 (90/974)	0.08 (38/474)	0.9	0.59	0.099 (35/353)	0.066 (9/137)	0.63	0.24	0.089 (55/621)	0.086 (29/337)	1.02	0.92
**HLA-B*3502**	0.034 (33/974)	0.044 (21/474)	1.16	0.61	0.023 (8/353)	0.029 (4/137)	0.73	0.63	0.04 (25/621)	0.05 (17/337)	1.27	0.48
**HLA-B*0702**	0.234 (228/974)	0.215 (102/474)	0.93	0.61	0.269 (95/353)	0.241 (33/137)	0.96	0.86	0.214 (133/621)	0.205 (69/337)	0.94	0.72
**HLA-DRB1*0901**	0.018 (18/974)	0.015 (7/474)	0.8	0.62	0.023 (8/353)	0.015 (2/137)	0.77	0.75	0.016 (10/621)	0.015 (5/337)	0.89	0.83
**HLA-DRB1*1001**	0.018 (18/974)	0.023 (11/474)	1.2	0.64	0.014 (5/353)	0.029 (4/137)	2.31	0.24	0.021 (13/621)	0.021 (7/337)	0.9	0.83
**HLA-A*2301**	0.03 (29/974)	0.034 (16/474)	1.15	0.65	0.042 (15/353)	0.029 (4/137)	0.65	0.47	0.023 (14/621)	0.036 (12/337)	1.6	0.24
**HLA-C*0702**	0.263 (256/974)	0.245 (116/474)	0.94	0.66	0.297 (105/353)	0.263 (36/137)	0.94	0.8	0.243 (151/621)	0.237 (80/337)	0.96	0.82
**HLA-A*2601**	0.077 (75/974)	0.076 (36/474)	0.91	0.66	0.062 (22/353)	0.066 (9/137)	0.86	0.73	0.085 (53/621)	0.08 (27/337)	0.91	0.72
**HLA-DRB5*9901**	0.973 (948/974)	0.977 (463/474)	1.15	0.71	0.98 (346/353)	0.971 (133/137)	0.7	0.58	0.969 (602/621)	0.979 (330/337)	1.6	0.3

**HLA-B*4002**	0.025 (24/974)	0.03 (14/474)	1.14	0.71	0.017 (6/353)	0 (0/137)	NA	0.98	0.029 (18/621)	0.042 (14/337)	1.32	0.46
**HLA-A*3301**	0.017 (17/974)	0.015 (7/474)	0.85	0.72	0.008 (3/353)	0.007 (1/137)	0.92	0.94	0.023 (14/621)	0.018 (6/337)	0.76	0.59
**HLA-C*1202**	0.016 (16/974)	0.015 (7/474)	0.85	0.73	0.02 (7/353)	0.007 (1/137)	0.36	0.35	0.014 (9/621)	0.018 (6/337)	1.22	0.71
**HLA-DRB1*1601**	0.034 (33/974)	0.04 (19/474)	1.11	0.73	0.023 (8/353)	0.044 (6/137)	1.53	0.45	0.04 (25/621)	0.039 (13/337)	0.93	0.83
**HLA-DRB1*1104**	0.114 (111/974)	0.131 (62/474)	1.06	0.75	0.076 (27/353)	0.168 (23/137)	1.89	0.058	0.135 (84/621)	0.116 (39/337)	0.8	0.31
**HLA-B*1402**	0.06 (58/974)	0.065 (31/474)	1.07	0.78	0.045 (16/353)	0.051 (7/137)	0.89	0.81	0.068 (42/621)	0.071 (24/337)	1.09	0.76
**HLA-B*3508**	0.017 (17/974)	0.017 (8/474)	0.89	0.78	0.011 (4/353)	0.029 (4/137)	2.2	0.29	0.021 (13/621)	0.012 (4/337)	0.58	0.35
**HLA-DRB1*0102**	0.028 (27/974)	0.027 (13/474)	0.91	0.8	0.02 (7/353)	0.036 (5/137)	1.26	0.72	0.032 (20/621)	0.024 (8/337)	0.73	0.47
**HLA-DRB1*1502**	0.011 (11/974)	0.013 (6/474)	1.14	0.8	0.017 (6/353)	0 (0/137)	NA	0.98	0.008 (5/621)	0.018 (6/337)	2.43	0.15
**HLA-A*0101**	0.311 (303/974)	0.312 (148/474)	1.03	0.81	0.351 (124/353)	0.299 (41/137)	0.83	0.42	0.288 (179/621)	0.318 (107/337)	1.14	0.38
**HLA-DPB1*0401**	0.664 (647/974)	0.658 (312/474)	0.97	0.81	0.691 (244/353)	0.657 (90/137)	0.86	0.5	0.649 (403/621)	0.659 (222/337)	1.02	0.9
**HLA-DQB1*0601**	0.011 (11/974)	0.013 (6/474)	1.12	0.83	0.017 (6/353)	0.007 (1/137)	0.41	0.42	0.008 (5/621)	0.015 (5/337)	1.93	0.3
**HLA-DRB1*1101**	0.132 (129/974)	0.141 (67/474)	1.04	0.83	0.122 (43/353)	0.168 (23/137)	1.28	0.4	0.138 (86/621)	0.131 (44/337)	0.94	0.75
**HLA-B*5101**	0.09 (88/974)	0.095 (45/474)	1.04	0.85	0.074 (26/353)	0.109 (15/137)	1.31	0.46	0.1 (62/621)	0.089 (30/337)	0.85	0.48
**HLA-DRB1*0403**	0.016 (16/974)	0.015 (7/474)	0.92	0.86	0.011 (4/353)	0.029 (4/137)	3.36	0.099	0.019 (12/621)	0.009 (3/337)	0.41	0.18
**HLA-B*4501**	0.011 (11/974)	0.011 (5/474)	0.91	0.87	0.011 (4/353)	0 (0/137)	NA	0.98	0.011 (7/621)	0.015 (5/337)	1.29	0.67
**HLA-DPA1*0202**	0.05 (49/974)	0.053 (25/474)	1.04	0.88	0.045 (16/353)	0.066 (9/137)	1.5	0.36	0.053 (33/621)	0.047 (16/337)	0.89	0.71
**HLA-B*3501**	0.139 (135/974)	0.137 (65/474)	0.98	0.88	0.11 (39/353)	0.146 (20/137)	1.5	0.19	0.155 (96/621)	0.134 (45/337)	0.84	0.36
**HLA-C*0401**	0.237 (231/974)	0.241 (114/474)	0.98	0.88	0.193 (68/353)	0.255 (35/137)	1.35	0.23	0.262 (163/621)	0.234 (79/337)	0.85	0.32
**HLA-DRB1*1103**	0.012 (12/974)	0.013 (6/474)	1.08	0.88	0.011 (4/353)	0 (0/137)	NA	0.98	0.013 (8/621)	0.018 (6/337)	1.49	0.47
**HLA-C*0202**	0.081 (79/974)	0.082 (39/474)	0.97	0.89	0.059 (21/353)	0.073 (10/137)	1.22	0.63	0.093 (58/621)	0.086 (29/337)	0.86	0.52
**HLA-A*1101**	0.116 (113/974)	0.118 (56/474)	1.02	0.9	0.096 (34/353)	0.146 (20/137)	1.63	0.11	0.127 (79/621)	0.107 (36/337)	0.82	0.35
**HLA-B*3503**	0.034 (33/974)	0.034 (16/474)	0.97	0.92	0.04 (14/353)	0.036 (5/137)	0.71	0.54	0.031 (19/621)	0.033 (11/337)	1.12	0.78
**HLA-B*5001**	0.015 (15/974)	0.017 (8/474)	1.04	0.92	0.023 (8/353)	0.022 (3/137)	0.97	0.97	0.011 (7/621)	0.015 (5/337)	1.44	0.54
**HLA-DQB1*0502**	0.044 (43/974)	0.049 (23/474)	1.03	0.92	0.034 (12/353)	0.051 (7/137)	1.12	0.82	0.05 (31/621)	0.047 (16/337)	0.94	0.84
**HLA-B*5601**	0.023 (22/974)	0.023 (11/474)	1.04	0.92	0.028 (10/353)	0.015 (2/137)	0.59	0.5	0.019 (12/621)	0.027 (9/337)	1.51	0.36
**HLA-DRB5*0202**	0.037 (36/974)	0.04 (19/474)	1.02	0.93	0.025 (9/353)	0.036 (5/137)	1.08	0.9	0.043 (27/621)	0.042 (14/337)	0.94	0.86
**HLA-A*0301**	0.257 (250/974)	0.253 (120/474)	1.01	0.94	0.258 (91/353)	0.219 (30/137)	0.86	0.55	0.256 (159/621)	0.267 (90/337)	1.07	0.66
**HLA-DPB1*0402**	0.235 (229/974)	0.234 (111/474)	0.99	0.96	0.227 (80/353)	0.263 (36/137)	1.19	0.46	0.24 (149/621)	0.223 (75/337)	0.94	0.72
**HLA-DRB1*0405**	0.011 (11/974)	0.011 (5/474)	0.97	0.96	0.017 (6/353)	0.015 (2/137)	0.96	0.97	0.008 (5/621)	0.009 (3/337)	1.12	0.87
**HLA-A*3101**	0.048 (47/974)	0.049 (23/474)	1.01	0.96	0.042 (15/353)	0.058 (8/137)	1.41	0.47	0.052 (32/621)	0.045 (15/337)	0.89	0.72
**HLA-B*5201**	0.016 (16/974)	0.017 (8/474)	0.98	0.96	0.02 (7/353)	0.007 (1/137)	0.36	0.35	0.014 (9/621)	0.021 (7/337)	1.44	0.48
**HLA-B*3901**	0.027 (26/974)	0.027 (13/474)	1.01	0.98	0.02 (7/353)	0.036 (5/137)	2.14	0.22	0.031 (19/621)	0.024 (8/337)	0.79	0.59

Only alleles seen in >=10 lSSc patients in the meta-analysis cohort are shown. Rare alleles for which odds ratios could not be accurately computed are assigned OR = NA.
CO = control, OR = odds ratio, NA = not applicable.

Supplementary Table 7: Classical HLA allele frequency differences between ACA+ and ACA- SSc in all three study cohorts.

	ACA+ vs -											
	Meta-analysis Cohort				Cohort 1				Cohort 2			
HLA Allele	ACA- Prop.(Count)	ACA+ Prop.(Count)	OR	p	ACA- Prop.(Count)	ACA+ Prop.(Count)	OR	p	ACA- Prop.(Count)	ACA+ Prop.(Count)	OR	p
HLADQA1_0101	0.233 (210/903)	0.436 (221/507)	2.58	4.50E-15	0.241 (64/266)	0.401 (87/217)	2.21	0.00012	0.229 (146/637)	0.462 (134/290)	2.99	1.20E-12
HLADQB1_0501	0.204 (184/903)	0.379 (192/507)	2.39	3.20E-12	0.214 (57/266)	0.35 (76/217)	2.02	9.00E-04	0.199 (127/637)	0.4 (116/290)	2.76	1.80E-10
HLADQA1_0201	0.196 (177/903)	0.077 (39/507)	0.34	6.70E-09	0.207 (55/266)	0.106 (23/217)	0.45	0.0037	0.192 (122/637)	0.055 (16/290)	0.24	3.10E-07
HLADRB1_0701	0.195 (176/903)	0.077 (39/507)	0.34	9.30E-09	0.203 (54/266)	0.106 (23/217)	0.47	0.0056	0.192 (122/637)	0.055 (16/290)	0.24	3.10E-07
HLADRB1_0101	0.159 (144/903)	0.292 (148/507)	2.15	1.50E-08	0.162 (43/266)	0.281 (61/217)	2.04	0.0019	0.159 (101/637)	0.3 (87/290)	2.33	8.70E-07
HLADQB1_0202	0.134 (121/903)	0.043 (22/507)	0.29	2.80E-07	0.147 (39/266)	0.055 (12/217)	0.35	0.0025	0.129 (82/637)	0.034 (10/290)	0.23	2.10E-05
HLADRB1_0801	0.039 (35/903)	0.112 (57/507)	3.15	3.90E-07	0.041 (11/266)	0.106 (23/217)	2.48	0.02	0.038 (24/637)	0.117 (34/290)	3.46	1.20E-05
HLADQA1_0501	0.563 (508/903)	0.42 (213/507)	0.57	8.00E-07	0.571 (152/266)	0.438 (95/217)	0.61	0.009	0.559 (356/637)	0.407 (118/290)	0.54	2.20E-05
HLADQB1_0402	0.048 (43/903)	0.122 (62/507)	2.76	1.30E-06	0.041 (11/266)	0.124 (27/217)	2.98	0.0039	0.05 (32/637)	0.121 (35/290)	2.62	0.00021
HLADQA1_0401	0.047 (42/903)	0.12 (61/507)	2.78	1.40E-06	0.041 (11/266)	0.12 (26/217)	2.81	0.0067	0.049 (31/637)	0.121 (35/290)	2.71	0.00014
HLADQB1_0201	0.291 (263/903)	0.189 (96/507)	0.52	2.50E-06	0.338 (90/266)	0.212 (46/217)	0.48	0.00076	0.272 (173/637)	0.172 (50/290)	0.51	0.00026
HLADRB1_0301	0.287 (259/903)	0.187 (95/507)	0.53	4.10E-06	0.327 (87/266)	0.207 (45/217)	0.5	0.0014	0.27 (172/637)	0.172 (50/290)	0.52	0.00032
HLADRB4_0101	0.137 (124/903)	0.063 (32/507)	0.41	2.20E-05	0.143 (38/266)	0.074 (16/217)	0.47	0.018	0.135 (86/637)	0.055 (16/290)	0.37	0.00041
HLADQA1_0301	0.285 (257/903)	0.402 (204/507)	1.65	2.50E-05	0.301 (80/266)	0.373 (81/217)	1.25	0.26	0.278 (177/637)	0.424 (123/290)	1.88	2.70E-05
HLAB_4402	0.14 (126/903)	0.235 (119/507)	1.82	3.20E-05	0.105 (28/266)	0.281 (61/217)	3.12	1.10E-05	0.154 (98/637)	0.2 (58/290)	1.37	0.089
HLADQB1_0302	0.172 (155/903)	0.264 (134/507)	1.72	6.30E-05	0.165 (44/266)	0.23 (50/217)	1.45	0.12	0.174 (111/637)	0.29 (84/290)	1.89	0.00018
HLADQA1_0102	0.37 (334/903)	0.268 (136/507)	0.62	9.10E-05	0.387 (103/266)	0.263 (57/217)	0.55	0.0031	0.363 (231/637)	0.272 (79/290)	0.65	0.0073
HLAC_0701	0.37 (334/903)	0.276 (140/507)	0.64	0.00021	0.406 (108/266)	0.313 (68/217)	0.69	0.057	0.355 (226/637)	0.248 (72/290)	0.58	0.00075
HLADRB5_0101	0.26 (235/903)	0.178 (90/507)	0.6	0.00027	0.305 (81/266)	0.175 (38/217)	0.44	4.00E-04	0.242 (154/637)	0.179 (52/290)	0.7	0.049
HLAB_0801	0.278 (251/903)	0.203 (103/507)	0.62	4.00E-04	0.297 (79/266)	0.212 (46/217)	0.59	0.017	0.27 (172/637)	0.197 (57/290)	0.63	0.0091
HLAB_3501	0.112 (101/903)	0.179 (91/507)	1.75	0.00043	0.094 (25/266)	0.147 (32/217)	1.61	0.1	0.119 (76/637)	0.203 (59/290)	1.95	0.00057
HLADRB1_1501	0.256 (231/903)	0.178 (90/507)	0.61	5.00E-04	0.305 (81/266)	0.175 (38/217)	0.44	4.00E-04	0.235 (150/637)	0.179 (52/290)	0.72	0.077
HLADRB3_0101	0.285 (257/903)	0.215 (109/507)	0.63	0.00052	0.308 (82/266)	0.221 (48/217)	0.58	0.012	0.275 (175/637)	0.21 (61/290)	0.65	0.013
HLAB_4403	0.066 (60/903)	0.022 (11/507)	0.31	0.00053	0.09 (24/266)	0.037 (8/217)	0.35	0.014	0.057 (36/637)	0.01 (3/290)	0.18	0.0048
HLADPB1_1301	0.095 (86/903)	0.041 (21/507)	0.42	0.00055	0.09 (24/266)	0.032 (7/217)	0.36	0.023	0.097 (62/637)	0.048 (14/290)	0.47	0.015
HLADRB1_0401	0.128 (116/903)	0.207 (105/507)	1.67	0.00076	0.135 (36/266)	0.226 (49/217)	1.59	0.067	0.126 (80/637)	0.193 (56/290)	1.61	0.014
HLADQB1_0602	0.244 (220/903)	0.178 (90/507)	0.65	0.0022	0.286 (76/266)	0.175 (38/217)	0.47	0.0013	0.226 (144/637)	0.179 (52/290)	0.76	0.13
HLADRB1_1104	0.144 (130/903)	0.077 (39/507)	0.55	0.0028	0.135 (36/266)	0.06 (13/217)	0.48	0.042	0.148 (94/637)	0.09 (26/290)	0.62	0.054
HLAA_0101	0.338 (305/903)	0.268 (136/507)	0.69	0.003	0.357 (95/266)	0.309 (67/217)	0.79	0.23	0.33 (210/637)	0.238 (69/290)	0.64	0.0064
HLADRB1_0102	0.019 (17/903)	0.041 (21/507)	2.51	0.0065	0.023 (6/266)	0.028 (6/217)	1.72	0.38	0.017 (11/637)	0.052 (15/290)	3.07	0.0067
HLAC_1601	0.049 (44/903)	0.018 (9/507)	0.36	0.0066	0.071 (19/266)	0.023 (5/217)	0.28	0.016	0.039 (25/637)	0.014 (4/290)	0.35	0.055
HLADPB1_1101	0.031 (28/903)	0.008 (4/507)	0.23	0.007	0.053 (14/266)	0.009 (2/217)	0.15	0.016	0.022 (14/637)	0.007 (2/290)	0.26	0.076
HLADRB1_1401	0.039 (35/903)	0.069 (35/507)	1.95	0.0073	0.03 (8/266)	0.06 (13/217)	2.34	0.073	0.042 (27/637)	0.076 (22/290)	1.93	0.029
HLADRB5_9901	0.966 (872/903)	0.99 (502/507)	3.62	0.0085	0.962 (256/266)	0.995 (216/217)	8.64	0.044	0.967 (616/637)	0.986 (286/290)	2.27	0.14
HLADRB4_0103	0.339 (306/903)	0.416 (211/507)	1.35	0.0086	0.365 (97/266)	0.406 (88/217)	1.11	0.59	0.328 (209/637)	0.424 (123/290)	1.47	0.0092
HLAA_2902	0.056 (51/903)	0.026 (13/507)	0.44	0.0097	0.056 (15/266)	0.037 (8/217)	0.63	0.31	0.057 (36/637)	0.017 (5/290)	0.29	0.011
HLADRB1_0404	0.064 (58/903)	0.107 (54/507)	1.68	0.0099	0.056 (15/266)	0.078 (17/217)	1.34	0.43	0.068 (43/637)	0.128 (37/290)	2.01	0.0039
HLADPA1_0201	0.324 (293/903)	0.26 (132/507)	0.73	0.011	0.353 (94/266)	0.235 (51/217)	0.57	0.0071	0.312 (199/637)	0.279 (81/290)	0.81	0.2
HLADQB1_0303	0.082 (74/903)	0.047 (24/507)	0.54	0.011	0.083 (22/266)	0.065 (14/217)	0.74	0.41	0.082 (52/637)	0.034 (10/290)	0.38	0.007
HLAB_1801	0.14 (126/903)	0.085 (43/507)	0.62	0.012	0.169 (45/266)	0.097 (21/217)	0.57	0.05	0.127 (81/637)	0.076 (22/290)	0.61	0.05
HLAC_0401	0.218 (197/903)	0.27 (137/507)	1.39	0.012	0.184 (49/266)	0.24 (52/217)	1.49	0.088	0.232 (148/637)	0.293 (85/290)	1.44	0.028
HLAC_0501	0.142 (128/903)	0.199 (101/507)	1.45	0.012	0.117 (31/266)	0.24 (52/217)	2.13	0.0031	0.152 (97/637)	0.169 (49/290)	1.14	0.51
HLADPB1_0301	0.206 (186/903)	0.262 (133/507)	1.35	0.021	0.207 (55/266)	0.226 (49/217)	1.03	0.88	0.206 (131/637)	0.29 (84/290)	1.53	0.0099
HLAC_1203	0.142 (128/903)	0.089 (45/507)	0.65	0.023	0.147 (39/266)	0.078 (17/217)	0.52	0.039	0.14 (89/637)	0.097 (28/290)	0.68	0.11
HLADRB3_9901	0.814 (735/903)	0.868 (440/507)	1.43	0.023	0.85 (226/266)	0.843 (183/217)	0.83	0.48	0.799 (509/637)	0.886 (257/290)	1.93	0.0021
HLAB_1402	0.051 (46/903)	0.077 (39/507)	1.68	0.024	0.041 (11/266)	0.055 (12/217)	1.6	0.29	0.055 (35/637)	0.093 (27/290)	1.81	0.031
HLADQB1_0503	0.042 (38/903)	0.067 (34/507)	1.74	0.024	0.038 (10/266)	0.06 (13/217)	1.95	0.13	0.044 (28/637)	0.072 (21/290)	1.77	0.06
HLADRB4_9901	0.919 (830/903)	0.951 (482/507)	1.72	0.025	0.91 (242/266)	0.945 (205/217)	1.72	0.15	0.923 (588/637)	0.955 (277/290)	1.85	0.059
HLAC_1402	0.021 (19/903)	0.039 (20/507)	1.92	0.047	0.038 (10/266)	0.023 (5/217)	0.68	0.5	0.014 (9/637)	0.052 (15/290)	3.77	0.0023

**HLAB_5101**	0.081 (73/903)	0.11 (56/507)	1.45	0.05	0.086 (23/266)	0.078 (17/217)	1.06	0.87	0.078 (50/637)	0.134 (39/290)	1.86	0.0071
**HLAC_0304**	0.125 (113/903)	0.168 (85/507)	1.35	0.056	0.128 (34/266)	0.147 (32/217)	1.08	0.77	0.124 (79/637)	0.183 (53/290)	1.54	0.029
**HLADPB1_0402**	0.219 (198/903)	0.258 (131/507)	1.28	0.056	0.192 (51/266)	0.286 (62/217)	1.69	0.018	0.231 (147/637)	0.238 (69/290)	1.11	0.53
**HLAA_0201**	0.452 (408/903)	0.511 (259/507)	1.23	0.065	0.444 (118/266)	0.507 (110/217)	1.29	0.18	0.455 (290/637)	0.514 (149/290)	1.2	0.21
**HLAC_0102**	0.064 (58/903)	0.089 (45/507)	1.46	0.069	0.053 (14/266)	0.097 (21/217)	1.95	0.068	0.069 (44/637)	0.083 (24/290)	1.25	0.4
**HLAB_4001**	0.091 (82/903)	0.124 (63/507)	1.38	0.072	0.102 (27/266)	0.124 (27/217)	1.14	0.65	0.086 (55/637)	0.124 (36/290)	1.49	0.084
**HLAC_1505**	0.014 (13/903)	0.002 (1/507)	0.15	0.074	0.015 (4/266)	0.005 (1/217)	0.49	0.53	0.014 (9/637)	0 (0/290)	NA	0.98
**HLAB_4901**	0.027 (24/903)	0.012 (6/507)	0.44	0.079	0.034 (9/266)	0.009 (2/217)	0.28	0.12	0.024 (15/637)	0.014 (4/290)	0.56	0.31
**HLAA_6801**	0.049 (44/903)	0.073 (37/507)	1.5	0.084	0.045 (12/266)	0.051 (11/217)	1.12	0.79	0.05 (32/637)	0.09 (26/290)	1.82	0.033
**HLAA_3101**	0.039 (35/903)	0.057 (29/507)	1.53	0.1	0.038 (10/266)	0.06 (13/217)	1.6	0.29	0.039 (25/637)	0.055 (16/290)	1.36	0.36
**HLADPB1_0201**	0.23 (208/903)	0.189 (96/507)	0.79	0.1	0.211 (56/266)	0.18 (39/217)	0.86	0.52	0.239 (152/637)	0.197 (57/290)	0.77	0.15
**HLADPB1_1001**	0.032 (29/903)	0.051 (26/507)	1.58	0.1	0.045 (12/266)	0.046 (10/217)	0.98	0.97	0.027 (17/637)	0.055 (16/290)	1.98	0.058
**HLAC_0802**	0.065 (59/903)	0.087 (44/507)	1.41	0.1	0.06 (16/266)	0.065 (14/217)	1.24	0.58	0.068 (43/637)	0.103 (30/290)	1.59	0.072
**HLAA_2301**	0.037 (33/903)	0.022 (11/507)	0.56	0.1	0.049 (13/266)	0.028 (6/217)	0.49	0.16	0.031 (20/637)	0.017 (5/290)	0.54	0.23
**HLADPB1_0101**	0.115 (104/903)	0.091 (46/507)	0.75	0.13	0.124 (33/266)	0.078 (17/217)	0.58	0.093	0.111 (71/637)	0.1 (29/290)	0.83	0.43
**HLADQB1_0502**	0.053 (48/903)	0.034 (17/507)	0.64	0.13	0.049 (13/266)	0.023 (5/217)	0.51	0.22	0.055 (35/637)	0.041 (12/290)	0.74	0.39
**HLAB_3503**	0.03 (27/903)	0.043 (22/507)	1.51	0.16	0.034 (9/266)	0.046 (10/217)	1.62	0.32	0.028 (18/637)	0.041 (12/290)	1.38	0.41
**HLADRB1_1001**	0.016 (14/903)	0.026 (13/507)	1.73	0.17	0.023 (6/266)	0.014 (3/217)	0.52	0.37	0.013 (8/637)	0.034 (10/290)	2.94	0.028
**HLAA_0301**	0.244 (220/903)	0.276 (140/507)	1.19	0.17	0.229 (61/266)	0.272 (59/217)	1.2	0.4	0.25 (159/637)	0.279 (81/290)	1.21	0.23
**HLAA_1101**	0.107 (97/903)	0.132 (67/507)	1.25	0.19	0.109 (29/266)	0.101 (22/217)	0.91	0.75	0.107 (68/637)	0.155 (45/290)	1.55	0.036
**HLADRB1_0407**	0.023 (21/903)	0.036 (18/507)	1.52	0.2	0.019 (5/266)	0.041 (9/217)	2.57	0.11	0.025 (16/637)	0.031 (9/290)	1.26	0.59
**HLAC_1502**	0.052 (47/903)	0.037 (19/507)	0.7	0.2	0.053 (14/266)	0.032 (7/217)	0.62	0.33	0.052 (33/637)	0.041 (12/290)	0.82	0.57
**HLAA_3301**	0.012 (11/903)	0.022 (11/507)	1.74	0.2	0.008 (2/266)	0.009 (2/217)	1.4	0.74	0.014 (9/637)	0.031 (9/290)	2.1	0.12
**HLADRB1_1601**	0.041 (37/903)	0.028 (14/507)	0.67	0.21	0.034 (9/266)	0.018 (4/217)	0.57	0.36	0.044 (28/637)	0.034 (10/290)	0.77	0.5
**HLAB_1501**	0.09 (81/903)	0.112 (57/507)	1.26	0.21	0.083 (22/266)	0.106 (23/217)	1.29	0.43	0.093 (59/637)	0.117 (34/290)	1.31	0.24
**HLADRB1_0402**	0.039 (35/903)	0.02 (10/507)	0.64	0.24	0.038 (10/266)	0.009 (2/217)	0.28	0.12	0.039 (25/637)	0.028 (8/290)	0.8	0.62
**HLADRB1_0901**	0.02 (18/903)	0.012 (6/507)	0.57	0.24	0.026 (7/266)	0.014 (3/217)	0.46	0.28	0.017 (11/637)	0.01 (3/290)	0.52	0.33
**HLAA_3001**	0.018 (16/903)	0.024 (12/507)	1.58	0.24	0.019 (5/266)	0.032 (7/217)	2.4	0.16	0.017 (11/637)	0.017 (5/290)	1.17	0.78
**HLAB_0702**	0.236 (213/903)	0.215 (109/507)	0.85	0.25	0.274 (73/266)	0.244 (53/217)	0.83	0.38	0.22 (140/637)	0.193 (56/290)	0.81	0.25
**HLAB_3906**	0.016 (14/903)	0.026 (13/507)	1.56	0.26	0.023 (6/266)	0.018 (4/217)	0.68	0.56	0.013 (8/637)	0.031 (9/290)	2.85	0.036
**HLAA_0302**	0.013 (12/903)	0.006 (3/507)	0.48	0.26	0.015 (4/266)	0 (0/217)	NA	0.98	0.013 (8/637)	0.01 (3/290)	0.87	0.84
**HLADRB3_0202**	0.4 (361/903)	0.349 (177/507)	0.88	0.27	0.342 (91/266)	0.355 (77/217)	1.23	0.32	0.424 (270/637)	0.345 (100/290)	0.76	0.07
**HLAA_2601**	0.086 (78/903)	0.063 (32/507)	0.78	0.27	0.064 (17/266)	0.06 (13/217)	1.03	0.94	0.096 (61/637)	0.066 (19/290)	0.74	0.27
**HLAC_0704**	0.027 (24/903)	0.037 (19/507)	1.41	0.28	0.011 (3/266)	0.037 (8/217)	3.61	0.066	0.033 (21/637)	0.038 (11/290)	1.12	0.78
**HLADRB1_1301**	0.065 (59/903)	0.081 (41/507)	1.25	0.3	0.038 (10/266)	0.088 (19/217)	2.25	0.049	0.077 (49/637)	0.076 (22/290)	1.04	0.88
**HLAB_5801**	0.013 (12/903)	0.02 (10/507)	1.57	0.3	0.011 (3/266)	0.023 (5/217)	2.37	0.26	0.014 (9/637)	0.017 (5/290)	1.18	0.78
**HLAB_3508**	0.02 (18/903)	0.012 (6/507)	0.61	0.31	0.023 (6/266)	0.009 (2/217)	0.43	0.31	0.019 (12/637)	0.014 (4/290)	0.67	0.5
**HLAB_3701**	0.027 (24/903)	0.02 (10/507)	0.68	0.32	0.038 (10/266)	0.018 (4/217)	0.39	0.13	0.022 (14/637)	0.021 (6/290)	0.94	0.9
**HLAB_3801**	0.048 (43/903)	0.032 (16/507)	0.74	0.32	0.03 (8/266)	0.028 (6/217)	0.98	0.97	0.055 (35/637)	0.034 (10/290)	0.64	0.25
**HLAB_5601**	0.021 (19/903)	0.028 (14/507)	1.42	0.33	0.019 (5/266)	0.032 (7/217)	1.55	0.47	0.022 (14/637)	0.024 (7/290)	1.16	0.76
**HLAA_0205**	0.018 (16/903)	0.024 (12/507)	1.47	0.33	0.019 (5/266)	0.014 (3/217)	0.78	0.75	0.017 (11/637)	0.031 (9/290)	1.81	0.21
**HLADPB1_0901**	0.016 (14/903)	0.022 (11/507)	1.49	0.33	0.026 (7/266)	0.023 (5/217)	0.99	0.99	0.011 (7/637)	0.021 (6/290)	1.91	0.26
**HLAC_1701**	0.028 (25/903)	0.018 (9/507)	0.68	0.34	0.019 (5/266)	0.018 (4/217)	0.95	0.94	0.031 (20/637)	0.017 (5/290)	0.56	0.27
**HLADQB1_0604**	0.054 (49/903)	0.043 (22/507)	0.78	0.34	0.038 (10/266)	0.041 (9/217)	1.31	0.58	0.061 (39/637)	0.045 (13/290)	0.65	0.19
**HLADRB1_0804**	0.011 (10/903)	0.006 (3/507)	0.53	0.35	0 (0/266)	0.014 (3/217)	NA	0.98	0.016 (10/637)	0 (0/290)	NA	0.97
**HLADRB1_1201**	0.027 (24/903)	0.018 (9/507)	0.69	0.35	0.015 (4/266)	0.032 (7/217)	2.39	0.18	0.031 (20/637)	0.007 (2/290)	0.2	0.033
**HLAB_5201**	0.018 (16/903)	0.012 (6/507)	0.64	0.36	0.019 (5/266)	0.014 (3/217)	0.65	0.57	0.017 (11/637)	0.01 (3/290)	0.57	0.4
**HLAB_5301**	0.011 (10/903)	0.006 (3/507)	0.54	0.36	0.004 (1/266)	0.005 (1/217)	1.73	0.71	0.014 (9/637)	0.007 (2/290)	0.49	0.37
**HLADPB1_1701**	0.024 (22/903)	0.018 (9/507)	0.69	0.36	0.026 (7/266)	0.023 (5/217)	0.86	0.79	0.024 (15/637)	0.014 (4/290)	0.55	0.29
**HLADQB1_0601**	0.013 (12/903)	0.008 (4/507)	0.59	0.37	0.015 (4/266)	0.014 (3/217)	0.95	0.95	0.013 (8/637)	0.003 (1/290)	0.29	0.24
**HLADPB1_0202**	0.011 (10/903)	0.006 (3/507)	0.56	0.39	0.004 (1/266)	0.005 (1/217)	1.91	0.65	0.014 (9/637)	0.007 (2/290)	0.53	0.43
**HLAC_0602**	0.134 (121/903)	0.12 (61/507)	0.86	0.39	0.132 (35/266)	0.124 (27/217)	0.91	0.73	0.135 (86/637)	0.117 (34/290)	0.86	0.48
**HLADPB1_0501**	0.038 (34/903)	0.047 (24/507)	1.27	0.39	0.038 (10/266)	0.06 (13/217)	1.58	0.3	0.038 (24/637)	0.038 (11/290)	1.01	0.97
**HLADRB1_1502**	0.013 (12/903)	0.008 (4/507)	0.61	0.39	0.011 (3/266)	0.014 (3/217)	1.26	0.79	0.014 (9/637)	0.003 (1/290)	0.25	0.19
**HLAB_3502**	0.042 (38/903)	0.028 (14/507)	0.76	0.4	0.026 (7/266)	0.023 (5/217)	1.24	0.73	0.049 (31/637)	0.031 (9/290)	0.7	0.37

**HLADRB1_1302**	0.08 (72/903)	0.067 (34/507)	0.83	0.41	0.053 (14/266)	0.074 (16/217)	1.66	0.2	0.091 (58/637)	0.062 (18/290)	0.62	0.089
**HLAB_4102**	0.012 (11/903)	0.008 (4/507)	0.62	0.42	0.004 (1/266)	0 (0/217)	NA	0.98	0.016 (10/637)	0.014 (4/290)	0.81	0.74
**HLAC_1202**	0.017 (15/903)	0.012 (6/507)	0.68	0.43	0.019 (5/266)	0.014 (3/217)	0.65	0.57	0.016 (10/637)	0.01 (3/290)	0.63	0.49
**HLADQA1_0103**	0.078 (70/903)	0.089 (45/507)	1.17	0.43	0.045 (12/266)	0.101 (22/217)	2.22	0.035	0.091 (58/637)	0.079 (23/290)	0.92	0.74
**HLADRB3_0301**	0.076 (69/903)	0.065 (33/507)	0.84	0.43	0.049 (13/266)	0.074 (16/217)	1.78	0.15	0.088 (56/637)	0.059 (17/290)	0.6	0.081
**HLADRB1_0103**	0.022 (20/903)	0.032 (16/507)	1.29	0.46	0.019 (5/266)	0.037 (8/217)	1.98	0.25	0.024 (15/637)	0.028 (8/290)	1.03	0.95
**HLAA_6601**	0.013 (12/903)	0.01 (5/507)	0.68	0.47	0.008 (2/266)	0.009 (2/217)	1.11	0.92	0.016 (10/637)	0.01 (3/290)	0.56	0.39
**HLAB_5001**	0.014 (13/903)	0.02 (10/507)	1.36	0.47	0.019 (5/266)	0.028 (6/217)	1.37	0.61	0.013 (8/637)	0.014 (4/290)	1.01	0.99
**HLAB_5501**	0.033 (30/903)	0.026 (13/507)	0.78	0.48	0.03 (8/266)	0.032 (7/217)	1.28	0.65	0.035 (22/637)	0.021 (6/290)	0.56	0.22
**HLADQB1_0301**	0.419 (378/903)	0.385 (195/507)	0.92	0.49	0.398 (106/266)	0.396 (86/217)	1.09	0.67	0.427 (272/637)	0.376 (109/290)	0.85	0.27
**HLADRB5_0202**	0.041 (37/903)	0.034 (17/507)	0.84	0.57	0.03 (8/266)	0.023 (5/217)	0.86	0.8	0.046 (29/637)	0.041 (12/290)	0.92	0.82
**HLAB_2705**	0.074 (67/903)	0.085 (43/507)	1.12	0.59	0.06 (16/266)	0.074 (16/217)	1.17	0.68	0.08 (51/637)	0.093 (27/290)	1.18	0.52
**HLAB_4101**	0.012 (11/903)	0.008 (4/507)	0.73	0.6	0.011 (3/266)	0.014 (3/217)	1.09	0.92	0.013 (8/637)	0.003 (1/290)	0.33	0.3
**HLADPA1_0103**	0.961 (868/903)	0.968 (491/507)	1.16	0.63	0.959 (255/266)	0.972 (211/217)	1.43	0.5	0.962 (613/637)	0.966 (280/290)	1.06	0.87
**HLAA_3002**	0.018 (16/903)	0.014 (7/507)	0.81	0.66	0.015 (4/266)	0.005 (1/217)	0.28	0.27	0.019 (12/637)	0.021 (6/290)	1.2	0.72
**HLAB_5701**	0.053 (48/903)	0.049 (25/507)	0.9	0.69	0.045 (12/266)	0.051 (11/217)	1.19	0.69	0.057 (36/637)	0.048 (14/290)	0.86	0.64
**HLADQB1_0603**	0.073 (66/903)	0.079 (40/507)	1.09	0.69	0.041 (11/266)	0.083 (18/217)	1.87	0.12	0.086 (55/637)	0.076 (22/290)	0.93	0.78
**HLAB_4501**	0.012 (11/903)	0.01 (5/507)	0.81	0.69	0.008 (2/266)	0.009 (2/217)	1.34	0.77	0.014 (9/637)	0.01 (3/290)	0.71	0.61
**HLADPB1_0401**	0.66 (596/903)	0.677 (343/507)	1.05	0.7	0.643 (171/266)	0.728 (158/217)	1.45	0.071	0.667 (425/637)	0.638 (185/290)	0.88	0.41
**HLAA_2501**	0.044 (40/903)	0.039 (20/507)	0.9	0.71	0.071 (19/266)	0.041 (9/217)	0.55	0.16	0.033 (21/637)	0.038 (11/290)	1.17	0.69
**HLAA_2402**	0.189 (171/903)	0.179 (91/507)	0.95	0.71	0.218 (58/266)	0.171 (37/217)	0.75	0.23	0.177 (113/637)	0.186 (54/290)	1.03	0.86
**HLAC_0702**	0.259 (234/903)	0.256 (130/507)	0.95	0.71	0.289 (77/266)	0.286 (62/217)	0.94	0.76	0.246 (157/637)	0.234 (68/290)	0.92	0.61
**HLAC_0303**	0.09 (81/903)	0.085 (43/507)	0.93	0.72	0.075 (20/266)	0.101 (22/217)	1.48	0.24	0.096 (61/637)	0.072 (21/290)	0.71	0.21
**HLAB_1401**	0.014 (13/903)	0.014 (7/507)	0.86	0.75	0.019 (5/266)	0.009 (2/217)	0.52	0.45	0.013 (8/637)	0.017 (5/290)	1.23	0.72
**HLAA_6802**	0.014 (13/903)	0.016 (8/507)	1.15	0.76	0.004 (1/266)	0.018 (4/217)	5.06	0.15	0.019 (12/637)	0.014 (4/290)	0.71	0.57
**HLADRB1_1303**	0.016 (14/903)	0.018 (9/507)	1.13	0.78	0.011 (3/266)	0.018 (4/217)	1.75	0.49	0.017 (11/637)	0.017 (5/290)	1.04	0.94
**HLADQB1_0609**	0.022 (20/903)	0.024 (12/507)	1.1	0.79	0.015 (4/266)	0.032 (7/217)	2.37	0.18	0.025 (16/637)	0.017 (5/290)	0.66	0.44
**HLADRB1_0405**	0.011 (10/903)	0.01 (5/507)	0.86	0.79	0.019 (5/266)	0.014 (3/217)	0.67	0.6	0.008 (5/637)	0.007 (2/290)	0.81	0.8
**HLAC_0202**	0.079 (71/903)	0.085 (43/507)	1.05	0.81	0.071 (19/266)	0.051 (11/217)	0.67	0.31	0.082 (52/637)	0.11 (32/290)	1.36	0.2
**HLADPA1_0202**	0.052 (47/903)	0.049 (25/507)	0.94	0.81	0.053 (14/266)	0.051 (11/217)	0.88	0.77	0.052 (33/637)	0.048 (14/290)	0.95	0.89
**HLAB_3901**	0.027 (24/903)	0.028 (14/507)	1.08	0.82	0.026 (7/266)	0.023 (5/217)	0.87	0.81	0.027 (17/637)	0.031 (9/290)	1.12	0.79
**HLADPB1_1401**	0.038 (34/903)	0.039 (20/507)	1.06	0.83	0.026 (7/266)	0.028 (6/217)	1.21	0.74	0.042 (27/637)	0.048 (14/290)	1.14	0.71
**HLADPB1_0601**	0.035 (32/903)	0.034 (17/507)	0.94	0.84	0.045 (12/266)	0.018 (4/217)	0.41	0.13	0.031 (20/637)	0.045 (13/290)	1.54	0.24
**HLAA_3201**	0.059 (53/903)	0.057 (29/507)	0.96	0.86	0.049 (13/266)	0.055 (12/217)	1.22	0.64	0.063 (40/637)	0.059 (17/290)	0.89	0.7
**HLAB_1302**	0.027 (24/903)	0.024 (12/507)	0.97	0.94	0.023 (6/266)	0.023 (5/217)	1.23	0.75	0.028 (18/637)	0.024 (7/290)	0.92	0.85
**HLAB_4002**	0.025 (23/903)	0.026 (13/507)	1	0.99	0.015 (4/266)	0.009 (2/217)	0.63	0.6	0.03 (19/637)	0.038 (11/290)	1.33	0.46
**HLADRB1_1101**	0.136 (123/903)	0.132 (67/507)	1	0.99	0.132 (35/266)	0.138 (30/217)	1.13	0.66	0.138 (88/637)	0.128 (37/290)	0.92	0.68

Only alleles seen in >=10 ACA- SSc patients in the meta-analysis cohort are shown. Rare alleles for which odds ratios could not be accurately computed are assigned OR = NA. CO = control, OR = odds ratio, NA = not applicable.

Supplementary Table 8: Classical HLA allele frequency differences between ATA+ and ATA- SSc in all three study cohorts.

	ATA+ vs -											
	Meta-analysis Cohort				Cohort 1				Cohort 2			
**HLA Allele**	**ATA- Prop.(Count)**	**ATA+ Prop.(Count)**	**OR**	**p**	**ATA- Prop.(Count)**	**ATA+ Prop.(Count)**	**OR**	**p**	**ATA- Prop.(Count)**	**ATA+ Prop.(Count)**	**OR**	**p**
**HLADPB1_1301**	0.041 (48/1170)	0.25 (60/240)	7.85	**9.30E-22**	0.035 (14/404)	0.228 (18/79)	8.54	**1.20E-07**	0.044 (34/766)	0.261 (42/161)	8.39	**6.80E-16**
**HLADRB1_1104**	0.083 (97/1170)	0.3 (72/240)	4.13	**9.70E-14**	0.069 (28/404)	0.266 (21/79)	4.93	9.00E-06	0.09 (69/766)	0.317 (51/161)	3.9	**3.30E-09**
**HLADQA1_0101**	0.339 (397/1170)	0.133 (32/240)	0.3	**2.40E-09**	0.347 (140/404)	0.114 (9/79)	0.23	0.00011	0.336 (257/766)	0.143 (23/161)	0.33	**4.20E-06**
**HLADPA1_0201**	0.271 (317/1170)	0.454 (109/240)	2.33	**1.00E-08**	0.26 (105/404)	0.519 (41/79)	3.26	5.90E-06	0.277 (212/766)	0.422 (68/161)	2.17	2.80E-05
**HLADRB1_1501**	0.203 (237/1170)	0.35 (84/240)	2.4	**4.20E-08**	0.223 (90/404)	0.367 (29/79)	2.25	0.003	0.192 (147/766)	0.342 (55/161)	2.37	1.60E-05
**HLADRB5_0101**	0.205 (240/1170)	0.354 (85/240)	2.38	**5.00E-08**	0.223 (90/404)	0.367 (29/79)	2.25	0.003	0.196 (150/766)	0.348 (56/161)	2.33	1.80E-05
**HLADQB1_0602**	0.197 (231/1170)	0.329 (79/240)	2.33	**2.20E-07**	0.21 (85/404)	0.367 (29/79)	2.55	0.00078	0.191 (146/766)	0.311 (50/161)	2.11	0.00024
**HLADQB1_0501**	0.292 (342/1170)	0.133 (32/240)	0.38	**1.70E-06**	0.302 (122/404)	0.114 (9/79)	0.3	0.0013	0.287 (220/766)	0.143 (23/161)	0.42	3.00E-04
**HLADQB1_0301**	0.374 (438/1170)	0.562 (135/240)	1.94	6.50E-06	0.374 (151/404)	0.519 (41/79)	1.67	0.046	0.375 (287/766)	0.584 (94/161)	2.11	4.50E-05
**HLADQB1_0303**	0.056 (65/1170)	0.138 (33/240)	2.81	7.60E-06	0.064 (26/404)	0.127 (10/79)	2.13	0.059	0.051 (39/766)	0.143 (23/161)	3.26	3.90E-05
**HLADQA1_0102**	0.313 (366/1170)	0.438 (105/240)	1.82	4.90E-05	0.312 (126/404)	0.443 (35/79)	1.78	0.024	0.313 (240/766)	0.435 (70/161)	1.79	0.0015
**HLAB_1801**	0.102 (119/1170)	0.208 (50/240)	2.09	1.00E-04	0.121 (49/404)	0.215 (17/79)	1.78	0.076	0.091 (70/766)	0.205 (33/161)	2.26	0.00066
**HLADQA1_0301**	0.35 (410/1170)	0.212 (51/240)	0.52	0.00011	0.354 (143/404)	0.228 (18/79)	0.58	0.065	0.349 (267/766)	0.205 (33/161)	0.49	0.00084
**HLADRB1_0101**	0.226 (265/1170)	0.104 (25/240)	0.42	0.00011	0.23 (93/404)	0.114 (9/79)	0.45	0.033	0.225 (172/766)	0.099 (16/161)	0.4	0.0012
**HLADRB1_0301**	0.274 (321/1170)	0.138 (33/240)	0.46	0.00014	0.277 (112/404)	0.253 (20/79)	0.96	0.88	0.273 (209/766)	0.081 (13/161)	0.26	1.00E-05
**HLADRB3_0202**	0.351 (411/1170)	0.525 (126/240)	1.76	0.00016	0.329 (133/404)	0.43 (34/79)	1.33	0.28	0.363 (278/766)	0.571 (92/161)	1.98	0.00022
**HLADQB1_0201**	0.277 (324/1170)	0.146 (35/240)	0.49	0.00031	0.287 (116/404)	0.253 (20/79)	0.92	0.77	0.272 (208/766)	0.093 (15/161)	0.31	4.10E-05
**HLADRB1_0701**	0.138 (161/1170)	0.225 (54/240)	1.87	0.00046	0.139 (56/404)	0.266 (21/79)	2.24	0.0067	0.137 (105/766)	0.205 (33/161)	1.71	0.018
**HLADQA1_0201**	0.138 (162/1170)	0.225 (54/240)	1.86	0.00051	0.141 (57/404)	0.266 (21/79)	2.21	0.0077	0.137 (105/766)	0.205 (33/161)	1.71	0.018
**HLAB_0702**	0.215 (252/1170)	0.296 (71/240)	1.76	0.00057	0.26 (105/404)	0.278 (22/79)	1.2	0.53	0.192 (147/766)	0.304 (49/161)	2.17	0.00015
**HLADQA1_0501**	0.488 (571/1170)	0.625 (150/240)	1.63	0.00092	0.488 (197/404)	0.633 (50/79)	1.73	0.035	0.488 (374/766)	0.621 (100/161)	1.61	0.0092
**HLAB_3508**	0.011 (13/1170)	0.046 (11/240)	3.74	0.0021	0.012 (5/404)	0.038 (3/79)	2.72	0.19	0.01 (8/766)	0.05 (8/161)	5.07	0.0021
**HLADQB1_0302**	0.22 (257/1170)	0.133 (32/240)	0.53	0.0021	0.205 (83/404)	0.139 (11/79)	0.65	0.23	0.227 (174/766)	0.13 (21/161)	0.5	0.0065
**HLADPB1_0301**	0.244 (285/1170)	0.146 (35/240)	0.55	0.0023	0.228 (92/404)	0.165 (13/79)	0.71	0.31	0.252 (193/766)	0.137 (22/161)	0.49	0.0044
**HLAC_0802**	0.082 (96/1170)	0.029 (7/240)	0.31	0.0036	0.072 (29/404)	0.013 (1/79)	0.14	0.059	0.087 (67/766)	0.037 (6/161)	0.37	0.026
**HLAA_2402**	0.171 (200/1170)	0.258 (62/240)	1.63	0.0038	0.186 (75/404)	0.253 (20/79)	1.46	0.2	0.163 (125/766)	0.261 (42/161)	1.84	0.0039
**HLAC_0702**	0.248 (290/1170)	0.312 (75/240)	1.58	0.0047	0.292 (118/404)	0.278 (22/79)	1.02	0.94	0.225 (172/766)	0.329 (53/161)	1.97	0.00063
**HLAB_3501**	0.148 (173/1170)	0.079 (19/240)	0.49	0.0048	0.124 (50/404)	0.089 (7/79)	0.7	0.41	0.161 (123/766)	0.075 (12/161)	0.41	0.0053
**HLADRB1_0401**	0.171 (200/1170)	0.083 (20/240)	0.5	0.0058	0.186 (75/404)	0.114 (9/79)	0.65	0.26	0.163 (125/766)	0.068 (11/161)	0.43	0.012
**HLAB_1402**	0.068 (79/1170)	0.025 (6/240)	0.31	0.0073	0.054 (22/404)	0.013 (1/79)	0.18	0.11	0.074 (57/766)	0.031 (5/161)	0.35	0.03
**HLAB_5101**	0.101 (118/1170)	0.05 (12/240)	0.43	0.0074	0.097 (39/404)	0.025 (2/79)	0.19	0.026	0.103 (79/766)	0.062 (10/161)	0.54	0.083
**HLADRB5_9901**	0.979 (1146/1170)	0.95 (228/240)	0.39	0.011	0.988 (399/404)	0.924 (73/79)	0.14	0.0024	0.975 (747/766)	0.963 (155/161)	0.73	0.52
**HLADRB1_1401**	0.056 (66/1170)	0.017 (4/240)	0.26	0.011	0.052 (21/404)	0 (0/79)	NA	0.98	0.059 (45/766)	0.025 (4/161)	0.38	0.076
**HLAB_5701**	0.045 (53/1170)	0.083 (20/240)	2	0.012	0.037 (15/404)	0.101 (8/79)	3.03	0.017	0.05 (38/766)	0.075 (12/161)	1.58	0.19
**HLADRB3_0101**	0.278 (325/1170)	0.175 (42/240)	0.63	0.012	0.272 (110/404)	0.266 (21/79)	1.07	0.82	0.281 (215/766)	0.13 (21/161)	0.45	0.0015
**HLAC_1203**	0.108 (126/1170)	0.192 (46/240)	1.63	0.014	0.104 (42/404)	0.165 (13/79)	1.49	0.26	0.11 (84/766)	0.205 (33/161)	1.73	0.024
**HLAB_0801**	0.267 (312/1170)	0.171 (41/240)	0.63	0.015	0.272 (110/404)	0.177 (14/79)	0.61	0.13	0.264 (202/766)	0.168 (27/161)	0.65	0.06
**HLADQB1_0503**	0.057 (67/1170)	0.021 (5/240)	0.33	0.018	0.057 (23/404)	0 (0/79)	NA	0.98	0.057 (44/766)	0.031 (5/161)	0.51	0.16
**HLADRB1_0102**	0.031 (36/1170)	0.008 (2/240)	0.22	0.041	0.027 (11/404)	0.013 (1/79)	0.32	0.29	0.033 (25/766)	0.006 (1/161)	0.16	0.077
**HLADPB1_0401**	0.678 (793/1170)	0.604 (145/240)	0.75	0.049	0.708 (286/404)	0.532 (42/79)	0.47	0.0029	0.662 (507/766)	0.64 (103/161)	0.9	0.58
**HLADPB1_0601**	0.031 (36/1170)	0.054 (13/240)	1.92	0.053	0.02 (8/404)	0.101 (8/79)	7.03	0.00031	0.037 (28/766)	0.031 (5/161)	0.83	0.71
**HLADRB1_0404**	0.087 (102/1170)	0.046 (11/240)	0.53	0.054	0.064 (26/404)	0.089 (7/79)	1.58	0.31	0.099 (76/766)	0.025 (4/161)	0.24	0.0064
**HLAC_1402**	0.032 (37/1170)	0.008 (2/240)	0.25	0.06	0.035 (14/404)	0.013 (1/79)	0.32	0.28	0.03 (23/766)	0.006 (1/161)	0.2	0.12
**HLAA_1101**	0.125 (146/1170)	0.083 (20/240)	0.64	0.078	0.109 (44/404)	0.114 (9/79)	1.05	0.9	0.133 (102/766)	0.068 (11/161)	0.48	0.027
**HLAA_2902**	0.041 (48/1170)	0.067 (16/240)	1.7	0.08	0.045 (18/404)	0.063 (5/79)	1.53	0.42	0.039 (30/766)	0.068 (11/161)	1.88	0.095
**HLAB_3502**	0.03 (35/1170)	0.071 (17/240)	1.76	0.08	0.017 (7/404)	0.063 (5/79)	2.75	0.11	0.037 (28/766)	0.075 (12/161)	1.46	0.32
**HLADRB1_0407**	0.032 (37/1170)	0.008 (2/240)	0.28	0.085	0.032 (13/404)	0.013 (1/79)	0.36	0.34	0.031 (24/766)	0.006 (1/161)	0.22	0.14
**HLADRB4_0103**	0.378 (442/1170)	0.312 (75/240)	0.77	0.085	0.386 (156/404)	0.367 (29/79)	0.98	0.93	0.373 (286/766)	0.286 (46/161)	0.69	0.052
**HLAB_5501**	0.034 (40/1170)	0.012 (3/240)	0.35	0.086	0.032 (13/404)	0.025 (2/79)	0.69	0.63	0.035 (27/766)	0.006 (1/161)	0.18	0.093

**HLADRB1_0405**	0.009 (10/1170)	0.021 (5/240)	2.55	0.097	0.017 (7/404)	0.013 (1/79)	0.73	0.78	0.004 (3/766)	0.025 (4/161)	8.76	0.0058
**HLADRB1_0801**	0.071 (83/1170)	0.038 (9/240)	0.55	0.097	0.084 (34/404)	0 (0/79)	NA	0.98	0.064 (49/766)	0.056 (9/161)	1.01	0.97
**HLAC_0602**	0.123 (144/1170)	0.158 (38/240)	1.39	0.099	0.111 (45/404)	0.215 (17/79)	2.29	0.011	0.129 (99/766)	0.13 (21/161)	1.03	0.92
**HLADPB1_0501**	0.045 (53/1170)	0.021 (5/240)	0.46	0.1	0.052 (21/404)	0.025 (2/79)	0.44	0.28	0.042 (32/766)	0.019 (3/161)	0.48	0.23
**HLAA_2301**	0.028 (33/1170)	0.046 (11/240)	1.8	0.1	0.032 (13/404)	0.076 (6/79)	2.72	0.057	0.026 (20/766)	0.031 (5/161)	1.22	0.71
**HLADRB1_1601**	0.039 (46/1170)	0.021 (5/240)	0.46	0.11	0.027 (11/404)	0.025 (2/79)	0.8	0.78	0.046 (35/766)	0.019 (3/161)	0.34	0.082
**HLAA_0201**	0.486 (569/1170)	0.412 (99/240)	0.79	0.12	0.488 (197/404)	0.405 (32/79)	0.74	0.22	0.486 (372/766)	0.416 (67/161)	0.85	0.38
**HLAC_1202**	0.013 (15/1170)	0.025 (6/240)	2.18	0.12	0.017 (7/404)	0.013 (1/79)	0.78	0.82	0.01 (8/766)	0.031 (5/161)	3.61	0.033
**HLADRB1_1001**	0.021 (25/1170)	0.008 (2/240)	0.33	0.13	0.022 (9/404)	0 (0/79)	NA	0.98	0.021 (16/766)	0.012 (2/161)	0.48	0.34
**HLAC_0304**	0.149 (174/1170)	0.1 (24/240)	0.71	0.13	0.149 (60/404)	0.076 (6/79)	0.51	0.14	0.149 (114/766)	0.112 (18/161)	0.83	0.49
**HLADRB1_1101**	0.126 (148/1170)	0.175 (42/240)	1.33	0.14	0.131 (53/404)	0.152 (12/79)	1.02	0.95	0.124 (95/766)	0.186 (30/161)	1.5	0.085
**HLADRB5_0202**	0.041 (48/1170)	0.025 (6/240)	0.53	0.15	0.027 (11/404)	0.025 (2/79)	0.8	0.78	0.048 (37/766)	0.025 (4/161)	0.44	0.13
**HLADPB1_1001**	0.036 (42/1170)	0.054 (13/240)	1.61	0.15	0.035 (14/404)	0.101 (8/79)	3.29	0.012	0.037 (28/766)	0.031 (5/161)	0.97	0.95
**HLAC_0501**	0.17 (199/1170)	0.121 (29/240)	0.73	0.15	0.178 (72/404)	0.127 (10/79)	0.74	0.41	0.166 (127/766)	0.118 (19/161)	0.73	0.24
**HLAA_2501**	0.039 (46/1170)	0.058 (14/240)	1.57	0.16	0.054 (22/404)	0.076 (6/79)	1.47	0.43	0.031 (24/766)	0.05 (8/161)	1.64	0.24
**HLAB_5201**	0.014 (16/1170)	0.025 (6/240)	2.02	0.16	0.017 (7/404)	0.013 (1/79)	0.78	0.82	0.012 (9/766)	0.031 (5/161)	3.12	0.052
**HLADRB1_1201**	0.021 (24/1170)	0.038 (9/240)	1.77	0.16	0.027 (11/404)	0 (0/79)	NA	0.98	0.017 (13/766)	0.056 (9/161)	3.69	0.0046
**HLADPB1_0101**	0.113 (132/1170)	0.075 (18/240)	0.7	0.17	0.104 (42/404)	0.101 (8/79)	1.03	0.94	0.117 (90/766)	0.062 (10/161)	0.61	0.16
**HLADRB1_0402**	0.032 (37/1170)	0.033 (8/240)	0.57	0.19	0.027 (11/404)	0.013 (1/79)	0.3	0.26	0.034 (26/766)	0.043 (7/161)	0.68	0.42
**HLAB_3901**	0.029 (34/1170)	0.017 (4/240)	0.52	0.23	0.027 (11/404)	0.013 (1/79)	0.4	0.4	0.03 (23/766)	0.019 (3/161)	0.65	0.5
**HLAB_1401**	0.016 (19/1170)	0.004 (1/240)	0.29	0.23	0.017 (7/404)	0 (0/79)	NA	0.99	0.016 (12/766)	0.006 (1/161)	0.46	0.47
**HLAA_3002**	0.018 (21/1170)	0.008 (2/240)	0.41	0.24	0.007 (3/404)	0.025 (2/79)	3.24	0.22	0.023 (18/766)	0 (0/161)	0	0.98
**HLADQA1_0103**	0.078 (91/1170)	0.1 (24/240)	1.33	0.25	0.072 (29/404)	0.063 (5/79)	0.92	0.87	0.081 (62/766)	0.118 (19/161)	1.47	0.18
**HLADQB1_0609**	0.025 (29/1170)	0.012 (3/240)	0.5	0.26	0.027 (11/404)	0 (0/79)	NA	0.98	0.023 (18/766)	0.019 (3/161)	0.83	0.76
**HLADRB1_0901**	0.019 (22/1170)	0.008 (2/240)	0.43	0.26	0.025 (10/404)	0 (0/79)	NA	0.98	0.016 (12/766)	0.012 (2/161)	0.82	0.8
**HLAC_0102**	0.076 (89/1170)	0.058 (14/240)	0.71	0.26	0.079 (32/404)	0.038 (3/79)	0.45	0.2	0.074 (57/766)	0.068 (11/161)	0.85	0.65
**HLAA_6802**	0.016 (19/1170)	0.008 (2/240)	0.44	0.27	0.01 (4/404)	0.013 (1/79)	1.38	0.78	0.02 (15/766)	0.006 (1/161)	0.25	0.18
**HLADQB1_0402**	0.079 (93/1170)	0.054 (13/240)	0.71	0.27	0.097 (39/404)	0 (0/79)	NA	0.98	0.07 (54/766)	0.081 (13/161)	1.32	0.4
**HLAA_2601**	0.079 (92/1170)	0.075 (18/240)	0.74	0.27	0.062 (25/404)	0.063 (5/79)	0.92	0.88	0.087 (67/766)	0.081 (13/161)	0.66	0.21
**HLAB_4402**	0.18 (211/1170)	0.138 (33/240)	0.8	0.29	0.198 (80/404)	0.101 (8/79)	0.5	0.085	0.171 (131/766)	0.155 (25/161)	0.98	0.92
**HLADPA1_0103**	0.967 (1131/1170)	0.95 (228/240)	0.7	0.29	0.968 (391/404)	0.949 (75/79)	0.68	0.52	0.966 (740/766)	0.95 (153/161)	0.67	0.34
**HLADRB1_0804**	0.009 (10/1170)	0.017 (4/240)	1.89	0.3	0.01 (4/404)	0 (0/79)	NA	0.98	0.008 (6/766)	0.025 (4/161)	3.44	0.069
**HLAB_5801**	0.015 (17/1170)	0.025 (6/240)	1.63	0.32	0.017 (7/404)	0.025 (2/79)	1.35	0.71	0.013 (10/766)	0.025 (4/161)	1.88	0.31
**HLADRB1_1301**	0.068 (80/1170)	0.083 (20/240)	1.29	0.33	0.059 (24/404)	0.063 (5/79)	1.13	0.82	0.073 (56/766)	0.093 (15/161)	1.3	0.4
**HLADQA1_0401**	0.078 (91/1170)	0.054 (13/240)	0.74	0.34	0.094 (38/404)	0 (0/79)	0	0.98	0.069 (53/766)	0.081 (13/161)	1.38	0.33
**HLADPB1_0901**	0.016 (19/1170)	0.025 (6/240)	1.56	0.36	0.02 (8/404)	0.051 (4/79)	2.71	0.12	0.014 (11/766)	0.012 (2/161)	0.84	0.82
**HLAA_3101**	0.048 (56/1170)	0.033 (8/240)	0.71	0.37	0.05 (20/404)	0.038 (3/79)	0.77	0.68	0.047 (36/766)	0.031 (5/161)	0.7	0.48
**HLAB_5601**	0.025 (29/1170)	0.017 (4/240)	0.62	0.39	0.025 (10/404)	0.025 (2/79)	1.14	0.87	0.025 (19/766)	0.012 (2/161)	0.46	0.3
**HLADQB1_0601**	0.01 (12/1170)	0.017 (4/240)	1.67	0.39	0.017 (7/404)	0 (0/79)	NA	0.99	0.007 (5/766)	0.025 (4/161)	4	0.047
**HLAC_0704**	0.032 (38/1170)	0.021 (5/240)	0.66	0.4	0.027 (11/404)	0 (0/79)	NA	0.98	0.035 (27/766)	0.031 (5/161)	1.03	0.96
**HLADRB1_1502**	0.01 (12/1170)	0.017 (4/240)	1.63	0.41	0.015 (6/404)	0 (0/79)	NA	0.98	0.008 (6/766)	0.025 (4/161)	3.43	0.067
**HLADRB3_0301**	0.076 (89/1170)	0.058 (14/240)	0.78	0.41	0.069 (28/404)	0.025 (2/79)	0.29	0.1	0.08 (61/766)	0.075 (12/161)	1.06	0.86
**HLADQB1_0502**	0.047 (55/1170)	0.042 (10/240)	0.75	0.42	0.035 (14/404)	0.051 (4/79)	1.21	0.75	0.054 (41/766)	0.037 (6/161)	0.61	0.27
**HLAA_0205**	0.021 (24/1170)	0.017 (4/240)	0.64	0.43	0.017 (7/404)	0.013 (1/79)	0.65	0.69	0.022 (17/766)	0.019 (3/161)	0.69	0.57
**HLADPB1_1401**	0.036 (42/1170)	0.05 (12/240)	1.31	0.43	0.025 (10/404)	0.038 (3/79)	1.36	0.66	0.042 (32/766)	0.056 (9/161)	1.29	0.53
**HLAC_0401**	0.238 (278/1170)	0.233 (56/240)	0.88	0.45	0.21 (85/404)	0.203 (16/79)	0.87	0.66	0.252 (193/766)	0.248 (40/161)	0.89	0.56
**HLADRB1_1302**	0.079 (92/1170)	0.062 (15/240)	0.81	0.46	0.069 (28/404)	0.038 (3/79)	0.45	0.21	0.084 (64/766)	0.075 (12/161)	1.01	0.98
**HLAA_3001**	0.021 (24/1170)	0.017 (4/240)	0.66	0.46	0.022 (9/404)	0.038 (3/79)	1.35	0.66	0.02 (15/766)	0.006 (1/161)	0.25	0.19
**HLAA_0302**	0.011 (13/1170)	0.008 (2/240)	0.57	0.46	0.007 (3/404)	0.013 (1/79)	1.75	0.64	0.013 (10/766)	0.006 (1/161)	0.33	0.31
**HLADPB1_0201**	0.209 (245/1170)	0.246 (59/240)	1.13	0.49	0.188 (76/404)	0.241 (19/79)	1.29	0.4	0.221 (169/766)	0.248 (40/161)	1.08	0.7
**HLAB_3701**	0.026 (30/1170)	0.017 (4/240)	0.7	0.5	0.032 (13/404)	0.013 (1/79)	0.42	0.4	0.022 (17/766)	0.019 (3/161)	0.88	0.84
**HLAA_6601**	0.013 (15/1170)	0.008 (2/240)	0.6	0.5	0.01 (4/404)	0 (0/79)	NA	0.98	0.014 (11/766)	0.012 (2/161)	0.82	0.8
**HLAB_4403**	0.05 (58/1170)	0.058 (14/240)	1.23	0.52	0.064 (26/404)	0.089 (7/79)	1.57	0.32	0.042 (32/766)	0.043 (7/161)	1.03	0.94
**HLAC_0701**	0.341 (399/1170)	0.312 (75/240)	0.91	0.52	0.366 (148/404)	0.354 (28/79)	0.92	0.76	0.328 (251/766)	0.292 (47/161)	0.91	0.62
**HLADPB1_1501**	0.009 (11/1170)	0.008 (2/240)	0.61	0.53	0.002 (1/404)	0 (0/79)	NA	0.99	0.013 (10/766)	0.012 (2/161)	0.6	0.54

**HLAC_1601**	0.037 (43/1170)	0.046 (11/240)	1.24	0.54	0.047 (19/404)	0.076 (6/79)	1.78	0.25	0.031 (24/766)	0.031 (5/161)	0.99	0.99
**HLADRB1_1103**	0.013 (15/1170)	0.008 (2/240)	0.65	0.57	0.01 (4/404)	0 (0/79)	NA	0.98	0.014 (11/766)	0.012 (2/161)	0.86	0.84
**HLAB_5001**	0.015 (18/1170)	0.021 (5/240)	1.34	0.58	0.017 (7/404)	0.051 (4/79)	3.2	0.075	0.014 (11/766)	0.006 (1/161)	0.43	0.43
**HLAB_4102**	0.011 (13/1170)	0.008 (2/240)	0.66	0.59	0.002 (1/404)	0 (0/79)	NA	0.99	0.016 (12/766)	0.012 (2/161)	0.72	0.68
**HLADQB1_0603**	0.074 (86/1170)	0.083 (20/240)	1.14	0.63	0.059 (24/404)	0.063 (5/79)	1.14	0.8	0.081 (62/766)	0.093 (15/161)	1.07	0.83
**HLADPB1_1101**	0.022 (26/1170)	0.025 (6/240)	1.25	0.63	0.03 (12/404)	0.051 (4/79)	1.95	0.27	0.018 (14/766)	0.012 (2/161)	0.85	0.83
**HLADPB1_1701**	0.021 (25/1170)	0.025 (6/240)	1.25	0.64	0.025 (10/404)	0.025 (2/79)	1.12	0.89	0.02 (15/766)	0.025 (4/161)	1.36	0.6
**HLAC_0202**	0.079 (93/1170)	0.088 (21/240)	1.13	0.64	0.054 (22/404)	0.101 (8/79)	2.01	0.11	0.093 (71/766)	0.081 (13/161)	0.86	0.65
**HLAC_1505**	0.009 (10/1170)	0.017 (4/240)	1.33	0.64	0.007 (3/404)	0.025 (2/79)	2.32	0.39	0.009 (7/766)	0.012 (2/161)	1.02	0.98
**HLAB_1501**	0.097 (114/1170)	0.1 (24/240)	1.12	0.64	0.089 (36/404)	0.114 (9/79)	1.43	0.38	0.102 (78/766)	0.093 (15/161)	0.95	0.87
**HLADRB1_1303**	0.015 (18/1170)	0.021 (5/240)	1.27	0.65	0.01 (4/404)	0.038 (3/79)	3.98	0.087	0.018 (14/766)	0.012 (2/161)	0.65	0.57
**HLAB_4002**	0.026 (31/1170)	0.021 (5/240)	0.8	0.66	0.01 (4/404)	0.025 (2/79)	2.38	0.34	0.035 (27/766)	0.019 (3/161)	0.51	0.29
**HLAB_4001**	0.106 (124/1170)	0.088 (21/240)	0.9	0.67	0.121 (49/404)	0.063 (5/79)	0.55	0.22	0.098 (75/766)	0.099 (16/161)	1.17	0.6
**HLAA_0301**	0.254 (297/1170)	0.258 (62/240)	1.07	0.67	0.255 (103/404)	0.203 (16/79)	0.76	0.37	0.253 (194/766)	0.286 (46/161)	1.23	0.3
**HLAA_3301**	0.016 (19/1170)	0.012 (3/240)	0.79	0.71	0.01 (4/404)	0 (0/79)	NA	0.98	0.02 (15/766)	0.019 (3/161)	1	1
**HLAA_6801**	0.056 (66/1170)	0.062 (15/240)	1.11	0.72	0.045 (18/404)	0.063 (5/79)	1.5	0.45	0.063 (48/766)	0.062 (10/161)	0.97	0.93
**HLAB_5301**	0.009 (10/1170)	0.012 (3/240)	1.27	0.72	0.002 (1/404)	0.013 (1/79)	3.34	0.41	0.012 (9/766)	0.012 (2/161)	0.91	0.9
**HLAB_4101**	0.009 (11/1170)	0.017 (4/240)	1.24	0.73	0.012 (5/404)	0.013 (1/79)	1.05	0.96	0.008 (6/766)	0.019 (3/161)	1.31	0.72
**HLADRB3_9901**	0.834 (976/1170)	0.829 (199/240)	1.07	0.73	0.844 (341/404)	0.861 (68/79)	1.37	0.39	0.829 (635/766)	0.814 (131/161)	0.99	0.95
**HLAB_4901**	0.021 (25/1170)	0.021 (5/240)	0.85	0.74	0.02 (8/404)	0.038 (3/79)	1.95	0.35	0.022 (17/766)	0.012 (2/161)	0.47	0.32
**HLAC_1701**	0.022 (26/1170)	0.033 (8/240)	1.15	0.75	0.017 (7/404)	0.025 (2/79)	1.55	0.6	0.025 (19/766)	0.037 (6/161)	1.1	0.85
**HLAB_2705**	0.079 (93/1170)	0.071 (17/240)	0.92	0.76	0.067 (27/404)	0.063 (5/79)	1.08	0.88	0.086 (66/766)	0.075 (12/161)	0.85	0.63
**HLAB_3503**	0.034 (40/1170)	0.033 (8/240)	0.9	0.79	0.042 (17/404)	0.013 (1/79)	0.23	0.16	0.03 (23/766)	0.043 (7/161)	1.68	0.25
**HLAC_1502**	0.048 (56/1170)	0.046 (11/240)	0.91	0.79	0.05 (20/404)	0.025 (2/79)	0.47	0.31	0.047 (36/766)	0.056 (9/161)	1.08	0.85
**HLAB_3801**	0.04 (47/1170)	0.05 (12/240)	0.91	0.8	0.025 (10/404)	0.051 (4/79)	1.97	0.27	0.048 (37/766)	0.05 (8/161)	0.65	0.32
**HLAA_3201**	0.058 (68/1170)	0.054 (13/240)	0.93	0.82	0.052 (21/404)	0.038 (3/79)	0.61	0.45	0.061 (47/766)	0.062 (10/161)	1.1	0.79
**HLADPB1_0402**	0.233 (273/1170)	0.233 (56/240)	0.97	0.83	0.24 (97/404)	0.203 (16/79)	0.79	0.44	0.23 (176/766)	0.248 (40/161)	1.04	0.86
**HLAC_0303**	0.09 (105/1170)	0.079 (19/240)	0.95	0.83	0.082 (33/404)	0.114 (9/79)	1.49	0.32	0.094 (72/766)	0.062 (10/161)	0.7	0.31
**HLADQB1_0202**	0.103 (120/1170)	0.096 (23/240)	0.95	0.84	0.097 (39/404)	0.152 (12/79)	1.65	0.17	0.106 (81/766)	0.068 (11/161)	0.66	0.22
**HLADRB1_0403**	0.015 (17/1170)	0.017 (4/240)	1.12	0.84	0.02 (8/404)	0 (0/79)	NA	0.99	0.012 (9/766)	0.025 (4/161)	2.16	0.23
**HLADPB1_0202**	0.009 (11/1170)	0.008 (2/240)	0.88	0.87	0.005 (2/404)	0 (0/79)	NA	0.98	0.012 (9/766)	0.012 (2/161)	0.97	0.97
**HLADRB1_0103**	0.026 (31/1170)	0.021 (5/240)	0.93	0.88	0.03 (12/404)	0.013 (1/79)	0.54	0.56	0.025 (19/766)	0.025 (4/161)	1.23	0.72
**HLADRB4_0101**	0.111 (130/1170)	0.108 (26/240)	1.03	0.89	0.111 (45/404)	0.114 (9/79)	1.05	0.89	0.111 (85/766)	0.106 (17/161)	1.03	0.91
**HLAA_0101**	0.316 (370/1170)	0.3 (72/240)	0.98	0.9	0.344 (139/404)	0.304 (24/79)	0.86	0.58	0.302 (231/766)	0.298 (48/161)	1.01	0.96
**HLAB_4501**	0.011 (13/1170)	0.012 (3/240)	1.09	0.9	0.007 (3/404)	0.013 (1/79)	1.69	0.66	0.013 (10/766)	0.012 (2/161)	0.95	0.95
**HLADQB1_0604**	0.051 (60/1170)	0.05 (12/240)	1.02	0.95	0.042 (17/404)	0.038 (3/79)	0.77	0.69	0.056 (43/766)	0.056 (9/161)	1.19	0.66
**HLADRB4_9901**	0.931 (1089/1170)	0.929 (223/240)	0.99	0.96	0.928 (375/404)	0.911 (72/79)	0.77	0.57	0.932 (714/766)	0.938 (151/161)	1.1	0.79
**HLAB_1302**	0.025 (29/1170)	0.029 (7/240)	1.02	0.96	0.022 (9/404)	0.025 (2/79)	0.87	0.87	0.026 (20/766)	0.031 (5/161)	1.03	0.95
**HLADPA1_0202**	0.05 (59/1170)	0.05 (12/240)	0.99	0.97	0.05 (20/404)	0.051 (4/79)	1.01	0.99	0.051 (39/766)	0.05 (8/161)	0.96	0.91
**HLADRB1_1102**	0.013 (15/1170)	0 (0/240)	NA	0.97	0.005 (2/404)	0 (0/79)	NA	0.98	0.017 (13/766)	0 (0/161)	NA	0.97
**HLAC_1602**	0.009 (10/1170)	0 (0/240)	NA	0.97	0.007 (3/404)	0 (0/79)	NA	0.99	0.009 (7/766)	0 (0/161)	NA	0.98
**HLADRB1_0408**	0.009 (10/1170)	0 (0/240)	NA	0.98	0.01 (4/404)	0 (0/79)	NA	0.98	0.008 (6/766)	0 (0/161)	NA	0.98
**HLAB_3906**	0.02 (23/1170)	0.017 (4/240)	0.99	0.98	0.02 (8/404)	0.025 (2/79)	1.58	0.58	0.02 (15/766)	0.012 (2/161)	0.63	0.55

Only alleles seen in >=10 ATA- SSc patients in the meta-analysis cohort are shown. Rare alleles for which odds ratios could not be accurately computed are assigned OR = NA. CO = control, OR = odds ratio, NA = not applicable.

Supplementary Table 9: Classical HLA allele frequency differences between ANoA+ and ANoA- SSc in all three study cohorts.

	ANoA+ vs -											
	Meta-analysis Cohort				Cohort 1				Cohort 2			
HLA Allele	ANoA- Prop.(Count)	ANoA+ Prop.(Count)	OR	P	ANoA- Prop.(Count)	ANoA+ Prop.(Count)	OR	P	ANoA- Prop.(Count)	ANoA+ Prop.(Count)	OR	P
HLADQA1_0101	0.34 (359/1055)	0.201 (71/353)	0.48	**9.20E-07**	0.335 (119/355)	0.244 (31/127)	0.62	0.042	0.343 (240/700)	0.177 (40/226)	0.41	**4.60E-06**
HLADQA1_0501	0.474 (500/1055)	0.626 (221/353)	1.84	**1.60E-06**	0.473 (168/355)	0.622 (79/127)	1.84	0.0048	0.474 (332/700)	0.628 (142/226)	1.83	0.00015
HLADQB1_0201	0.227 (240/1055)	0.337 (119/353)	1.88	**4.70E-06**	0.262 (93/355)	0.339 (43/127)	1.47	0.094	0.21 (147/700)	0.336 (76/226)	2.21	6.40E-06
HLAC_0701	0.304 (321/1055)	0.433 (153/353)	1.78	5.80E-06	0.332 (118/355)	0.457 (58/127)	1.69	0.015	0.29 (203/700)	0.42 (95/226)	1.86	0.00012
HLADRB1_0301	0.225 (237/1055)	0.331 (117/353)	1.86	6.80E-06	0.254 (90/355)	0.331 (42/127)	1.49	0.082	0.21 (147/700)	0.332 (75/226)	2.16	1.20E-05
HLADRB1_1104	0.095 (100/1055)	0.195 (69/353)	2.27	7.20E-06	0.079 (28/355)	0.165 (21/127)	2.51	0.0067	0.103 (72/700)	0.212 (48/226)	2.12	0.00066
HLADQB1_0501	0.295 (311/1055)	0.181 (64/353)	0.53	3.40E-05	0.29 (103/355)	0.228 (29/127)	0.7	0.15	0.297 (208/700)	0.155 (35/226)	0.43	3.90E-05
HLAB_1801	0.098 (103/1055)	0.187 (66/353)	2.05	3.80E-05	0.101 (36/355)	0.236 (30/127)	2.8	0.00028	0.096 (67/700)	0.159 (36/226)	1.7	0.02
HLAB_0801	0.229 (242/1055)	0.317 (112/353)	1.68	0.00018	0.242 (86/355)	0.307 (39/127)	1.41	0.15	0.223 (156/700)	0.323 (73/226)	1.85	0.00045
HLAA_0101	0.289 (305/1055)	0.385 (136/353)	1.61	0.00028	0.315 (112/355)	0.394 (50/127)	1.4	0.13	0.276 (193/700)	0.381 (86/226)	1.71	0.0011
HLADRB3_0101	0.24 (253/1055)	0.32 (113/353)	1.63	0.00043	0.254 (90/355)	0.315 (40/127)	1.39	0.16	0.233 (163/700)	0.323 (73/226)	1.81	0.00075
HLADPB1_0301	0.247 (261/1055)	0.164 (58/353)	0.6	0.0014	0.234 (83/355)	0.165 (21/127)	0.66	0.13	0.254 (178/700)	0.164 (37/226)	0.57	0.0061
HLADRB3_9901	0.853 (900/1055)	0.773 (273/353)	0.61	0.0014	0.854 (303/355)	0.827 (105/127)	0.81	0.46	0.853 (597/700)	0.743 (168/226)	0.52	0.00058
HLADRB1_0801	0.078 (82/1055)	0.025 (9/353)	0.32	0.0016	0.087 (31/355)	0.024 (3/127)	0.27	0.033	0.073 (51/700)	0.027 (6/226)	0.37	0.025
HLADPB1_1301	0.063 (66/1055)	0.116 (41/353)	1.93	0.0019	0.054 (19/355)	0.094 (12/127)	1.87	0.12	0.067 (47/700)	0.128 (29/226)	2.05	0.0046
HLADRB1_0101	0.227 (239/1055)	0.147 (52/353)	0.6	0.0024	0.223 (79/355)	0.189 (24/127)	0.8	0.4	0.229 (160/700)	0.124 (28/226)	0.5	0.0017
HLADQB1_0402	0.086 (91/1055)	0.037 (13/353)	0.42	0.0042	0.099 (35/355)	0.024 (3/127)	0.23	0.016	0.08 (56/700)	0.044 (10/226)	0.56	0.1
HLAA_0201	0.497 (524/1055)	0.402 (142/353)	0.71	0.0056	0.476 (169/355)	0.457 (58/127)	0.92	0.71	0.507 (355/700)	0.372 (84/226)	0.62	0.0025
HLADQA1_0401	0.084 (89/1055)	0.037 (13/353)	0.43	0.0059	0.096 (34/355)	0.024 (3/127)	0.23	0.019	0.079 (55/700)	0.044 (10/226)	0.57	0.12
HLAB_4402	0.191 (201/1055)	0.122 (43/353)	0.61	0.0072	0.203 (72/355)	0.126 (16/127)	0.56	0.061	0.184 (129/700)	0.119 (27/226)	0.63	0.045
HLADQA1_0102	0.314 (331/1055)	0.388 (137/353)	1.41	0.0081	0.304 (108/355)	0.402 (51/127)	1.54	0.047	0.319 (223/700)	0.381 (86/226)	1.36	0.06
HLAB_3501	0.15 (158/1055)	0.096 (34/353)	0.59	0.0083	0.13 (46/355)	0.087 (11/127)	0.62	0.18	0.16 (112/700)	0.102 (23/226)	0.58	0.026
HLADPA1_0201	0.284 (300/1055)	0.354 (125/353)	1.4	0.011	0.299 (106/355)	0.307 (39/127)	1.06	0.82	0.277 (194/700)	0.381 (86/226)	1.68	0.0016
HLADRB1_0102	0.033 (35/1055)	0.008 (3/353)	0.22	0.012	0.031 (11/355)	0.008 (1/127)	0.22	0.16	0.034 (24/700)	0.009 (2/226)	0.22	0.042
HLAC_0401	0.251 (265/1055)	0.195 (69/353)	0.68	0.013	0.242 (86/355)	0.118 (15/127)	0.4	0.0027	0.256 (179/700)	0.239 (54/226)	0.86	0.43
HLADPB1_0101	0.097 (102/1055)	0.136 (48/353)	1.56	0.019	0.093 (33/355)	0.134 (17/127)	1.58	0.16	0.099 (69/700)	0.137 (31/226)	1.65	0.036
HLADQB1_0302	0.22 (232/1055)	0.161 (57/353)	0.68	0.019	0.208 (74/355)	0.157 (20/127)	0.73	0.27	0.226 (158/700)	0.164 (37/226)	0.67	0.051
HLADRB1_1301	0.081 (85/1055)	0.042 (15/353)	0.52	0.022	0.062 (22/355)	0.055 (7/127)	0.93	0.87	0.09 (63/700)	0.035 (8/226)	0.37	0.0092
HLADRB1_1401	0.057 (60/1055)	0.028 (10/353)	0.45	0.024	0.054 (19/355)	0.016 (2/127)	0.26	0.078	0.059 (41/700)	0.035 (8/226)	0.56	0.15
HLADQB1_0604	0.043 (45/1055)	0.074 (26/353)	1.78	0.024	0.042 (15/355)	0.031 (4/127)	0.72	0.58	0.043 (30/700)	0.097 (22/226)	2.63	0.0011
HLAC_1202	0.01 (11/1055)	0.028 (10/353)	2.68	0.027	0.014 (5/355)	0.024 (3/127)	1.65	0.5	0.009 (6/700)	0.031 (7/226)	3.71	0.022
HLADQB1_0503	0.058 (61/1055)	0.031 (11/353)	0.49	0.035	0.059 (21/355)	0.016 (2/127)	0.23	0.05	0.057 (40/700)	0.04 (9/226)	0.67	0.29
HLAB_5201	0.011 (12/1055)	0.028 (10/353)	2.47	0.038	0.014 (5/355)	0.024 (3/127)	1.65	0.5	0.01 (7/700)	0.031 (7/226)	3.19	0.034
HLADRB3_0301	0.064 (68/1055)	0.096 (34/353)	1.56	0.045	0.062 (22/355)	0.055 (7/127)	0.91	0.83	0.066 (46/700)	0.119 (27/226)	2.04	0.0062
HLADQA1_0301	0.343 (362/1055)	0.28 (99/353)	0.76	0.047	0.346 (123/355)	0.299 (38/127)	0.82	0.39	0.341 (239/700)	0.27 (61/226)	0.73	0.072
HLADRB5_0101	0.218 (230/1055)	0.263 (93/353)	1.33	0.05	0.22 (78/355)	0.315 (40/127)	1.65	0.034	0.217 (152/700)	0.235 (53/226)	1.14	0.49
HLADRB1_1302	0.067 (71/1055)	0.099 (35/353)	1.53	0.052	0.065 (23/355)	0.055 (7/127)	0.87	0.75	0.069 (48/700)	0.124 (28/226)	2.03	0.0058
HLADRB1_0404	0.088 (93/1055)	0.054 (19/353)	0.61	0.058	0.073 (26/355)	0.047 (6/127)	0.64	0.34	0.096 (67/700)	0.058 (13/226)	0.61	0.11
HLAC_0501	0.173 (183/1055)	0.127 (45/353)	0.72	0.068	0.177 (63/355)	0.15 (19/127)	0.84	0.55	0.171 (120/700)	0.115 (26/226)	0.66	0.076
HLADRB1_1501	0.216 (228/1055)	0.258 (91/353)	1.3	0.07	0.22 (78/355)	0.315 (40/127)	1.65	0.034	0.214 (150/700)	0.226 (51/226)	1.1	0.62
HLAB_1402	0.066 (70/1055)	0.042 (15/353)	0.59	0.074	0.054 (19/355)	0.031 (4/127)	0.56	0.31	0.073 (51/700)	0.049 (11/226)	0.6	0.14
HLAC_0202	0.088 (93/1055)	0.059 (21/353)	0.64	0.079	0.065 (23/355)	0.055 (7/127)	0.89	0.8	0.1 (70/700)	0.062 (14/226)	0.57	0.067
HLAC_0802	0.08 (84/1055)	0.054 (19/353)	0.63	0.081	0.068 (24/355)	0.047 (6/127)	0.65	0.37	0.086 (60/700)	0.058 (13/226)	0.61	0.12
HLADQB1_0603	0.082 (87/1055)	0.054 (19/353)	0.63	0.082	0.062 (22/355)	0.055 (7/127)	0.93	0.88	0.093 (65/700)	0.053 (12/226)	0.52	0.048
HLAA_2402	0.174 (184/1055)	0.218 (77/353)	1.29	0.093	0.192 (68/355)	0.213 (27/127)	1.13	0.65	0.166 (116/700)	0.221 (50/226)	1.42	0.071
HLADPA1_0103	0.969 (1022/1055)	0.949 (335/353)	0.61	0.098	0.961 (341/355)	0.976 (124/127)	1.55	0.5	0.973 (681/700)	0.934 (211/226)	0.4	0.011
HLADQB1_0602	0.21 (222/1055)	0.244 (86/353)	1.26	0.12	0.208 (74/355)	0.307 (39/127)	1.72	0.024	0.211 (148/700)	0.208 (47/226)	1.02	0.91
HLAB_3503	0.039 (41/1055)	0.023 (8/353)	0.55	0.12	0.048 (17/355)	0.016 (2/127)	0.29	0.11	0.034 (24/700)	0.027 (6/226)	0.82	0.67

HLAA_3002	0.013 (14/1055)	0.025 (9/353)	1.88	0.15	0.008 (3/355)	0.016 (2/127)	2	0.46	0.016 (11/700)	0.031 (7/226)	1.92	0.19
HLAA_2902	0.041 (43/1055)	0.059 (21/353)	1.49	0.15	0.042 (15/355)	0.063 (8/127)	1.68	0.26	0.04 (28/700)	0.058 (13/226)	1.37	0.37
HLADRB5_9901	0.978 (1032/1055)	0.963 (340/353)	0.6	0.15	0.977 (347/355)	0.976 (124/127)	1.17	0.82	0.979 (685/700)	0.956 (216/226)	0.5	0.099
HLADQB1_0303	0.064 (68/1055)	0.085 (30/353)	1.38	0.16	0.073 (26/355)	0.079 (10/127)	1.1	0.81	0.06 (42/700)	0.088 (20/226)	1.58	0.11
HLADRB1_0103	0.029 (31/1055)	0.014 (5/353)	0.5	0.16	0.034 (12/355)	0.008 (1/127)	0.19	0.12	0.027 (19/700)	0.018 (4/226)	0.7	0.53
HLAB_4405	0.01 (11/1055)	0.003 (1/353)	0.25	0.18	0.014 (5/355)	0 (0/127)	NA	0.98	0.009 (6/700)	0.004 (1/226)	0.52	0.55
HLADPB1_1401	0.034 (36/1055)	0.051 (18/353)	1.49	0.18	0.025 (9/355)	0.031 (4/127)	1.19	0.78	0.039 (27/700)	0.062 (14/226)	1.65	0.15
HLADRB1_0407	0.031 (33/1055)	0.017 (6/353)	0.55	0.18	0.031 (11/355)	0.024 (3/127)	0.71	0.61	0.031 (22/700)	0.013 (3/226)	0.42	0.17
HLADPB1_0401	0.676 (713/1055)	0.635 (224/353)	0.84	0.19	0.696 (247/355)	0.638 (81/127)	0.74	0.18	0.666 (466/700)	0.633 (143/226)	0.87	0.4
HLADQA1_0103	0.087 (92/1055)	0.065 (23/353)	0.73	0.19	0.076 (27/355)	0.055 (7/127)	0.72	0.46	0.093 (65/700)	0.071 (16/226)	0.72	0.27
HLAA_3201	0.053 (56/1055)	0.074 (26/353)	1.38	0.19	0.051 (18/355)	0.055 (7/127)	1.06	0.89	0.054 (38/700)	0.084 (19/226)	1.59	0.12
HLAA_0301	0.265 (280/1055)	0.227 (80/353)	0.83	0.2	0.265 (94/355)	0.205 (26/127)	0.7	0.17	0.266 (186/700)	0.239 (54/226)	0.88	0.5
HLAA_6801	0.063 (66/1055)	0.042 (15/353)	0.69	0.2	0.054 (19/355)	0.031 (4/127)	0.63	0.42	0.067 (47/700)	0.049 (11/226)	0.72	0.34
HLAB_3508	0.014 (15/1055)	0.025 (9/353)	1.72	0.21	0.02 (7/355)	0.008 (1/127)	0.44	0.45	0.011 (8/700)	0.035 (8/226)	3.2	0.025
HLADPB1_0901	0.02 (21/1055)	0.011 (4/353)	0.52	0.23	0.028 (10/355)	0.016 (2/127)	0.49	0.37	0.016 (11/700)	0.009 (2/226)	0.48	0.35
HLAB_5701	0.048 (51/1055)	0.062 (22/353)	1.37	0.23	0.045 (16/355)	0.055 (7/127)	1.19	0.71	0.05 (35/700)	0.066 (15/226)	1.43	0.27
HLADRB1_0401	0.166 (175/1055)	0.13 (46/353)	0.81	0.24	0.183 (65/355)	0.157 (20/127)	0.87	0.62	0.157 (110/700)	0.115 (26/226)	0.77	0.28
HLAA_0205	0.022 (23/1055)	0.014 (5/353)	0.55	0.24	0.017 (6/355)	0.016 (2/127)	0.91	0.91	0.024 (17/700)	0.013 (3/226)	0.44	0.2
HLADPB1_0402	0.241 (254/1055)	0.212 (75/353)	0.84	0.25	0.237 (84/355)	0.228 (29/127)	0.95	0.85	0.243 (170/700)	0.204 (46/226)	0.77	0.17
HLAB_3906	0.022 (23/1055)	0.011 (4/353)	0.54	0.25	0.02 (7/355)	0.024 (3/127)	1.12	0.87	0.023 (16/700)	0.004 (1/226)	0.19	0.11
HLAB_2705	0.083 (88/1055)	0.062 (22/353)	0.75	0.26	0.073 (26/355)	0.047 (6/127)	0.63	0.32	0.089 (62/700)	0.071 (16/226)	0.8	0.46
HLADRB1_1102	0.012 (13/1055)	0.006 (2/353)	0.43	0.27	0.006 (2/355)	0 (0/127)	NA	0.98	0.016 (11/700)	0.009 (2/226)	0.57	0.47
HLAB_4102	0.012 (13/1055)	0.006 (2/353)	0.44	0.28	0.003 (1/355)	0 (0/127)	NA	0.98	0.017 (12/700)	0.009 (2/226)	0.49	0.35
HLADRB4_0103	0.376 (397/1055)	0.34 (120/353)	0.87	0.29	0.4 (142/355)	0.339 (43/127)	0.78	0.27	0.364 (255/700)	0.341 (77/226)	0.93	0.65
HLAB_4901	0.019 (20/1055)	0.028 (10/353)	1.51	0.3	0.014 (5/355)	0.047 (6/127)	3.99	0.029	0.021 (15/700)	0.018 (4/226)	0.79	0.68
HLADQA1_0201	0.149 (157/1055)	0.167 (59/353)	1.18	0.33	0.163 (58/355)	0.157 (20/127)	0.98	0.94	0.141 (99/700)	0.173 (39/226)	1.29	0.22
HLADRB1_0901	0.015 (16/1055)	0.023 (8/353)	1.53	0.34	0.017 (6/355)	0.031 (4/127)	2.07	0.27	0.014 (10/700)	0.018 (4/226)	1.32	0.64
HLADRB1_1001	0.021 (22/1055)	0.014 (5/353)	0.62	0.34	0.014 (5/355)	0.031 (4/127)	2.38	0.21	0.024 (17/700)	0.004 (1/226)	0.15	0.07
HLADRB1_1303	0.018 (19/1055)	0.011 (4/353)	0.59	0.35	0.017 (6/355)	0.008 (1/127)	0.46	0.47	0.019 (13/700)	0.013 (3/226)	0.63	0.48
HLAB_5801	0.017 (18/1055)	0.011 (4/353)	0.59	0.35	0.02 (7/355)	0.008 (1/127)	0.41	0.41	0.016 (11/700)	0.013 (3/226)	0.72	0.62
HLAB_5601	0.026 (27/1055)	0.017 (6/353)	0.66	0.36	0.025 (9/355)	0.024 (3/127)	1.12	0.86	0.026 (18/700)	0.013 (3/226)	0.49	0.26
HLAA_2301	0.034 (36/1055)	0.023 (8/353)	0.7	0.37	0.039 (14/355)	0.039 (5/127)	0.99	0.99	0.031 (22/700)	0.013 (3/226)	0.43	0.18
HLADRB1_0402	0.027 (29/1055)	0.045 (16/353)	1.35	0.37	0.023 (8/355)	0.031 (4/127)	1.41	0.6	0.03 (21/700)	0.053 (12/226)	1.32	0.49
HLADRB1_1101	0.138 (146/1055)	0.125 (44/353)	0.85	0.39	0.124 (44/355)	0.165 (21/127)	1.47	0.2	0.146 (102/700)	0.102 (23/226)	0.62	0.056
HLADRB3_0202	0.372 (392/1055)	0.414 (146/353)	1.12	0.39	0.338 (120/355)	0.378 (48/127)	1.19	0.45	0.389 (272/700)	0.434 (98/226)	1.08	0.63
HLADRB1_0701	0.149 (157/1055)	0.164 (58/353)	1.15	0.4	0.163 (58/355)	0.15 (19/127)	0.92	0.78	0.141 (99/700)	0.173 (39/226)	1.29	0.22
HLADPB1_0601	0.033 (35/1055)	0.04 (14/353)	1.31	0.41	0.031 (11/355)	0.039 (5/127)	1.31	0.63	0.034 (24/700)	0.04 (9/226)	1.22	0.63
HLAB_5101	0.095 (100/1055)	0.082 (29/353)	0.83	0.42	0.073 (26/355)	0.11 (14/127)	1.63	0.17	0.106 (74/700)	0.066 (15/226)	0.58	0.069
HLADQB1_0502	0.043 (45/1055)	0.057 (20/353)	1.25	0.43	0.034 (12/355)	0.047 (6/127)	1.38	0.54	0.047 (33/700)	0.062 (14/226)	1.21	0.57
HLADPB1_1001	0.042 (44/1055)	0.031 (11/353)	0.77	0.44	0.054 (19/355)	0.024 (3/127)	0.47	0.23	0.036 (25/700)	0.035 (8/226)	1.04	0.93
HLAA_6601	0.013 (14/1055)	0.008 (3/353)	0.61	0.44	0.008 (3/355)	0.008 (1/127)	0.97	0.98	0.016 (11/700)	0.009 (2/226)	0.55	0.44
HLADPB1_0201	0.209 (220/1055)	0.235 (83/353)	1.12	0.45	0.186 (66/355)	0.22 (28/127)	1.27	0.36	0.22 (154/700)	0.243 (55/226)	1.09	0.62
HLAA_3301	0.017 (18/1055)	0.011 (4/353)	0.66	0.45	0.011 (4/355)	0 (0/127)	NA	0.98	0.02 (14/700)	0.018 (4/226)	0.89	0.84
HLADRB1_0405	0.009 (10/1055)	0.014 (5/353)	1.49	0.48	0.014 (5/355)	0.024 (3/127)	1.7	0.48	0.007 (5/700)	0.009 (2/226)	1.56	0.6
HLADQB1_0301	0.399 (421/1055)	0.431 (152/353)	1.09	0.48	0.38 (135/355)	0.449 (57/127)	1.32	0.2	0.409 (286/700)	0.42 (95/226)	0.97	0.83
HLAC_0602	0.126 (133/1055)	0.139 (49/353)	1.14	0.48	0.124 (44/355)	0.142 (18/127)	1.16	0.62	0.127 (89/700)	0.137 (31/226)	1.11	0.64
HLAC_1203	0.117 (123/1055)	0.142 (50/353)	1.14	0.48	0.099 (35/355)	0.165 (21/127)	1.87	0.04	0.126 (88/700)	0.128 (29/226)	0.85	0.51
HLAA_0302	0.011 (12/1055)	0.008 (3/353)	0.63	0.49	0.006 (2/355)	0.016 (2/127)	3.1	0.27	0.014 (10/700)	0.004 (1/226)	0.22	0.16
HLAC_1402	0.029 (31/1055)	0.023 (8/353)	0.77	0.51	0.028 (10/355)	0.039 (5/127)	1.39	0.56	0.03 (21/700)	0.013 (3/226)	0.44	0.19
HLADPB1_0501	0.044 (46/1055)	0.034 (12/353)	0.81	0.53	0.054 (19/355)	0.031 (4/127)	0.62	0.4	0.039 (27/700)	0.035 (8/226)	0.99	0.99
HLAB_3801	0.043 (45/1055)	0.04 (14/353)	0.82	0.54	0.028 (10/355)	0.031 (4/127)	1.17	0.8	0.05 (35/700)	0.044 (10/226)	0.69	0.35
HLAB_4501	0.01 (11/1055)	0.014 (5/353)	1.38	0.56	0.006 (2/355)	0.016 (2/127)	3.03	0.27	0.013 (9/700)	0.013 (3/226)	1.02	0.97
HLADPA1_0202	0.049 (52/1055)	0.057 (20/353)	1.17	0.56	0.045 (16/355)	0.071 (9/127)	1.68	0.24	0.051 (36/700)	0.049 (11/226)	0.92	0.82
HLADPB1_1701	0.021 (22/1055)	0.025 (9/353)	1.26	0.56	0.028 (10/355)	0.016 (2/127)	0.53	0.41	0.017 (12/700)	0.031 (7/226)	1.96	0.17
HLAA_1101	0.119 (126/1055)	0.108 (38/353)	0.89	0.57	0.107 (38/355)	0.102 (13/127)	0.95	0.89	0.126 (88/700)	0.111 (25/226)	0.87	0.58

HLADRB5_0202	0.036 (38/1055)	0.045 (16/353)	1.19	0.58	0.025 (9/355)	0.031 (4/127)	1.17	0.8	0.041 (29/700)	0.053 (12/226)	1.17	0.65
HLAB_3701	0.026 (27/1055)	0.02 (7/353)	0.8	0.61	0.028 (10/355)	0.031 (4/127)	1.13	0.84	0.024 (17/700)	0.013 (3/226)	0.56	0.36
HLAB_1401	0.015 (16/1055)	0.011 (4/353)	0.77	0.64	0.014 (5/355)	0.016 (2/127)	0.96	0.97	0.016 (11/700)	0.009 (2/226)	0.57	0.48
HLAC_0704	0.032 (34/1055)	0.025 (9/353)	0.84	0.65	0.025 (9/355)	0.016 (2/127)	0.68	0.63	0.036 (25/700)	0.031 (7/226)	1.01	0.99
HLADPB1_1501	0.009 (10/1055)	0.008 (3/353)	0.74	0.65	0.003 (1/355)	0 (0/127)	NA	0.98	0.013 (9/700)	0.013 (3/226)	0.77	0.71
HLADRB4_0101	0.114 (120/1055)	0.102 (36/353)	0.92	0.67	0.104 (37/355)	0.134 (17/127)	1.34	0.36	0.119 (83/700)	0.084 (19/226)	0.71	0.21
HLADRB1_1103	0.011 (12/1055)	0.014 (5/353)	1.25	0.68	0.008 (3/355)	0.008 (1/127)	0.86	0.9	0.013 (9/700)	0.018 (4/226)	1.37	0.61
HLAC_1601	0.039 (41/1055)	0.034 (12/353)	0.87	0.68	0.054 (19/355)	0.039 (5/127)	0.77	0.61	0.031 (22/700)	0.031 (7/226)	0.96	0.92
HLAB_3901	0.027 (29/1055)	0.025 (9/353)	0.86	0.69	0.023 (8/355)	0.031 (4/127)	1.56	0.49	0.03 (21/700)	0.022 (5/226)	0.69	0.46
HLAB_4403	0.052 (55/1055)	0.045 (16/353)	0.89	0.7	0.073 (26/355)	0.047 (6/127)	0.64	0.35	0.041 (29/700)	0.044 (10/226)	1.07	0.86
HLAB_4101	0.009 (10/1055)	0.014 (5/353)	1.22	0.72	0.011 (4/355)	0.016 (2/127)	1.47	0.66	0.009 (6/700)	0.013 (3/226)	1.11	0.89
HLAA_3101	0.046 (49/1055)	0.042 (15/353)	0.9	0.73	0.048 (17/355)	0.047 (6/127)	1	1	0.046 (32/700)	0.04 (9/226)	0.9	0.79
HLAB_5501	0.031 (33/1055)	0.028 (10/353)	0.88	0.74	0.042 (15/355)	0 (0/127)	NA	0.98	0.026 (18/700)	0.044 (10/226)	1.82	0.14
HLAA_2501	0.042 (44/1055)	0.045 (16/353)	1.1	0.76	0.056 (20/355)	0.063 (8/127)	1.12	0.8	0.034 (24/700)	0.035 (8/226)	1.06	0.89
HLAB_1501	0.1 (106/1055)	0.091 (32/353)	0.94	0.76	0.087 (31/355)	0.11 (14/127)	1.28	0.47	0.107 (75/700)	0.08 (18/226)	0.77	0.36
HLADPB1_1101	0.024 (25/1055)	0.02 (7/353)	0.89	0.78	0.037 (13/355)	0.024 (3/127)	0.69	0.57	0.017 (12/700)	0.018 (4/226)	1.13	0.83
HLAC_0702	0.262 (276/1055)	0.246 (87/353)	0.96	0.79	0.299 (106/355)	0.26 (33/127)	0.8	0.36	0.243 (170/700)	0.239 (54/226)	1.04	0.83
HLAA_6802	0.014 (15/1055)	0.017 (6/353)	1.14	0.8	0.011 (4/355)	0.008 (1/127)	0.68	0.73	0.016 (11/700)	0.022 (5/226)	1.31	0.63
HLADRB1_1201	0.024 (25/1055)	0.023 (8/353)	0.9	0.8	0.028 (10/355)	0.008 (1/127)	0.26	0.21	0.021 (15/700)	0.031 (7/226)	1.42	0.46
HLAB_4002	0.025 (26/1055)	0.028 (10/353)	1.09	0.81	0.006 (2/355)	0.031 (4/127)	6.62	0.034	0.034 (24/700)	0.027 (6/226)	0.72	0.48
HLAA_3001	0.02 (21/1055)	0.02 (7/353)	0.9	0.82	0.028 (10/355)	0.016 (2/127)	0.5	0.38	0.016 (11/700)	0.022 (5/226)	1.29	0.64
HLAC_1502	0.047 (50/1055)	0.045 (16/353)	0.94	0.83	0.039 (14/355)	0.055 (7/127)	1.39	0.5	0.051 (36/700)	0.04 (9/226)	0.73	0.42
HLAB_0702	0.228 (241/1055)	0.227 (80/353)	1.03	0.83	0.265 (94/355)	0.252 (32/127)	0.9	0.68	0.21 (147/700)	0.212 (48/226)	1.09	0.66
HLAC_1701	0.023 (24/1055)	0.028 (10/353)	1.08	0.84	0.017 (6/355)	0.024 (3/127)	1.53	0.57	0.026 (18/700)	0.031 (7/226)	1.02	0.97
HLAB_1302	0.026 (27/1055)	0.025 (9/353)	0.93	0.85	0.025 (9/355)	0.016 (2/127)	0.62	0.56	0.026 (18/700)	0.031 (7/226)	1.14	0.78
HLAA_2601	0.075 (79/1055)	0.085 (30/353)	1.04	0.85	0.059 (21/355)	0.071 (9/127)	1.21	0.65	0.083 (58/700)	0.093 (21/226)	0.96	0.88
HLADRB1_1601	0.035 (37/1055)	0.04 (14/353)	1.06	0.86	0.025 (9/355)	0.031 (4/127)	1.15	0.82	0.04 (28/700)	0.044 (10/226)	0.99	0.99
HLAB_4001	0.103 (109/1055)	0.102 (36/353)	1.04	0.86	0.118 (42/355)	0.094 (12/127)	0.81	0.55	0.096 (67/700)	0.106 (24/226)	1.19	0.49
HLADQB1_0202	0.101 (107/1055)	0.102 (36/353)	1.03	0.88	0.104 (37/355)	0.11 (14/127)	1.11	0.77	0.1 (70/700)	0.097 (22/226)	NA	0.99
HLAB_3502	0.035 (37/1055)	0.042 (15/353)	1.05	0.88	0.028 (10/355)	0.016 (2/127)	0.52	0.42	0.039 (27/700)	0.058 (13/226)	1.22	0.59
HLADRB1_0403	0.015 (16/1055)	0.014 (5/353)	0.93	0.88	0.017 (6/355)	0.016 (2/127)	1.03	0.97	0.014 (10/700)	0.013 (3/226)	0.89	0.87
HLAC_0303	0.089 (94/1055)	0.085 (30/353)	0.98	0.94	0.096 (34/355)	0.063 (8/127)	0.59	0.2	0.086 (60/700)	0.097 (22/226)	1.24	0.42
HLAB_5001	0.016 (17/1055)	0.017 (6/353)	1.03	0.95	0.023 (8/355)	0.024 (3/127)	1.14	0.85	0.013 (9/700)	0.013 (3/226)	0.96	0.96
HLADRB4_9901	0.93 (981/1055)	0.932 (329/353)	1.01	0.95	0.927 (329/355)	0.921 (117/127)	0.88	0.74	0.931 (652/700)	0.938 (212/226)	1.1	0.76
HLAC_0102	0.073 (77/1055)	0.074 (26/353)	1.01	0.97	0.076 (27/355)	0.063 (8/127)	0.84	0.69	0.071 (50/700)	0.08 (18/226)	1.12	0.7
HLADQB1_0609	0.023 (24/1055)	0.023 (8/353)	1.01	0.98	0.023 (8/355)	0.024 (3/127)	1.17	0.82	0.023 (16/700)	0.022 (5/226)	0.96	0.94
HLAC_0304	0.142 (150/1055)	0.136 (48/353)	1	0.99	0.138 (49/355)	0.134 (17/127)	1.01	0.97	0.144 (101/700)	0.137 (31/226)	1.01	0.96

Only alleles seen in >=10 ANoA- SSc patients in the meta-analysis cohort are shown. Rare alleles for which odds ratios could not be accurately computed are assigned OR = NA. CO = control, OR = odds ratio, NA = not applicable.

Supplementary Table 10: Statistical interactions between biologically interacting KIRs and HLA subgroups reaching statistical significance ($p<0.05$) in the meta-analysis cohort. Table shows KIR associations (+ dominant, ++ recessive) in individuals with a given HLA subgroup genotype (+ positive, - negative, +|+ homozygote, +|- heterozygote or negative).

		Meta-analysis cohort							Cohort 1							Cohort 2						
**KIR**	**Subtype**	**SSc Prop.**	**(Count)**	**CO Prop.**	**(Count)**	**OR**	**P**	**Int.p**	**SSc Prop.**	**(Count)**	**CO Prop.**	**(Count)**	**OR**	**p**	**Int.p**	**SSc Prop.**	**(Count)**	**CO Prop.**	**(Count)**	**OR**	**p**	**Int.p**
3DS1 ++	***Bw4 I80+***	0.059	(41/698)	0.049	(297/6016)	1.12	0.52	**0.01**	0.046	(11/238)	0.052	(157/2997)	0.83	0.57	**0.03**	0.065	(30/460)	0.046	(140/3019)	1.28	0.24	0.10
	Bw4 I80-	0.023	(18/767)	0.044	(321/7257)	0.50	**0.005**		0.004	(1/265)	0.044	(161/3635)	0.08	**0.01**		0.034	(17/502)	0.044	(160/3622)	0.72	0.22	
3DL1	***Bw4 I80+***	0.937	(654/698)	0.950	(5714/6016)	0.84	0.30	**0.01**	0.945	(225/238)	0.947	(2837/2997)	1.03	0.93	**0.05**	0.933	(429/460)	0.953	(2877/3019)	0.76	0.19	0.08
	Bw4 I80-	0.971	(745/767)	0.954	(6925/7257)	1.69	**0.02**		0.985	(261/265)	0.954	(3468/3635)	3.18	**0.02**		0.964	(484/502)	0.954	(3457/3622)	1.37	0.22	
3DL1	***Bw4+***	0.945	(947/1002)	0.952	(9121/9583)	0.92	0.58	**0.02**	0.954	(332/348)	0.951	(4542/4778)	1.10	0.71	0.06	0.94	(615/654)	0.953	(4579/4805)	0.85	0.39	0.15
	Bw4-	0.976	(452/463)	0.953	(3518/3690)	2.11	**0.02**		0.994	(154/155)	0.951	(1763/1854)	8.03	**0.04**		0.968	(298/308)	0.956	(1755/1836)	1.48	0.26	
3DS1 ++	***Bw4+***	0.049	(49/1002)	0.047	(450/9583)	0.98	0.92	**0.03**	0.032	(11/348)	0.048	(228/4778)	0.64	0.15	0.12	0.058	(38/654)	0.046	(222/4805)	1.16	0.43	0.14
	Bw4-	0.022	(10/463)	0.046	(168/3690)	0.44	**0.01**		0.006	(1/155)	0.049	(90/1854)	0.13	**0.04**		0.029	(9/308)	0.042	(78/1836)	0.65	0.24	
2DL3	***C1+***	0.896	(1139/1271)	0.912	(10537/11557)	0.89	0.23	**0.03**	0.889	(399/449)	0.913	(5270/5775)	0.81	0.18	0.60	0.900	(740/822)	0.911	(5267/5782)	0.93	0.58	**0.02**
	C1-	0.948	(184/194)	0.909	(1560/1716)	1.98	**0.04**		0.926	(50/54)	0.919	(788/857)	1.17	0.77		0.957	(134/140)	0.899	(772/859)	2.85	**0.02**	
2DL2 ++	***C1+***	0.104	(132/1271)	0.088	(1020/11557)	1.13	0.23	**0.03**	0.111	(50/449)	0.088	(506/5775)	1.24	0.18	0.60	0.100	(82/822)	0.089	(514/5782)	1.07	0.58	**0.02**
	C1-	0.052	(10/194)	0.091	(156/1716)	0.50	**0.04**		0.074	(4/54)	0.081	(69/857)	0.85	0.77		0.043	(6/140)	0.101	(87/859)	0.35	**0.02**	
2DS2 ++	***C1+***	0.104	(132/1271)	0.088	(1021/11557)	1.13	0.23	**0.03**	0.111	(50/449)	0.088	(506/5775)	1.24	0.18	0.60	0.100	(82/822)	0.089	(515/5782)	1.07	0.58	**0.02**
	C1-	0.052	(10/194)	0.091	(156/1716)	0.50	**0.04**		0.074	(4/54)	0.081	(69/857)	0.85	0.77		0.043	(6/140)	0.101	(87/859)	0.35	**0.02**	
2DL3	***C2 +|+***	0.948	(184/194)	0.909	(1560/1716)	1.98	**0.04**	**0.03**	0.926	(50/54)	0.919	(788/857)	1.17	0.77	0.60	0.957	(134/140)	0.899	(772/859)	2.85	**0.02**	**0.02**
	C2 +|-	0.896	(1139/1271)	0.912	(10537/11557)	0.89	0.23		0.889	(399/449)	0.913	(5270/5775)	0.81	0.18		0.900	(740/822)	0.911	(5267/5782)	0.93	0.58	

OR = odds ratio. CO = control, p = p-value, Prop = proportion, Int.p = KIRxHLA interaction term p-value

<u>Supplementary Table 11:</u> Statistical interactions between biologically interacting KIRs and HLA alleles reaching statistical significance (p<0.05) in the meta-analysis cohort. Table shows KIR associations (+ dominant, ++ recessive) in individuals with or without a given HLA allele.

		Metanalysis					Cohort 1					Cohort 2				
**Gene**	**Subtype**	**SSc Prop. (Count)**	**CO Prop. (Count)**	**OR**	**p**	**Int.p**	**SScProp. (Count)**	**CO Prop. (Count)**	**OR**	**p**	**Int.p**	**SSc Prop. (Count)**	**CO Prop. (Count)**	**OR**	**p**	**Int.p**
3DS1++	***HLA-B*38+ (Bw4)***	0.100 (6/60)	0.024 (8/333)	3.92	**0.02**	**0.02**	0.071 (1/14)	0.019 (3/155)	5.23	0.21	0.09	0.109 (5/46)	0.028 (5/178)	3.49	0.08	0.14
	HLA-B*38- (Bw4)	0.038 (53/1405)	0.047 (610/12940)	0.75	**0.05**		0.022 (11/489)	0.049 (315/6477)	0.44	**0.008**		0.046 (42/916)	0.046 (295/6463)	0.94	0.70	
3DL1	***HLA-B*38+ (Bw4)***	0.900 (54/60)	0.976 (325/333)	0.26	**0.02**	**0.03**	0.929 (13/14)	0.981 (152/155)	0.19	0.21	0.15	0.891 (41/46)	0.972 (173/178)	0.29	0.08	0.15
	HLA-B*38- (Bw4)	0.957 (1345/1405)	0.952 (12314/12940)	1.20	0.19		0.967 (473/489)	0.950 (6153/6477)	1.60	0.07		0.952 (872/916)	0.953 (6161/6463)	1.05	0.77	
2DS4WT++	***HLA-C*16+ (C1/C2)***	0.108 (8/74)	0.042 (49/1157)	2.65	**0.02**	**0.007**	0.138 (4/29)	0.038 (23/602)	3.52	**0.04**	**0.02**	0.089 (4/45)	0.047 (26/555)	2.21	0.19	0.12
	HLA-C*16- (C1/C2)	0.044 (61/1391)	0.048 (585/12116)	0.87	0.31		0.051 (24/474)	0.053 (320/6030)	0.90	0.62		0.040 (37/917)	0.044 (265/6086)	0.90	0.57	
2DS2++	***HLA-C*16+ (C1/C2)***	0.203 (15/74)	0.098 (113/1157)	2.29	**0.009**	**0.02**	0.172 (5/29)	0.098 (59/602)	2.03	0.18	0.43	0.222 (10/45)	0.097 (54/555)	2.65	**0.02**	**0.01**
	HLA-C*16- (C1/C2)	0.091 (127/1391)	0.088 (1064/12116)	0.99	0.92		0.103 (49/474)	0.086 (516/6030)	1.18	0.30		0.085 (78/917)	0.090 (548/6086)	0.89	0.37	
2DL2++	***HLA-C*16+ (C1/C2)***	0.203 (15/74)	0.098 (113/1157)	2.29	**0.009**	**0.02**	0.172 (5/29)	0.098 (59/602)	2.03	0.18	0.43	0.222 (10/45)	0.097 (54/555)	2.65	**0.02**	**0.01**
	HLA-C*16- (C1/C2)	0.091 (127/1391)	0.088 (1063/12116)	0.99	0.92		0.103 (49/474)	0.086 (516/6030)	1.18	0.30		0.085 (78/917)	0.090 (547/6086)	0.89	0.37	
2DL3	***HLA-C*16+ (C1/C2)***	0.797 (59/74)	0.902 (1044/1157)	0.44	**0.009**	**0.02**	0.828 (24/29)	0.902 (543/602)	0.49	0.18	0.43	0.778 (35/45)	0.903 (501/555)	0.38	**0.02**	**0.01**
	HLA-C*16- (C1/C2)	0.909 (1264/1391)	0.912 (11053/12116)	1.01	0.93		0.897 (425/474)	0.915 (5515/6030)	0.85	0.30		0.915 (839/917)	0.910 (5538/6086)	1.12	0.37	
2DL1++	***HLA-C*16+ (C1/C2)***	0.608 (45/74)	0.707 (818/1157)	0.66	0.10	**0.03**	0.655 (19/29)	0.713 (429/602)	0.85	0.69	0.55	0.578 (26/45)	0.701 (389/555)	0.59	0.11	**0.03**
	HLA-C*16- (C1/C2)	0.730 (1015/1391)	0.704 (8529/12116)	1.15	**0.03**		0.703 (333/474)	0.696 (4196/6030)	1.04	0.74		0.744 (682/917)	0.712 (4333/6086)	1.21	**0.02**	
2DL3	***HLA-C*04+ (C2)***	0.931 (324/348)	0.904 (2104/2327)	1.61	**0.04**	**0.01**	0.915 (97/106)	0.912 (1060/1162)	1.10	0.79	0.34	0.938 (227/242)	0.896 (1044/1165)	2.10	**0.01**	**0.01**
	HLA-C*04- (C2)	0.894 (999/1117)	0.913 (9993/10946)	0.84	0.10		0.887 (352/397)	0.914 (4998/5470)	0.76	0.11		0.899 (647/720)	0.912 (4995/5476)	0.88	0.36	
2DL2++	***HLA-C*04+ (C2)***	0.069 (24/348)	0.095 (222/2327)	0.62	**0.04**	**0.01**	0.085 (9/106)	0.088 (102/1162)	0.91	0.79	0.34	0.062 (15/242)	0.103 (120/1165)	0.48	**0.01**	**0.01**
	HLA-C*04- (C2)	0.106 (118/1117)	0.087 (954/10946)	1.19	0.10		0.113 (45/397)	0.086 (473/5470)	1.30	0.11		0.101 (73/720)	0.088 (481/5476)	1.13	0.36	
2DL1++	***HLA-C*04+ (C2)***	0.770 (268/348)	0.706 (1642/2327)	1.45	**0.006**	**0.03**	0.736 (78/106)	0.700 (813/1162)	1.20	0.44	0.38	0.785 (190/242)	0.712 (829/1165)	1.61	**0.006**	**0.04**
	HLA-C*04- (C2)	0.709 (792/1117)	0.704 (7705/10946)	1.04	0.59		0.690 (274/397)	0.697 (3812/5470)	0.97	0.81		0.719 (518/720)	0.711 (3893/5476)	1.06	0.49	
2DS4WT++	***HLA-C*07+ (C1)***	0.055 (45/814)	0.044 (322/7336)	1.25	0.17	**0.02**	0.062 (19/304)	0.049 (180/3681)	1.23	0.41	0.24	0.051 (26/510)	0.039 (142/3655)	1.34	0.19	**0.04**
	HLA-C*07- (C1)	0.037 (24/651)	0.053 (312/5937)	0.66	**0.05**		0.045 (9/199)	0.055 (163/2951)	0.76	0.44		0.033 (15/452)	0.050 (149/2986)	0.63	0.11	
3DS1++	***HLA-A*24+ (Bw4)***	0.073 (20/274)	0.055 (115/2094)	1.29	0.33	**0.04**	0.091 (9/99)	0.059 (62/1046)	1.56	0.24	**0.001**	0.063 (11/175)	0.051 (53/1048)	1.16	0.68	0.67
	HLA-A*24- (Bw4)	0.033 (39/1191)	0.045 (503/11179)	0.68	0.02		0.007 (3/404)	0.046 (256/5586)	0.15	**0.001**		0.046 (36/787)	0.044 (247/5593)	0.96	0.81	

OR = odds ratio. CO = control, p = p-value, Prop. = proportion, Int.p = KIRxHLA interaction term p-value
