# Supplementary material for: Contribution of HLA and KIR Alleles to Systemic Sclerosis Susceptibility and Immunological and Clinical Disease Subtypes
Source: Front Genet. 2022 Jun 8;13:913196. doi: 10.3389/fgene.2022.913196 (PMC9214260; doi:10.3389/fgene.2022.913196)
Supplement: Supplementary file 2 [file DataSheet1.PDF]

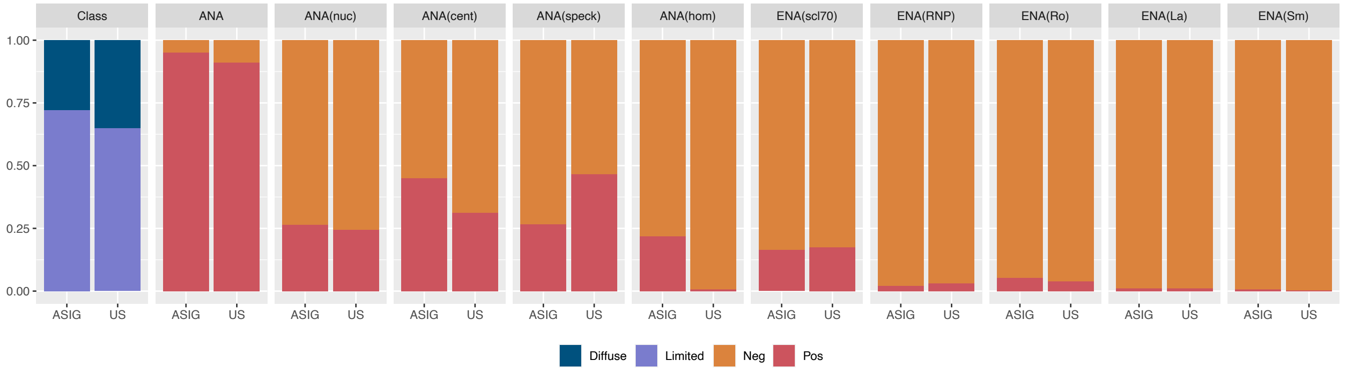

<u>Supplementary Figure 1:</u> Proportion of Cohort 1 (ASIG) and Cohort 2 (US) SSc patients within each disease subclassification and positive or negative for clinical autoantibodies. ANA, antinuclear autoantibody; nuc, nucleolar staining; cent, centromere staining; speck, speckled staining; hom, homogeneous staining; ENA, extractable nuclear antigen autoantibody; scl70, anti-Scl70/anti-topoisomerase autoantibiody; RNP, anti-ribonucleoprotein autoantibody; Ro, anti-Sjögren's-syndrome related antigen A autoantibody (anti-SSA/Ro); La, anti-Sjögren's-syndrome related antigen B autoantibody (anti-SSB/La); Sm, anti-Smith autoantibody.

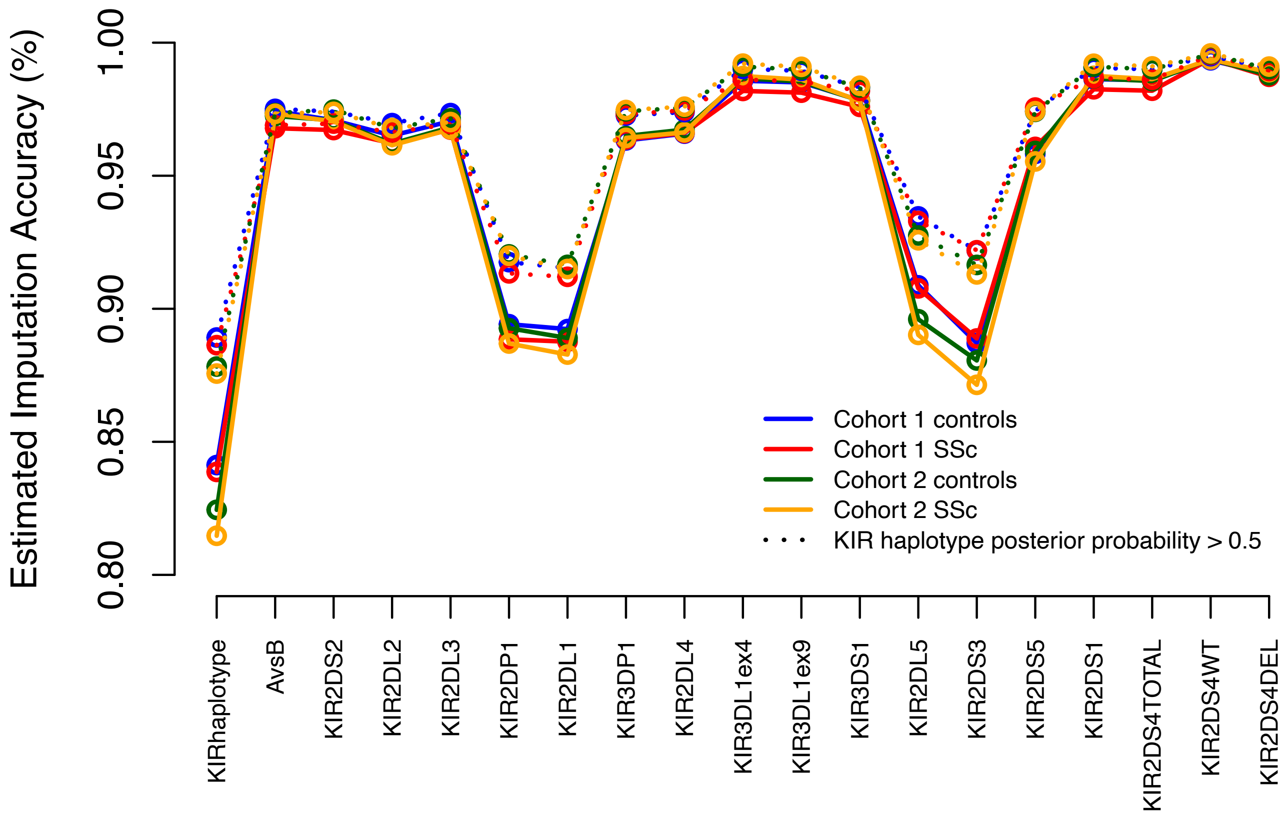

<u>Supplementary Figure 2:</u> Estimated imputation accuracy (posterior probability) scores for individual KIR genes and imputed KIR haplotypes across SSc patients and controls from both study cohorts. Dashed lines indicate the effect of filtering included haplotypes using a *KIRhaplotype* posterior probability threshold of >0.5 on gene-based imputation accuracy.

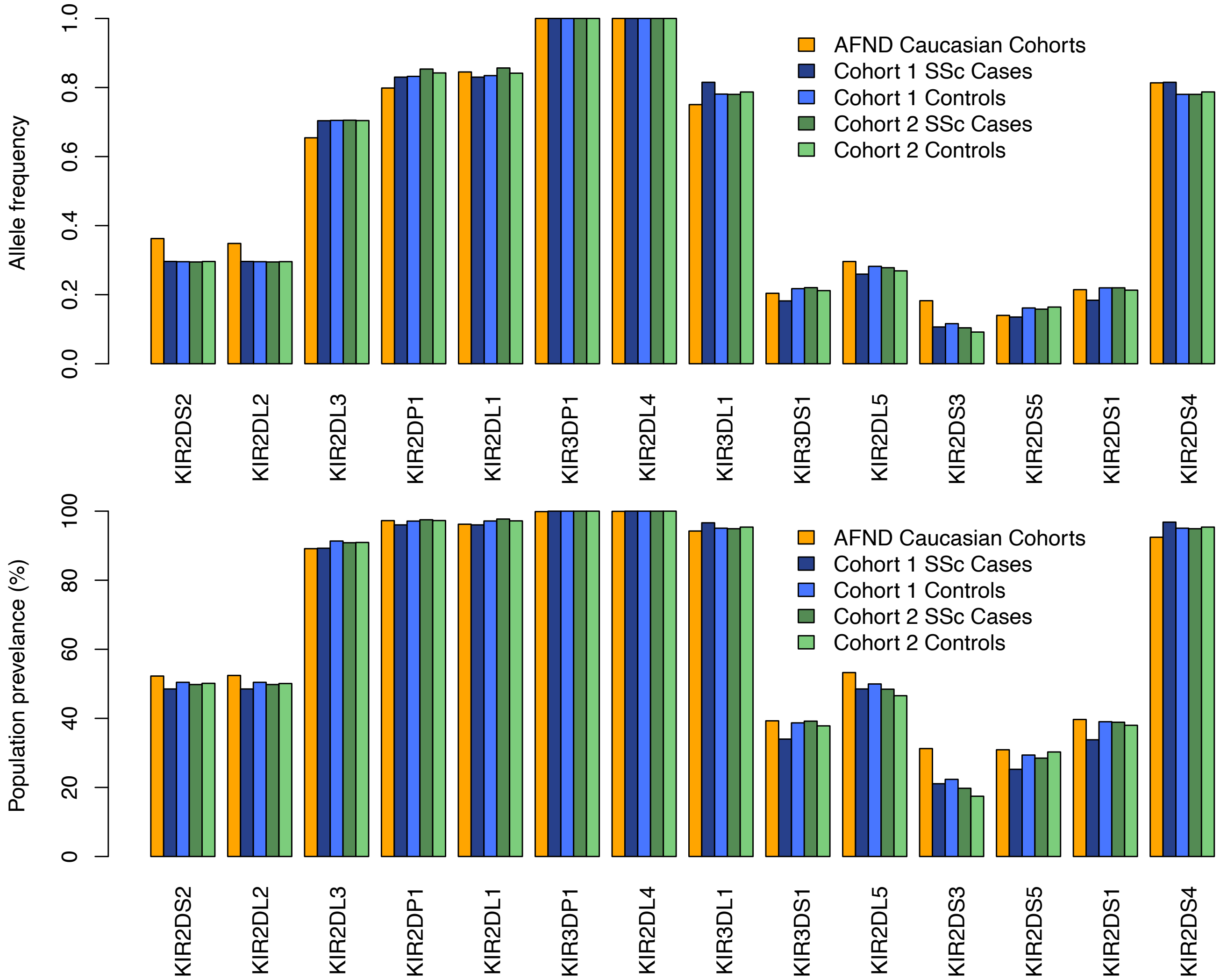

Supplementary Figure 3: Comparison of imputed KIR frequencies (A) and population prevalence (B) in Cohort 1 (blue) and Cohort 2 (green) relative to those averaged across all available Caucasian populations with KIR allele frequencies and % population prevalence available in the Allele Frequency Net Database (AFND).

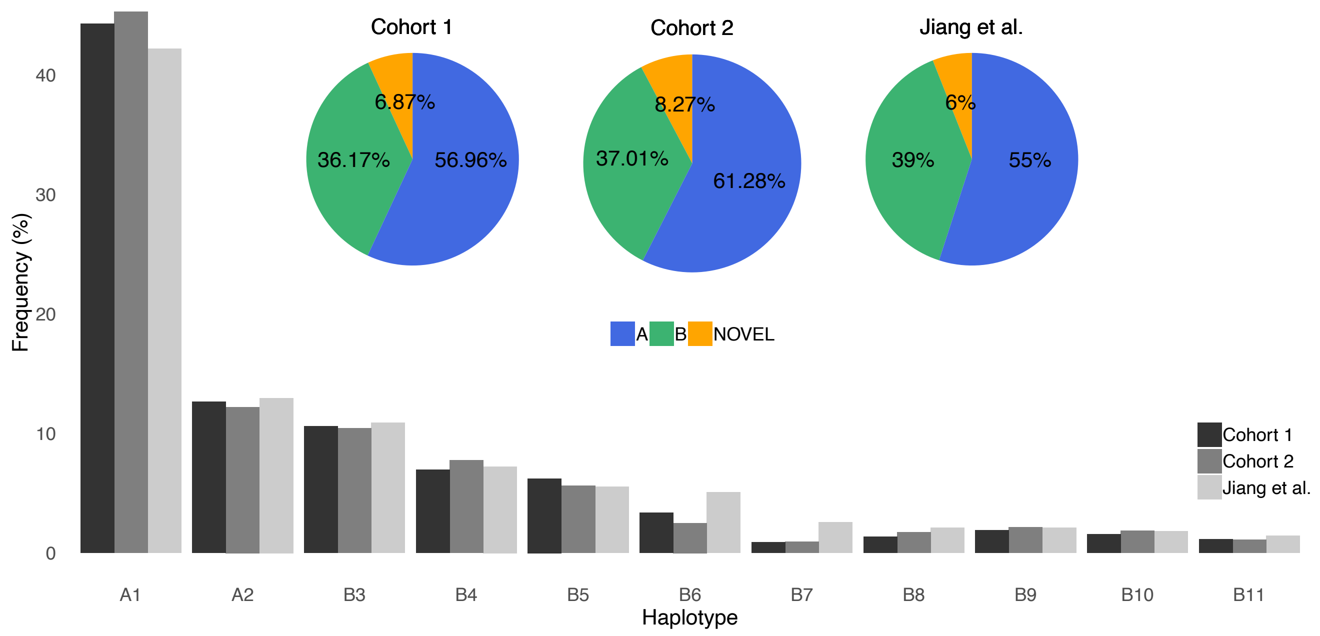

<u>Supplementary Figure 4:</u> Comparison of imputed KIR haplotype frequencies in Cohorts 1 and 2 relative to those derived from 793 nuclear families from the US and UK as published in Jiang et.al., 2012.
